# Rational Design of Capsid Protein VP1 Degraders to Overcome Pleconaril Resistance in Inhibiting Enterovirus D68

**DOI:** 10.1021/jacsau.6c00039

**Published:** 2026-03-23

**Authors:** Kan Li, Jiabin Ni, Guangjin Fan, Jun Wang

**Affiliations:** Department of Medicinal Chemistry, Ernest Mario School of Pharmacy, Rutgers, The State University of New Jersey, Piscataway, New Jersey 08854, United States

**Keywords:** enterovirus, EV-D68, capsid protein, PROTAC, antiviral

## Abstract

Enterovirus D68 (EV-D68) is an emerging respiratory pathogen with pandemic potential, yet no vaccines or antivirals are available. Capsid inhibitors, such as pleconaril, that target the hydrophobic canyon on the viral capsid protein VP1, exhibit potent antiviral activity but have a low barrier to resistance. Here, we report a targeted protein degradation strategy to overcome antiviral resistance by selectively degrading the capsid protein VP1. Through a structure-based rational design, we developed a series of pleconaril-based PROTACs that recruit the cereblon (CRBN) E3 ligase to degrade the capsid VP1 protein. We established a single-cycle replication assay integrating immunofluorescence and Western blot to quantify VP1 protein levels when compounds were added postviral entry. Linker and CRBN ligand optimization led to the identification of **Jun15702**, a first-in-class VP1 degrader that displays potent antiviral activity against multiple wild-type EV-D68 strains and retains submicromolar potency against recombinant pleconaril-resistant variant, rMO-VP1 F159 V. Significantly, **Jun15702** displayed a higher genetic barrier to drug resistance than pleconaril. Mechanistic studies demonstrate that **Jun15702** not only inhibits viral entry, as does pleconaril, but also induces CRBN-dependent degradation of VP1 when added post viral entry. Collectively, this work demonstrates targeted degradation of a viral structural protein as a viable strategy and introduces a new paradigm for overcoming drug resistance in antiviral discovery against enteroviruses.

## Graphical abstract

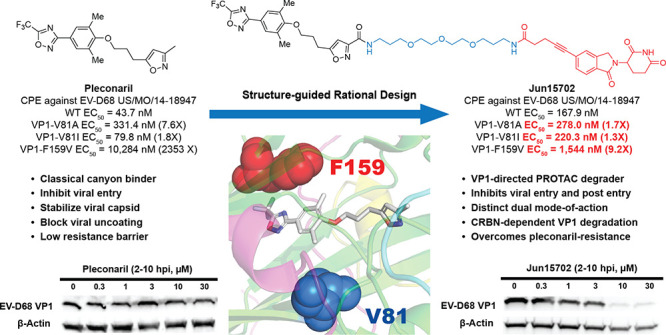

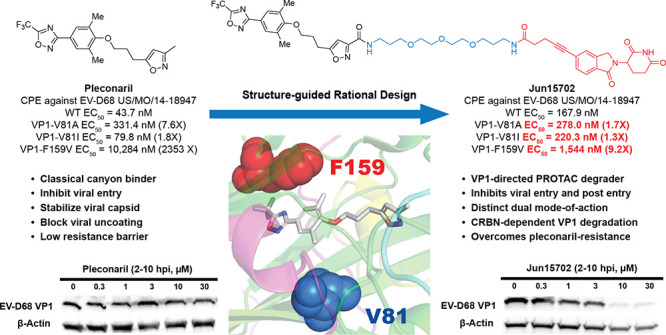

## Introduction

Enterovirus D68 (EV-D68) is a respiratory virus that primarily causes mild to moderate respiratory diseases in children. However, severe infections of EV-D68 can cause neurological complications, including acute flaccid myelitis (AFM), resulting in limb weakness and, in rare cases, death.^1–3^ EV-D68 is transmitted mainly through the respiratory route and has emerged as a significant public health concern with pandemic potential.^4^ During its first major outbreak in 2014, thousands of confirmed infections and an estimated millions of undetected cases occurred in the United States and across Europe and Asia.^5–7^ Compared with historical strains, contemporary EV-D68 isolates from the 2014 outbreak onward exhibited increased pathogenicity.^6,8–10^ Notably, recent strains, such as EV-D68 (US/KY/14) and EV-D68 (US/MO/47), can infect neuronal cell lines and induce cytopathic effects, a property not observed for earlier strains, including the prototypical Fermon isolate.^10,11^ Despite its growing clinical impact, no antiviral agent has been approved for the prevention or treatment of EV-D68 infection, underscoring an urgent need for new therapeutic strategies.^12^

EV-D68 is a nonenveloped, positive-sense, single-stranded RNA virus enclosed by an icosahedral capsid composed of 60 protomers. Each protomer contains three external capsid proteins (VP1, VP2, VP3) and an internal protein (VP4).^12,13^ A prominent structural feature of the capsid proteins is the “canyon”, a deep surface depression primarily formed by the G-H loop of VP1 (Figure 1A).^13^ This hydrophobic pocket is typically occupied by a lipid molecule, which plays a critical role in capsid stability and host receptor engagement.^2,13^ The VP1 canyon has been extensively validated as an antiviral drug target, with capsid binders, such as pleconaril and its analog CP-11526092, shown to inhibit viral uncoating and genome release (Figure 1A).^13,14^ However, capsid inhibitors generally exhibit a low barrier to resistance.^15^ During the study with pleconaril, mutations at VP1 canyon residues, such as Val81 (V81A, V81I) and Phe159 (F159 V), were identified that confer resistance by disrupting ligand binding,^12,16–18^ thereby limiting its therapeutic potential against EV-D68 (Figure 1B).

**Figure 1:**
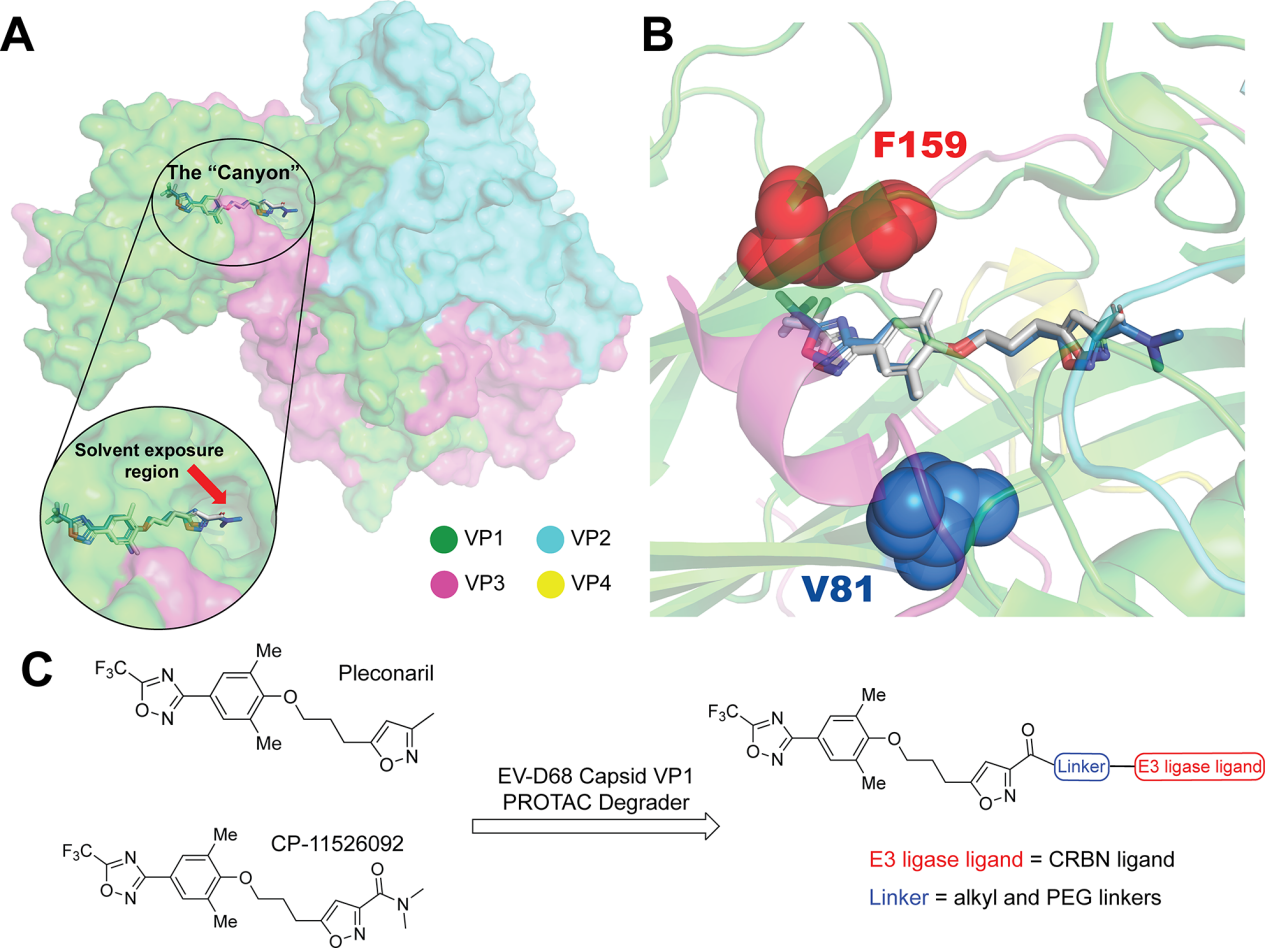
Design of EV-D68 capsid VP1 PROTACs. (A) Cryo-EM structures of EV-D68 capsid proteins in complex with pleconaril (PDB ID: 4WM7) and CP-11526092 (PDB ID: 7TAF). Capsid proteins VP1, VP2, VP3, and VP4 are colored green, cyan, purple, and yellow, respectively. The VP1 canyon occupied by capsid binders and the solvent-exposed region are highlighted. (B) Pleconaril-resistance–associated VP1 residues Val81 and Phe159 are shown as marine and red spheres, respectively. (C) Design strategy for VP1 PROTACs derived from pleconaril and CP-11526092, in which CRBN ligands are tethered to the isoxazole moiety via alkyl or PEG linkers.

Proteolysis-targeting chimeras (PROTACs) are bifunctional molecules that direct target proteins to the ubiquitin-proteasome pathway for degradation.^19–21^ PROTACs offer several advantages, including catalytic target protein depletion and the potential to overcome resistance mechanisms associated with target overexpression or point mutations.^22,23^ This paradigm-shifting strategy has rapidly advanced in oncology, leading to the development of numerous PROTACs that have progressed into clinical trials.^24,25^ In contrast, antiviral PROTACs remain at an early stage of development.^26,27^ Nonetheless, proof-of-concept studies have demonstrated successful applications of targeted protein degradation against several viral nonstructural proteins, including the NS3/4A protease of hepatitis C virus (HCV) and the main protease of SARS-CoV-2, as well as viral structural proteins such as the dengue virus envelope protein.^28–31^ These advances prompted us to explore a degradation-based strategy targeting the EV-D68 capsid protein VP1 (Figure 1C).

Here, we report the rational design of pleconaril-based VP1 PROTAC degraders that recruit the cereblon (CRBN) E3 ligase to induce selective VP1 degradation (Figure 1C). As capsid inhibitors primarily act during viral entry (0–1 h-postinfection, hpi), we established experimental approaches to directly assess VP1 depletion by PROTACs during a single replication cycle. VP1 degradation was evaluated by integrating immunofluorescence and Western blot analyses. Our strategy led to the identification of **Jun15702**, a first-in-class VP1 degrader with potent antiviral activity against multiple EV-D68 strains and submicromolar activity against pleconaril-resistant variants. Furthermore, **Jun15702** displays a higher genetic barrier to drug resistance than pleconaril.

## Results and Discussion

### Design of VP1 PROTACs Based on Pleconaril and Pomalidomide (4-amino thalidomide)

Based on the reported Cryo-EM structures of EV-D68 capsid proteins in complex with pleconaril or CP-11526092, as well as the structure–activity relationship studies of pleconaril, the 3-methyl substitution at the isoxazole ring is amenable to conjugation without impairing the VP1 binding (Figure 1A,C).^13,14^ We therefore chose the isoxazole ring as the exit site for linking to E3 ligase ligands via linkers of various lengths and hydrophobicity. We initially selected pomalidomide, a CRBN ligand that binds the substrate receptor of the CUL4-RBX1-DDB1-CRBN E3 ubiquitin ligase complex (CRL4CRBN). We synthesized a series of PROTACs and evaluated their antiviral activities against wild-type (WT) EV-D68 US/MO/14–18947 in a cytopathic effect (CPE) assay (Table 1). Candidate degraders with alkyl and polyethylene glycol (PEG) linkers of different lengths were tested. PROTACs with alkyl linkers (**Jun1522**, **Jun14956**, **Jun1554**, and **Jun15191**) showed no antiviral activity up to 5 μM (EC_50_ > 5 μM). In contrast, PROTACs with PEG linkers retained potent antiviral activity. For example, **Jun15183**, with a PEG2-amine linker, had potent antiviral activity (EC_50_ = 110.2 nM). PROTACs with longer linkers generally showed improved antiviral activity (**Jun15375** vs **Jun15332** and **Jun15333**, **Jun15322** vs **Jun15323** and **Jun15331**). These results support our hypothesis that pleconaril can be derivatized without substantially compromising its antiviral activity.

**Table 1:**
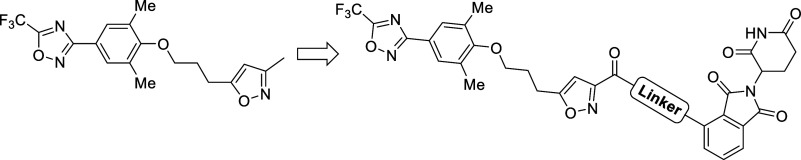

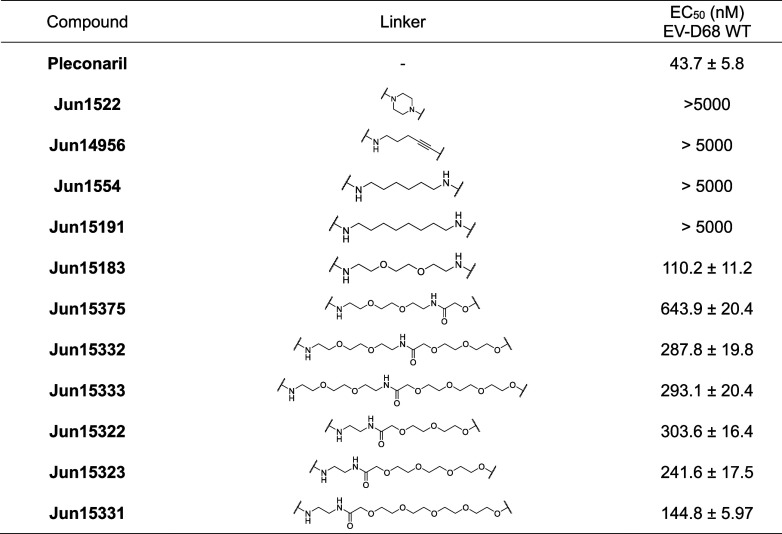
Initial Exploration of Pleconaril-Based VP1 PROTACs^a^

a EC_50_ values (mean ± SD) were determined by CPE assay against EV-D68 US/MO/14–18947 in three biological replicates (*n* = 3).

### Design of VP1 PROTACs Based on Pleconaril and 5-amino-thalidomide

Starting from **Jun15183**, we also explored the PROTAC design by linking pleconaril to 5-amino-thalidomide. Interestingly, **Jun15192** exhibited an improved antiviral activity against the WT EV-D68 US/MO/14–18947 (EC_50_ = 49.9 nM) (Table 2). Thus, **Jun15192** was selected as a starting point for further optimization and characterization. We synthesized and tested additional analogs of **Jun15192** bearing PEG linkers of varying lengths or modified propoxylene-PEG linkers (Table 2). As the goal of designing VP1 PROTACs is to target pleconaril-resistant mutant viruses, we tested the antiviral activity of these PROTACs against the WT EV-D68 US/MO/14–18947 virus, along with three recombinant EV-D68 US/MO/14–18947 (rMO) strains containing pleconaril-resistance mutations in VP1, namely rMO-VP1 V81A, rMO-VP1 V81I, and rMO-VP1 F159 V (Figure 1B). These mutations were identified from prior serial viral passage experiments with escalating drug selection pressure.^13,16,18,32^ Compared to the WT strain, pleconaril exhibited 7.6-fold resistance against rMO-VP1 V81A, 1.8-fold resistance against rMO-VP1 V81I, and 235.3-fold resistance against rMO-VP1 F159 V (Table 2). **Jun15373**, with a shorter linker, displayed an antiviral profile similar to that of pleconaril against the WT virus and the V81A (5.1-fold) and V81I (1.6-fold) variants (Table 2). Compounds with extended linkers than **Jun15192**, including **Jun15294**, **Jun15374**, and **Jun15421**, had reduced antiviral activity against both WT and the drug-resistant mutants rMO-VP1 V81A and rMO-VP1 V81I (Table 2). Disappointingly, none of the analogs showed inhibition against the rMO-VP1 F159 V variant at up to 20 μM (Table 2).

**Table 2:**
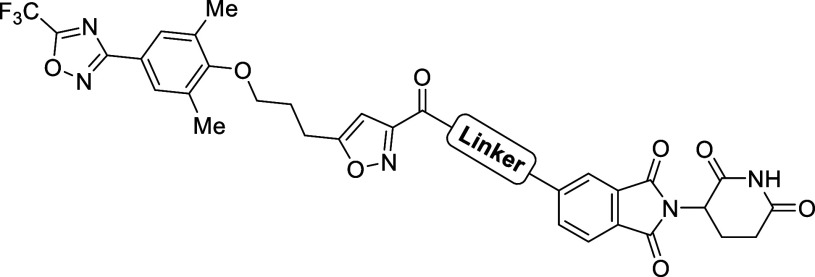

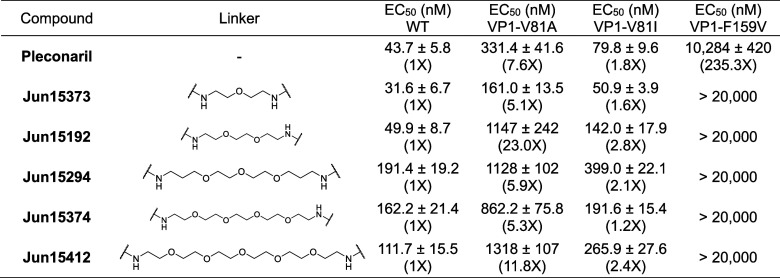
Linker Optimization of VP1 PROTACs^a^

a EC_50_ values (mean ± SD) were determined by CPE assay against EV-D68 US/MO/14–18947 and the recombinant viruses in three biological replicates (*n* = 3).

### CRBN Ligand Optimization of VP1 PROTACs

We next focused on optimizing the CRBN ligand to enhance antiviral potency and overcome resistance. We first modified the 5-amino-thalidomide scaffold; however, introduction of a fluorine substituent on the phenyl ring (**Jun15616**) failed to improve antiviral activity against the three pleconaril-resistant strains, especially rMO-VP1 F159 V variant (Table 3). We then altered the linker-CRBN conjugation strategy, replacing the carbon–nitrogen linkage with an amide bond. The resulting degrader, **Jun15701**, showed reduced antiviral activity against the WT virus compared to pleconaril but exhibited similar activity against the highly resistant rMO-VP1 F159 V variant (EC_50_ = 11,818 nM vs 10,284 nM for pleconaril).

**Table 3:**
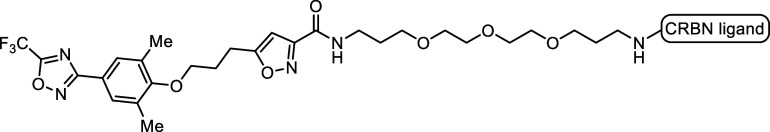

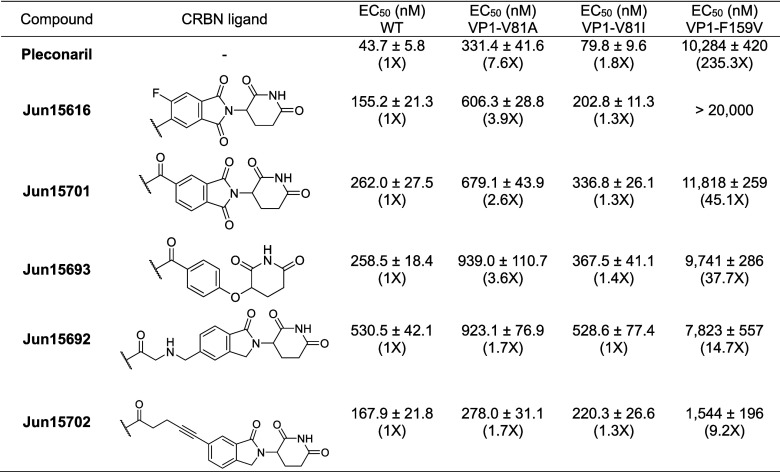
CRBN Ligands Optimization of VP1 PROTAC Degraders^a^

a EC_50_ values (mean ± SD) were determined by CPE assay against EV-D68 US/MO/14–18947 and the recombinant viruses in three biological replicates (*n* = 3).

Building on this conjugation approach, we evaluated additional reported CRBN ligands, including **Jun15693** (a benzoic acid-derived glutarimide), **Jun15692** (a glycine-linked lenalidomide-C1 amine), and **Jun15702** (a lenalidomide-acetylene-C2-carboxylic acid derivative). These three candidate VP1 PROTACs demonstrated improved antiviral activity against the highly resistant rMO-VP1 F159 V (Table 3). Encouragingly, **Jun15702** emerged as the most promising candidate, exhibiting equivalent potency against the rMO-VP1 V81A and V81I variants and a 9.2-fold loss of activity against the rMO-VP1 F159 V mutant compared to the WT virus. **Jun15702** achieved submicromolar potency against rMO-VP1 F159 V, representing approximately a 6-fold improvement over pleconaril (EC_50_ = 1544 nM vs 10,284 nM for pleconaril; Table 3).

To evaluate the broad-spectrum antiviral activity of **Jun15702**, we assessed its antiviral activity against five EV-D68 strains isolated during the 2014 outbreak using plaque reduction assays, with pleconaril included as a positive control. The virus panel consisted of clade B1 strains US/MO/14–18947 and US/MO/14–18949, clade B2 strains US/IL/14–18,952 and US/IL/14–18956, and the clade D strain US/KY/14–18953. **Jun15702** demonstrated robust and consistent antiviral activity across all tested strains, with EC_50_ values ranging from 65.4 to 564.5 nM. Notably, **Jun15702** was more potent than pleconaril against all strains except EV-D68 US/MO/14–18947 (Figure 2).

**Figure 2:**
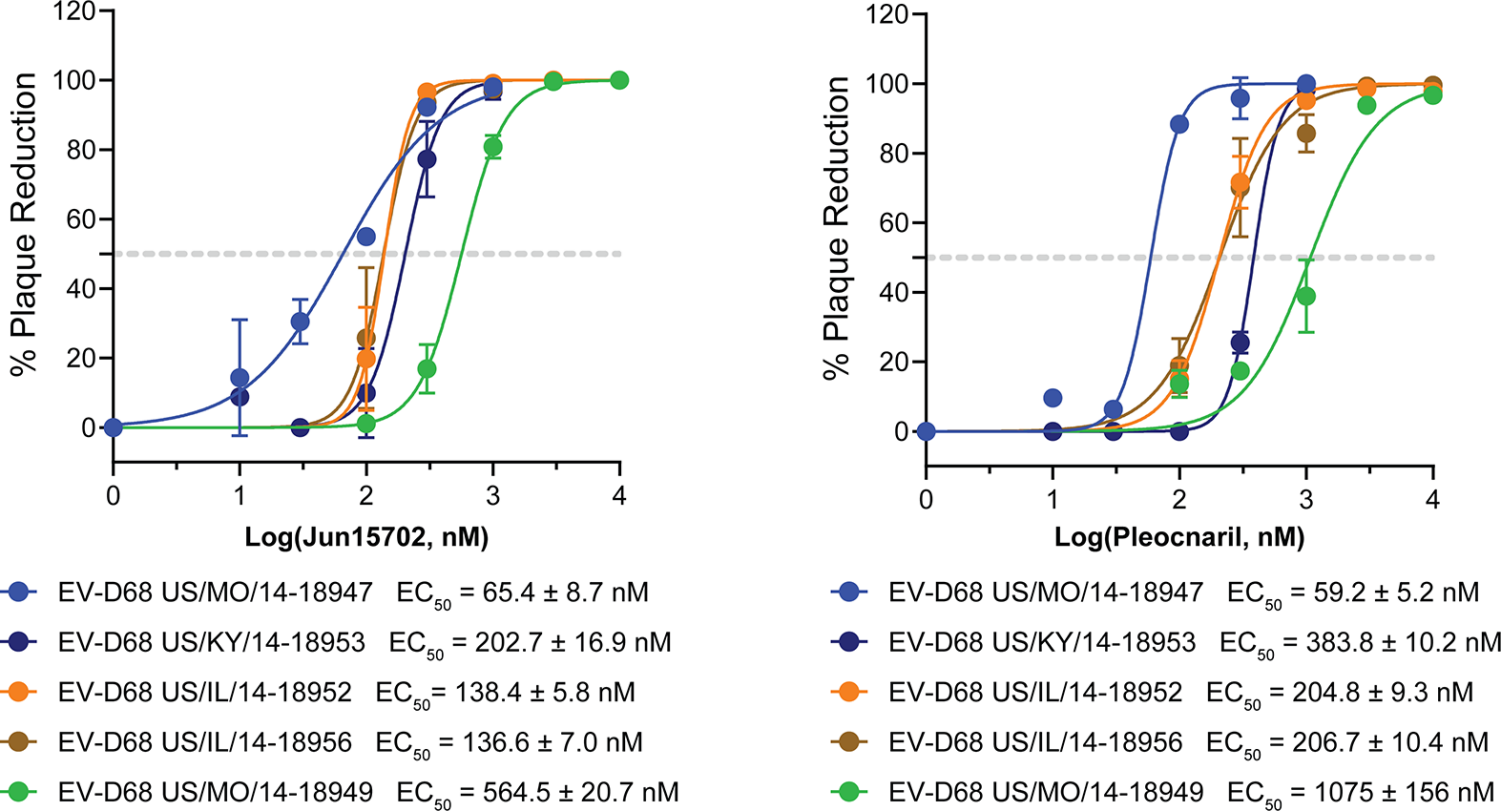
**Jun15702** inhibits multiple EV-D68 strains. Antiviral activity of **Jun15702** and pleconaril against five EV-D68 strains as determined by plaque assays. EC_50_ values are reported as mean ± SD from two biological replicates.

### Synthesis of VP1 PROTACs

The synthesis of pleconaril derivatives was carried out following reported procedures with minor modifications.^33^ *O*-alkylation of hydroxybenzonitrile **1a** with 5-bromo-1-pentyne in the presence of potassium carbonate and potassium iodide in *N*-methylpyrrolidone at 65 °C afforded 4-(pent-4-yn-1-yloxy)­benzonitrile **1b** (Scheme 1A). Subsequent treatment of **1b** with hydroxylamine hydrochloride and potassium carbonate in ethanol yielded the corresponding amidoxime **1c**. Two sequential cyclization reactions were then employed to construct the heterocyclic core: treatment with trifluoroacetic anhydride in pyridine generated the 1,2,4-oxadiazole intermediate **1d**, followed by reaction with 2-chloro-2-(hydroxyimino)­acetic acid methyl ester in the presence of triethylamine in dry dimethylformamide to afford the isoxazole derivative **1e**. Final ester hydrolysis using lithium hydroxide provided the key intermediate carboxylic acid **1** (Scheme 1A). A series of *tert*-butoxycarbonyl (Boc)-protected amino PEG or alkyl linkers were coupled to intermediate **1** via amide bond formation to give intermediates **2a**–**2g**. Subsequent one-step Boc deprotection furnished the corresponding amine intermediates **4a**-**4g** (Scheme 1B).

**Scheme 1:**
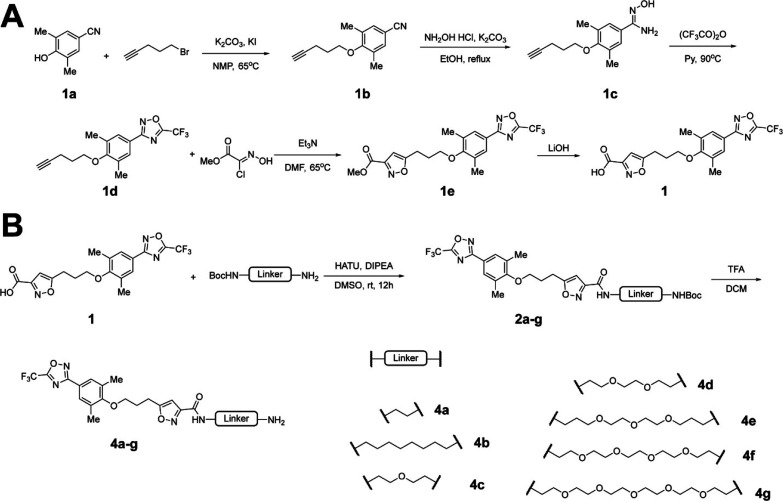
Synthesis of Reported Intermediates Acid **1** and Intermediates **4a** to **4g**

In parallel, commercially available cereblon (CRBN) ligands were functionalized with alkyl or PEG linkers to generate additional coupling partners for PROTAC assembly. Nucleophilic substitution of 2-(2,6-dioxopiperidin-3-yl)-4-fluoroisoindoline-1,3-dione with Boc-protected amino PEG or alkyl linkers afforded intermediates **3a** and **3b**, which upon TFA-mediated Boc deprotection yielded amine intermediates **5a** and **5b** (Scheme 2A). Additional CRBN-linker intermediates, including thalidomide-acetylene-C3-NH_2_ (**5c**), lenalidomide-acetylene-C2-COOH (**5d**), and the corresponding N-methylated negative control (**5e**), were synthesized from commercially available CRBN ligands via single-step cross-coupling reactions followed by TFA-mediated Boc or *tert*-butyl deprotection (Scheme 2B).

**Scheme 2:**
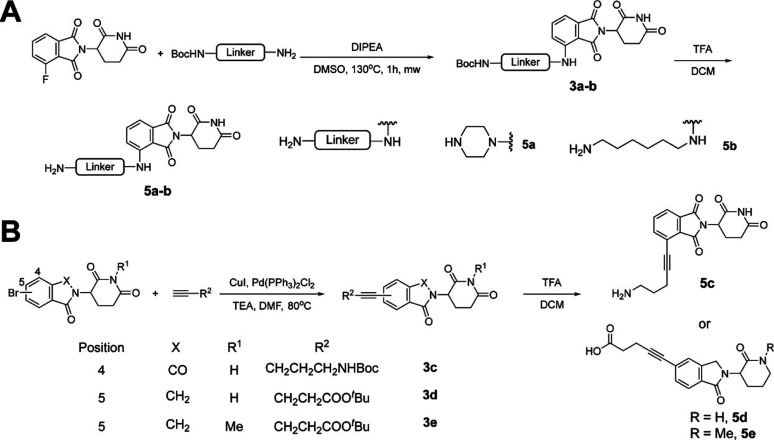
Synthesis of Intermediates **5a** to **5e**

Final VP1 PROTAC degraders were assembled through three general synthetic strategies (Scheme 3). First, amide coupling of the pleconaril-derived acid intermediate 1 with PEG- or alkyl-linked CRBN ligands **5a**–**5c** yielded a subset of PROTACs (Scheme 3A). Second, amide coupling of pleconaril-derived amine intermediates **4a**, **4d**, and **4e** with either commercially available CRBN ligands or linker-functionalized intermediates **5d** and **5e** furnished additional analogs (Scheme 3B). Finally, nucleophilic substitution reactions between amine intermediates **4b**–**4g** and commercially available CRBN ligands were employed to generate the remaining VP1 PROTAC degraders (Scheme 3C).

**Scheme 3:**
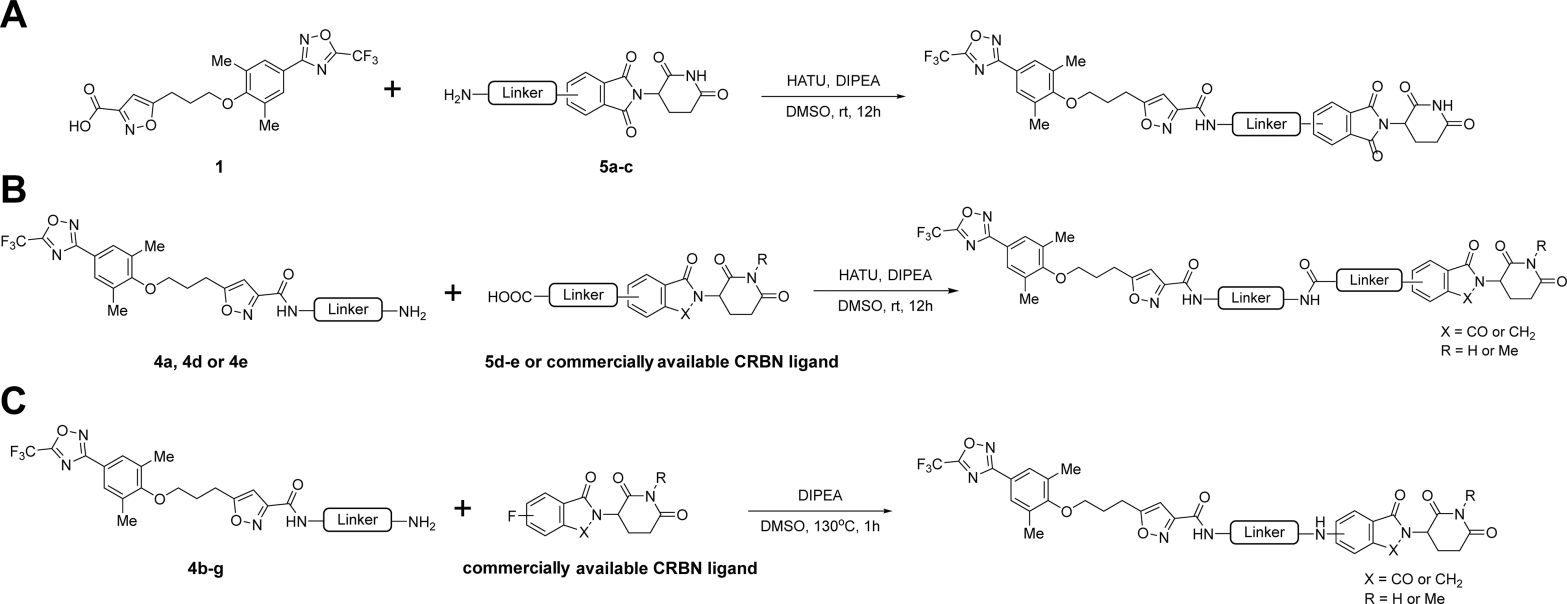
Synthesis of VP1 PROTAC Degraders

### Characterization of VP1 Degradation by PROTACs Using Immunofluorescence Assay

If **Jun15702** functions as a PROTAC to degrade VP1, it is expected to retain its antiviral activity when added postviral entry. This is in direct contrast to VP1 inhibitors, such as pleconaril, which act at viral entry and lose antiviral activity when added post viral entry.^16^ To test this hypothesis, we performed a time-of-addition experiment with a single cycle of viral replication (8 h) and quantified viral VP1 levels using immunofluorescence. In the time-of-addition experiment, the compound was added at different time points before, during, or after viral infection, and VP1 levels were quantified by immunofluorescence at 8 hpi. As expected, pleconaril almost completely suppressed VP1 expression when added before (−1–8 hpi) or during (0–1 hpi, 0–8 hpi) viral entry but lost antiviral activity when added after viral entry (2–8 hpi) (Figures 3A and S1A).

**Figure 3:**
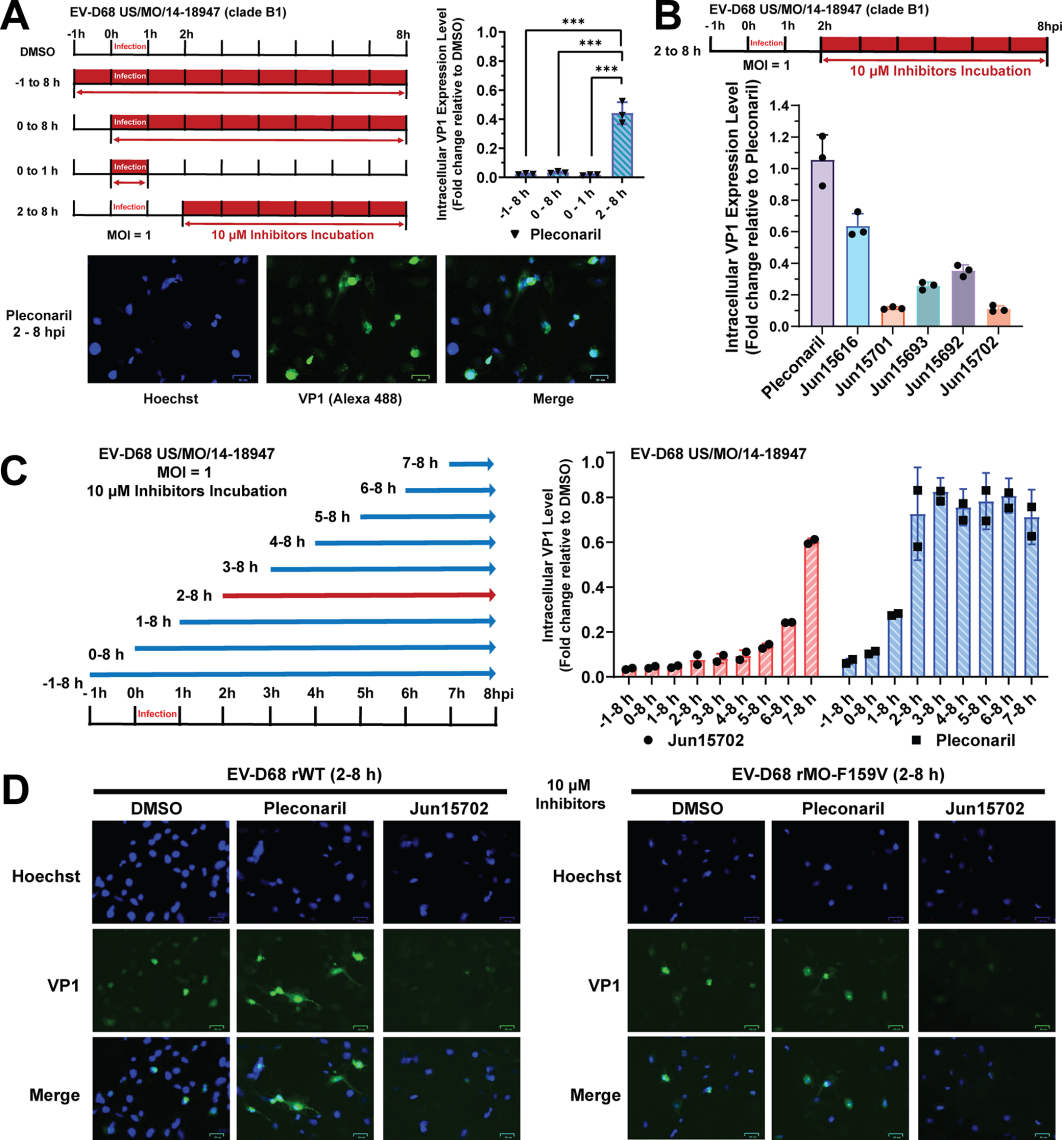
Immunofluorescence assay with a single-cycle EV-D68 replication elucidates **Jun15702**-induced VP1 degradation. (A) Time-of-addition assay with pleconaril (*n* = 3, mean ± SD, three independent experiments). VP1 levels are expressed as fold change relative to the DMSO-treated control. VP1 was detected using an Alexa Fluor 488-conjugated secondary antibody (green), and nuclei were counterstained with Hoechst (blue). Representative images from three independent experiments are shown. (B) Immunofluorescence assay of VP1 following treatment with VP1 PROTACs at 2 hpi (2–8 h) (*n* = 3, mean ± SD, three independent experiments). (C) Schematic representation and results for the full course of the time-of-addition assay. Quantification of intracellular VP1 levels by immunofluorescence staining following treatment with **Jun15702** or pleconaril at the indicated time points (*n* = 2 independent experiments). VP1 levels are expressed as fold change relative to the DMSO-treated control. (D) Immunofluorescence images of VP1 levels following treating EV-D68 rWT or rMO-F159 V virus-infected RD cells with pleconaril or **Jun15702**. Compounds (10 μM) or DMSO were present in the cell culture for 2–8 h. Representative images from two independent experiments are shown. The scale bar in panels A and D is 25 μm.

To characterize PROTAC-mediated VP1 degradation, we performed immunofluorescence assays by adding PROTACs post viral entry at 2 hpi (Figure 3B). **Jun15616** induced a less than 2-fold reduction in VP1 levels compared with the pleconaril-treated control (Figure 3B). In contrast, **Jun15701**, **Jun15693**, and **Jun15692** induced substantial VP1 depletion, with 2.5- to 10-fold reductions compared with pleconaril-treated cells (Figures 3B and S1B). Notably, **Jun15702** produced the most pronounced effect, reducing intracellular VP1 levels by nearly 10-fold relative to pleconaril treatment (Figures 3B and S1B).

We next performed a full-course time-of-addition experiment to quantify intracellular viral VP1 protein levels using immunofluorescence imaging. In this experiment, RD cells were infected with EV-D68 US/MO/14–18947 at an MOI of 1, and 10 μM of **Jun15702** or pleconaril were added at different time points either before, during, or after viral infection throughout the replication cycle (Figure 3C). Both **Jun15702** and pleconaril showed nearly complete depletion of VP1 protein level when present before viral entry at −1–8 h and 0–8 h (Figure 3C). Pleconaril slightly lost inhibitory activity when added right after infection at 1 hpi (1–8 h), whereas **Jun15702** retained inhibitory activity (Figures 3C and S1C). The inhibitory activity of pleconaril dramatically diminished when it was added postviral entry at 2–8 h to 7–8 h. In contrast, **Jun15702** retained potent inhibitory activity even when treated at 5–8 h, and gradually lost activity at 6–8 h and 7–8 h. The results suggest that **Jun15702** displayed a different mode of action from pleconaril and could inhibit EV-D68 viral replication at the early, middle, and late stages of viral replication (Figures 3C and S1C).

As **Jun15702** displayed a submicromolar antiviral activity against pleconaril-resistant strain rMO-VP1 F159 V (Table 3), an immunofluorescent staining was performed to validate VP1 depletion (Figure 3D). Ten μM pleconaril or **Jun15702** was added to EV-D68 rWT or the rMO-F159 V mutant virus-infected RD cells at 2 hpi (2–8 h). The pleconaril-treated groups showed a high level of intracellular VP1 protein, similar to that in the DMSO group (Figure 3D), suggesting no inhibition. In contrast, **Jun15702**-treated groups showed dramatically reduced VP1 immunofluorescence signals, suggesting that **Jun15702** can degrade not only WT VP1 but also the VP1–F159 V mutant (Figure 3D). Collectively, the immunofluorescent assay results of **Jun15702** corroborate its potent antiviral activity against EV-D68 WT and mutant viruses.

### Characterization of the Mechanism of Action of CRBN-Mediated VP1 Degradation

To investigate whether VP1 degradation by this series of PROTACs is CRBN-dependent, the corresponding N-methylated control compounds **Jun15551** and **Jun15953** were synthesized and evaluated alongside **Jun15294** and **Jun15702** (Figure 4A). In CPE assays, modest potency reduction was observed for **Jun15551** (359.7 vs 191.4 nM for **Jun15294**, Figure 4A). **Jun15953** also exhibited approximately a 3-fold loss of antiviral potency relative to **Jun15702** (EC_50_ = 410 nM vs 167.9 nM; Figure 4B). In the immunofluorescence assay, both **Jun15294** and **Jun15702** reduced VP1 levels at 8 hpi by about 10-fold. In contrast, **Jun15551** exhibited a marked loss of VP1 degradation activity, with a slighter than 2-fold decrease in VP1 level compared to pleconaril, and **Jun15593** showed about a 4-fold decrease in VP1 level (Figure 4C).

**Figure 4:**
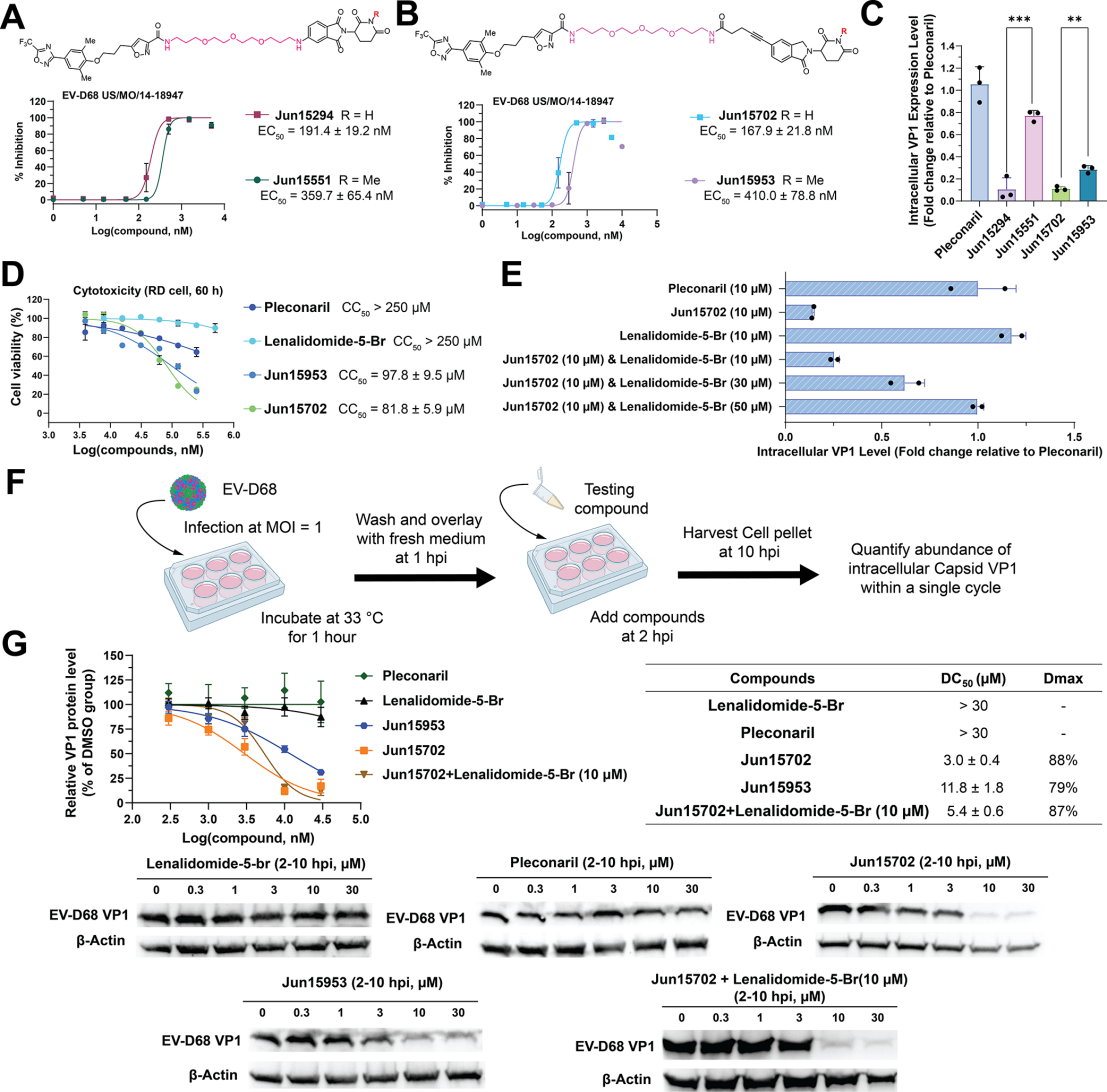
Jun15702 induces capsid VP1 degradation in a CRBN-dependent manner. (A) Chemical structures of the VP1 PROTAC degrader **Jun15702** and its corresponding N-methylated analog, **Jun15551**, and their antiviral activities. EC_50_ values are reported as mean ± SD from three biological replicates in CPE assays (*n* = 3). (B) Chemical structures of the VP1 PROTAC degrader **Jun15702** and its corresponding N-methylated analog, **Jun15953**, and their antiviral activities. EC_50_ values are reported as mean ± SD from three biological replicates in CPE assays (*n* = 3). (C) Immunofluorescence assay of VP1 following treatment with VP1 PROTACs or controls at 2 hpi (2–8 hpi) (*n* = 3, mean ± SD, three independent experiments). (D) Cytotoxicity of pleconaril, lenalidomide-5-Br, **Jun15953**, and **Jun15702** in RD cells following 60 h incubation, as measured by neutral red uptake assays. CC_50_ values are reported as mean ± SD from three biological replicates (*n* = 3). (E) Competition experiment between **Jun15702** and lenalidomide-5-Br assessed by immunofluorescence. Intracellular VP1 levels were quantified as the ratio of VP1 to Hoechst nuclear staining using ImageJ. (F) Schematic representation of sample preparation for immunoblot analysis during a single-cycle EV-D68 infection to evaluate VP1 degradation. RD cells were infected with EV-D68 at MOI = 1. Following viral entry, cells were washed at 1 hpi, and compounds were added at 2 hpi and present during the 2–10 hpi period. Cells were harvested at 10 hpi for immunoblotting. Created with BioRender.com. (G) Immunoblot analysis of VP1 degradation induced by **Jun15702** compared with control compounds (pleconaril, lenalidomide-5-Br, and **Jun15953**), as well as cotreatment with **Jun15702** and lenalidomide-5-Br (10 μM). Quantification of normalized VP1 protein abundance is presented as mean ± SD from three independent experiments (*n* = 3). Representative immunoblots are shown, and β-actin was used as a loading control.

Prior to mechanistic studies, the cytotoxicity of pleconaril, lenalidomide-5-Br, **Jun15702**, and **Jun15953** were assessed. None of the compounds exhibited significant cytotoxic effects at concentrations below 50 μM (Figure 4D).

To assess CRBN engagement by **Jun15702**, competition experiments with the E3 ligase ligand lenalidomide-5-Br were performed using the same immunofluorescence assay procedure (Figure 3B). Specifically, RD cells were infected with EV-D68 US/MO/14–18,947 at an MOI of 1, and 10 μM **Jun15702** was added, along with increasing concentrations of lenalidomide-5-Br (10, 30, and 50 μM) at 2 hpi; compounds were present for 2–8 h. Cells were fixed at 8 hpi and quantified by immunofluorescence staining. As shown in Figures 4E and S2B, neither pleconaril (10 μM) nor lenalidomide-5-Br (10 μM) alone induced VP1 depletion, whereas **Jun15702** robustly reduced intracellular VP1 levels. Importantly, increasing concentrations of lenalidomide-5-Br progressively restored VP1 levels, indicating competitive inhibition of **Jun15702**-mediated VP1 degradation by lenalidomide-5-Br and supporting a CRBN-dependent mechanism (Figure 4E).

We further evaluated VP1 degradation by Western blot analysis using a single-cycle viral replication assay (Figure 4F). In this assay, RD cells were infected with EV-D68 US/MO/14–18947 at an MOI of 1 and treated with PROTACs during 2–10 hpi. Consistent with immunofluorescence results, neither pleconaril nor lenalidomide-5-Br induced VP1 degradation, whereas **Jun15702** caused a dose-dependent reduction in VP1 protein levels (Figure 4G). Treatment with **Jun15953** also reduced VP1 levels relative to pleconaril-treated controls but was less effective than **Jun15702**, consistent with the results observed in immunofluorescence experiments (Figure 4C).

Finally, a reciprocal competition experiment was conducted in which a fixed concentration of lenalidomide-5-Br (10 μM) was combined with increasing concentrations of **Jun15702**. VP1 degradation by **Jun15702** was largely preserved until its concentration fell below 3 μM, indicating that **Jun15702** maintains potent CRBN engagement under these conditions. This observation is consistent with immunofluorescence-based competition experiments and further supports a CRBN-dependent degradation mechanism (Figure 4E,G).

### Profiling the Genetic Barrier to Drug Resistance of the VP1 PROTAC **Jun15702**

To test the hypothesis that VP1 PROTAC **Jun15702** has a greater genetic barrier to drug resistance than the inhibitor pleconaril, we performed a serial viral passage experiment to select resistant viruses against **Jun15702** and included pleconaril for side-by-side comparison. In the passage experiment, EV-D68 US/MO/14–18947 virus was amplified in RD cells in the presence of increasing concentrations of **Jun15702** or pleconaril, starting from approximately 1 × EC_50_ of the compound at passage 1 (P1), followed by a 2-fold increase in each subsequent passage (Figure 5A). Viruses from passage 8 in both DMSO-treated (DMSO-P8) and **Jun15702**-treated (**Jun15702**-P8) groups were harvested, and the drug sensitivity of **Jun15702** and pleconaril against the DMSO-P8 and **Jun15702**-P8 viruses was determined in a plaque assay. Despite the high drug pressure of 24 μM at P8, **Jun15702** retained potent antiviral activity against the **Jun15702**-P8 virus with an EC_50_ of 3724 nM, representing a 266-fold decrease in potency compared with the DMSO-P8 virus (EC_50_ = 14.0 nM; Figure 5B). However, pleconaril completely loses antiviral activity against the pleconaril-P8 virus (EC_50_ > 20 μM) compared to the DMSO-P8 virus (EC_50_ = 49.0 nM) (Figure 5B).

**Figure 5:**
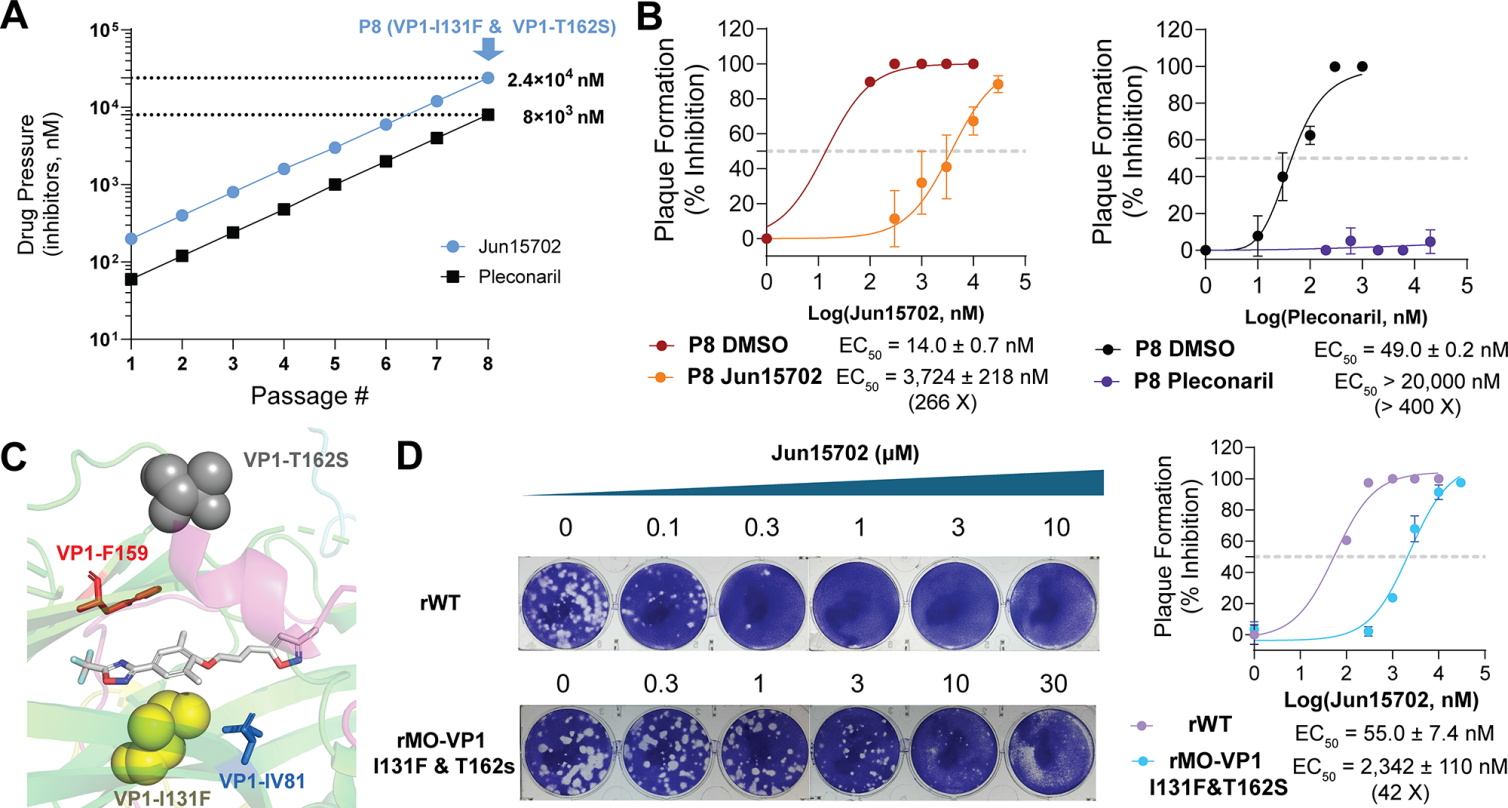
Serial viral passage experiment to profile the genetic barrier to drug resistance of the VP1 PROTAC Jun15702. (A) Schematic illustration of the serial passage of **Jun15702** and pleconaril in RD cells with EV-D68 US/MO/14–18947. (B) Antiviral EC_50_ curves of **Jun15702** and pleconaril against inhibitor-treated and DMSO-treated viruses at P8. EC_50_ values are reported as mean ± SD from two biological replicates in plaque assays (*n* = 2). (C) **Jun15702**-resistance–associated VP1 residues Ile131 and Thr162 are shown as yellow and gray spheres, Pleconaril-resistance-associated VP1 residues Val81 and Phe159 are shown as marine and red spheres, respectively. (D) Plaque assay results of drug sensitivity of **Jun15702** against recombinant wild-type (rWT) and recombinant **Jun15702**-resistant virus (rMO-VP1 I131F & T162S). Representative images from two independent experiments are shown. EC_50_ values are reported as mean ± SD from two biological replicates in plaque assays (*n* = 2).

We subsequently sequenced the whole viral genome of the **Jun15702**-P8 virus to identify resistance-associated mutations. Two substitutions, I131F and T162S, were identified within the viral VP1 protein (Figure 5A). Structural mapping onto the crystal structure of the pleconaril/capsid complex (PDB ID: 4WM7) uncovered that both residues are located within the canyon where pleconaril binds (Figure 5C).

To further validate this resistance phenotype, we generated recombinant EV-D68 viruses containing VP1–I131F and VP1-T162S mutations. The antiviral activity of **Jun15702** was evaluated against the recombinant mutant virus (rMO-VP1 I131F & T162S) and the recombinant wild-type strain (rWT) in a plaque assay (Figure 5D). The EC_50_ of **Jun15702** against rWT was 55.0 nM, whereas the EC_50_ against rMO-VP1 I131F & T162S was 2342 nM, corresponding to approximately 42-fold resistance.

Overall, the serial viral passage experiments validated our hypothesis that the VP1 PROTAC **Jun15702** displays a higher genetic barrier to drug resistance than the inhibitor pleconaril.

## Conclusions

VP1 is a well-validated antiviral drug target for enteroviruses, and several capsid inhibitors have been reported to exhibit potent antiviral activity.^12,33,34^ Pleconaril was originally developed as a rhinovirus antiviral and later repurposed as an inhibitor of the EV-D68 VP1 capsid.^13^ Previous studies by us and others have identified drug-resistant mutations in pleconaril.^17,18^ In this study, we aim to leverage the PROTAC strategy to tackle pleconaril drug resistance. In addition, although antiviral resistance is ultimately inevitable, we aim to investigate whether PROTAC-based degraders can delay the emergence of resistance compared to conventional antiviral inhibitors. To this end, we designed a series of PROTACs by conjugating pleconaril to E3 ligase ligands via diverse linkers. It was found that linker length and hydrophobicity, as well as the E3 ligase ligand, have a profound effect on the antiviral activity and VP1 degradation of PROTACs. Through systematic optimization of linker composition and CRBN-recruiting ligands, we identified **Jun15702** as a first-in-class VP1 degrader with robust antiviral activity across multiple EV-D68 strains. Notably, **Jun15702** maintains submicromolar potency against pleconaril-resistant variants, including the highly resistant VP1–F159 V mutant. Mechanistic studies employing methylated control PROTACs, competition experiments, immunofluorescence imaging, and immunoblot analyses collectively demonstrate that **Jun15702** induces VP1 degradation in a CRBN-dependent manner and acts through a distinct mechanism of action from pleconaril. Furthermore, the serial viral passage experiments to select drug-resistant mutations revealed that the VP1 PROTAC **Jun15702** had a higher genetic barrier to drug resistance than the inhibitor pleconaril. In contrast to pleconaril, which showed complete loss of antiviral activity, **Jun15702** retained potent antiviral activity against the P8 virus. It is worth noting that the high concentration used in the in vitro passage experiments (24 μM) is substantially higher than the antiviral potency of **Jun15702** (EC_50_ = 55.0 nM). This large margin suggests that the selective pressure applied in vitro is considerably greater than what would typically occur under physiological conditions, and therefore, the emergence of resistance in an *in vivo* setting may require a longer period of time. Taken together, these findings demonstrate viral capsid protein degradation as a viable and complementary strategy for antiviral development. This work extends targeted protein degradation beyond host and oncogenic targets to the enteroviral structural protein VP1 and highlights PROTAC-based approaches as a promising strategy for the development of next-generation antivirals against enteroviruses with a higher genetic barrier to drug resistance.

## Supplementary Material



## Notes

Experimental procedures for the antiviral assay, immunofluorescence assay, and Western blots; Figures S1, S2; Synthesis procedures and compound characterization; NMR spectra and HPLC traces (PDF)
