# Supplementary material for: Rational Design of Capsid Protein VP1 Degraders to Overcome Pleconaril Resistance in Inhibiting Enterovirus D68
Source: JACS Au. 2026 Mar 23;6(4):2433–44. doi: 10.1021/jacsau.6c00039 (PMC13126190; doi:10.1021/jacsau.6c00039)
Supplement: Supplementary file 1 [file au6c00039_si_001.pdf]

Supplementary Information

Rational Design of Capsid Protein VP1 Degraders to Overcome Pleconaril Resistance in Inhibiting Enterovirus D68

Kan Li,[1,#] Jiabin Ni,[1,#] Guangjin Fan,[1] and Jun Wang[1,*]

[1]Department of Medicinal Chemistry, Ernest Mario School of Pharmacy, Rutgers, the State University of New Jersey, Piscataway, NJ, 08854, USA

[#]These authors contributed equally to this work

*Corresponding authors. Email: junwang@pharmacy.rutgers.edu (J.W.)

Table of Contents

Experimental Procedures S3

Figure S1. Time-of-addition assay with immunofluorescence staining to quantify intracellular capsid VP1 level of EV-D68 S23

Figure S2. Time-of-addition and competition assays with immunofluorescence staining to quantify intracellular capsid VP1 level of EV-D68 S24

NMR spectra S25

HPLC traces S55

Reference S78

Experimental Procedures

General Information. Solvents and commercially available building blocks were purchased from suppliers and used without purification. All reactions were monitored by thin-layer chromatography (TLC) visualizing under ultraviolet light (254 nm), or liquid chromatography-mass spectrometry (LC-MS). Column chromatography purification was performed by using CombiFlash@ NextGen 300+ system. High-performance liquid chromatography purification was performed by using ACCQPrep HP150 system. The ESI-MS readings were recorded on an Agilent MSD iQ G6160A mass spectrometer. ^{1}H NMR and ^{13}C NMR spectra were recorded on a Bruker AV-400 spectrometer at 400 MHz and 100 MHz, respectively. Coupling constants (J) are expressed in hertz (Hz). NMR data is analyzed with MestReNova (14.1.0). Chemical shifts (δ) of NMR are reported in parts per million (ppm) units. High-resolution mass spectra were recorded on Q-TOF Premier mass spectrometer. The purity of compounds was determined to be over 95% by reverse-phase HPLC analysis. All compounds were characterized by proton and carbon NMR and MS.

5-(3-(2,6-Dimethyl-4-(5-(trifluoromethyl)-1,2,4-oxadiazol-3-yl)phenoxy)propyl) isoxazole-3-carboxylic acid (**1**). The preparation of intermediate 1 was according to the reference (*Eur. J. Med Chem.* 2020, *188*, 112007). ^{1}H NMR (400 MHz, DMSO-d_6) δ 7.77 (s, 2H), 6.49 (s, 1H), 3.89 (t, J = 6.1 Hz, 2H), 3.04 (t, J = 7.6 Hz, 2H), 2.32 (s, 6H), 2.16 (q, J = 7.1 Hz, 2H). MS (ESI) m/z $[M+H]^+$ calcd for $C_{18}H_{17}F_3N_3O_5$: 412.1, found: 412.1.

General Procedure 1 (GP-1). A mixture of **1** (1.0 equiv.), amine (1.1 equiv.), HATU (1.5 equiv.) and DIPEA (2 equiv.) were dissolved in 10 mL DMSO. After that, the mixture was poured into water (10 mL) and extracted by EtOAc (3 × 10 mL). The organic phase was collected, dried over anhydrous $MgSO_4$ and evaporated under reduced pressure. The residue was purified by column chromatography using EtOAc/Hexane as an eluent to give corresponding products **2a-g**.

Tert-butyl-(2-(5-(3-(2,6-dimethyl-4-(5-(trifluoromethyl)-1,2,4-oxadiazol-3-yl)phenoxy)propyl)isoxazole-3-carboxamido)ethyl)carbamate (**2a**). The title compound was prepared according to the GP-1 from 1 and *N*-Boc-ethylenediamine, as a white solid in 65% yield. ^{1}H NMR (400 MHz, $CDCl_3$) δ 7.78 (s, 2H), 7.25 (s, 1H), 6.52 (s, 1H), 4.88

(s, 1H), 3.88 (t, *J* = 6.0 Hz, 2H), 3.59 – 3.50 (m, 2H), 3.41 – 3.31 (m, 2H), 3.12 (t, *J* = 7.6 Hz, 2H), 2.33 (s, 6H), 2.24 (q, *J* = 7.0 Hz, 2H), 1.44 (s, 9H). MS (ESI) m/z $[M+H]^+$ calcd for $C_{25}H_{31}F_3N_5O_6$: 554.2, found: 554.2.

Tert-butyl-(8-(5-(3-(2,6-dimethyl-4-(5-(trifluoromethyl)-1,2,4-oxadiazol-3-yl)phenoxy)propyl)isoxazole-3-carboxamido)octyl)carbamate (**2b**). The title compound was prepared according to the GP-1 from 1 and tert-butyl (8-aminooctyl)carbamate, as a colorless oil in 56% yield. ^{1}H NMR (400 MHz, $CDCl_3$) δ 7.78 (s, 2H), 7.25 (s, 1H), 6.52 (s, 1H), 4.88 (s, 1H), 3.88 (t, *J* = 6.0 Hz, 2H), 3.59 – 3.50 (m, 2H), 3.41 – 3.31 (m, 2H), 3.12 (t, *J* = 7.6 Hz, 2H), 2.33 (s, 6H), 2.24 (q, *J* = 7.0 Hz, 2H), 1.71-1.63 (m, 2H), 1.62-1.53 (m, 2H), 1.44 (s, 9H), 1.43-1.38 (m, 2H), 1.38-1.29 (m, 6H). MS (ESI) m/z $[M+H]^+$ calcd for $C_{31}H_{43}F_3N_5O_6$: 638.3, found: 638.2.

Tert-butyl-(2-(2-(5-(3-(2,6-dimethyl-4-(5-(trifluoromethyl)-1,2,4-oxadiazol-3-yl)phenoxy)propyl)isoxazole-3-carboxamido)ethoxy)ethyl)carbamate (**2c**). The title compound was prepared according to the GP-1 from 1 and Amino-PEG2-NH-Boc, as a yellowish oil in 72% yield. ^{1}H NMR (400 MHz, $CDCl_3$) δ 7.79 (s, 2H), 7.11 (s, 1H), 6.53 (s, 1H), 4.89 (s, 1H), 3.89 (t, *J* = 6.0 Hz, 2H), 3.68 – 3.59 (m, 4H), 3.54 (t, *J* = 5.2 Hz, 2H), 3.33 (d, *J* = 4.9 Hz, 2H), 3.12 (t, *J* = 7.6 Hz, 2H), 2.34 (s, 6H), 2.25 (p, *J* = 6.5 Hz, 2H), 1.45 (s, 9H). MS (ESI) m/z $[M+H]^+$ calcd for $C_{27}H_{35}F_3N_5O_7$: 598.2, found: 598.3.

Tert-butyl-(2-(2-(2-(5-(3-(2,6-dimethyl-4-(5-(trifluoromethyl)-1,2,4-oxadiazol-3-yl)phenoxy)propyl)isoxazole-3-carboxamido)ethoxy)ethoxy)ethyl)carbamate (**2d**). The title compound was prepared according to the GP-1 from 1 and boc-1-amino-3,6-dioxa-8-octanediamine, as a yellowish oil in 75% yield. ^{1}H NMR (400 MHz, $CDCl_3$) δ 7.78 (s, 2H), 7.32 (s, 1H), 6.54 (s, 1H), 5.14 (s, 1H), 3.89 (t, *J* = 6.0 Hz, 2H), 3.68 – 3.63 (m, 8H), 3.57 (t, *J* = 5.2 Hz, 2H), 3.33 (t, *J* = 5.2 Hz, 2H), 3.12 (t, *J* = 7.6 Hz, 2H), 2.34 (s, 6H), 2.28 – 2.21 (m, 2H), 1.45 (s, 9H). MS (ESI) m/z $[M+H]^+$ calcd for $C_{29}H_{39}F_3N_5O_8$: 642.3, found: 642.3.

Tert-butyl-(1-(5-(3-(2,6-dimethyl-4-(5-(trifluoromethyl)-1,2,4-oxadiazol-3-yl)phenoxy)propyl)isoxazol-3-yl)-1-oxo-6,9,12-trioxa-2-azapentadecan-15-yl)carbamate (**2e**). The title compound was prepared according to the GP-1 from 1 and N-Boc-C1-PEG3-C3-NH2, as a white solid in 54% yield. ^{1}H NMR (400 MHz, $CDCl_3$) δ 7.78 (s, 2H),

7.44 (s, 1H), 6.52 (s, 1H), 5.04 (s, 1H), 3.89 (t, *J* = 6.0 Hz, 2H), 3.71 – 3.54 (m, 14H), 3.23 (q, *J* = 7.3 Hz, 2H), 3.11 (t, *J* = 7.6 Hz, 2H), 2.34 (s, 6H), 2.25 (p, *J* = 7.4 Hz, 2H), 1.93 – 1.87 (m, 2H), 1.78 – 1.73 (m, 2H), 1.43 (s, 9H). MS (ESI) m/z $[M+H]^+$ calcd for $C_{33}H_{47}F_3N_5O_9$: 714.3, found: 714.3.

Tert-butyl-(1-(5-(3-(2,6-dimethyl-4-(5-(trifluoromethyl)-1,2,4-oxadiazol-3-yl)phenoxy)propyl)isoxazol-3-yl)-1-oxo-5,8,11,14-tetraoxa-2-azahexadecan-16-yl)carbamate (**2f**). The title compound was prepared according to the GP-1 from 1 and Boc-NH-PEG4-CH2CH2NH2, as a white solid in 37% yield. ^1H NMR (400 MHz, $CDCl_3$) *δ* 7.78 (s, 2H), 7.27 (s, 1H), 6.53 (s, 1H), 5.04 (s, 1H), 3.89 (t, *J* = 6.0 Hz, 2H), 3.71 – 3.60 (m, 16H), 3.55 (t, *J* = 5.1 Hz, 2H), 3.32 (q, *J* = 5.7 Hz, 2H), 3.12 (t, *J* = 7.6 Hz, 2H), 2.34 (s, 6H), 2.24 (p, *J* = 6.6 Hz, 2H), 1.44 (s, 9H). MS (ESI) m/z $[M+H]^+$ calcd for $C_{33}H_{47}F_3N_3O_{10}$: 730.3, found: 730.

Tert-butyl-(1-(5-(3-(2,6-dimethyl-4-(5-(trifluoromethyl)-1,2,4-oxadiazol-3-yl)phenoxy)propyl)isoxazol-3-yl)-1-oxo-5,8,11,14,17-pentaoxa-2-azanonadecan-19-yl)carbamate (**2g**). The title compound was prepared according to the GP-1 from 1 and NH2-PEG5-C2-NH-Boc, as a white solid in 66% yield. ^1H NMR (400 MHz, $CDCl_3$) *δ* 7.78 (s, 2H), 7.25 (s, 1H), 6.52 (s, 1H), 5.05 (s, 1H), 3.89 (t, *J* = 6.0 Hz, 2H), 3.68 – 3.62 (m, 20H), 3.54 (t, *J* = 5.1 Hz, 2H), 3.31 (q, *J* = 5.4 Hz, 2H), 3.12 (t, *J* = 7.6 Hz, 2H), 2.34 (s, 6H), 2.27 – 2.21 (m, 2H), 1.44 (s, 9H). MS (ESI) m/z $[M+H]^+$ calcd for $C_{35}H_{51}F_3N_5O_{11}$: 774.4, found: 774.4.

General Procedure 2 (GP-2). 4-Fluoro-thalidomide (1 mmol, 1 equiv.), DIPEA (5 equiv.) and the corresponding mono-protected diamine (1 equiv.) were dissolved in DMSO. The mixture was stirred at 130 ℃ for 1 h under microwave. After cooling to room temperature, the mixture was diluted with water (10 mL) and then extracted by EtOAc (3 × 15 mL). The combined organic phase was further washed by brine, dried over anhydrous sodium sulfate and evaporated under reduced pressure. The residue was purified by column chromatography to give corresponding products **3a-b**.

Tert-butyl 4-(2-(2,6-dioxopiperidin-3-yl)-1,3-dioxoisoindolin-4-yl)piperazine-1-carboxylate (**3a**). The title compound was prepared according to the GP-2 from 4-fluoro-thalidomide and *N*-Boc-piperazine, as a yellow solid in 73% yield. ^1H NMR (400 MHz, DMSO-d_6) *δ*

11.10 (s, 1H), 7.73 (dd, *J* = 8.3, 7.2 Hz, 1H), 7.38 (dd, *J* = 15.2, 7.7 Hz, 2H), 5.11 (dd, *J* = 12.8, 5.4 Hz, 1H), 3.52 (s, 4H), 3.26 (d, *J*= 4.3 Hz, 4H), 2.95 - 2.81 (m, 1H), 2.66 - 2.52 (m, 2H), 2.08 - 1.99 (m, 1H), 1.43 (s, 9H). The NMR data matched those reported in the literature (*J. Med. Chem.,* 2022, *65*, 9096). MS (ESI) m/z $[M+H]^+$ calcd for $C_{22}H_{27}N_4O_6$: 443.2, found: 443.2.

Tert-butyl (6-((2-(2,6-dioxopiperidin-3-yl)-1,3-dioxoisoindolin-4-yl)amino)hexyl)carbamate (**3b**). The title compound was prepared according to the GP-2 from 4-fluoro-thalidomide and *N*-Boc-1,6-diaminohexane, as a yellow solid in 68% yield. ^{1}H NMR (400 MHz, $CDCl_3$) δ 8.80 (s, 1H), 7.48 (dd, *J* = 8.5, 7.1 Hz, 1H), 7.07 (d, *J* = 7.1 Hz, 1H), 6.87 (d, *J* = 8.6 Hz, 1H), 6.24 (t, *J* = 5.6 Hz, 1H), 4.98 – 4.90 (m, 1H), 4.69 (t, *J* = 6.0 Hz, 1H), 3.25 (q, *J* = 6.6 Hz, 2H), 3.13 – 3.07 (m, 2H), 2.89 – 2.73 (m, 3H), 2.15 – 2.09 (m, 1H), 1.66 (p, *J* = 7.1 Hz, 2H), 1.52 – 1.33 (m, 15H). The NMR data matched those reported in the literature (*J. Med. Chem.* 2018, *61*, 462). MS (ESI) m/z $[M+H]^+$ calcd for $C_{24}H_{33}N_4O_6$: 473.2, found: 473.3.

General Procedure 3 (GP-3). CuI (20 mol%) and $Pd(Ph_3P)_2Cl_2$ (10 mol%) were added to a solution of alkyne (1 equiv.) and ArBr (1 equiv.) in Et_3N (0.4 M) and DMF (0.4 M). The mixture was stirred at 80 °C under an N_2 atmosphere overnight. The reaction mixture was poured into a saturated aqueous solution of NH_4Cl and after separation of the organic layer the aqueous layer was extracted with EtOAc. The combined organic layers were washed with brine, dried over Na_2SO_4, and concentrated in vacuo. The residue was purified by column chromatography to give corresponding products **3c-e**.

Tert-butyl (5-(2-(2,6-dioxopiperidin-3-yl)-1,3-dioxoisoindolin-4-yl)pent-4-yn-1-yl)carbamate (**3c**). The title compound was prepared according to the GP-3 from 4-bromo-2-(2,6-dioxopiperidin-3-yl)isoindoline-1,3-dione and *N*-Boc-4-pentyne-1-amine, as a yellow solid in 84% yield. ^{1}H NMR (400 MHz, $CDCl_3$) δ 8.13 (s, 1H), 7.79 (d, *J* = 7.0 Hz, 1H), 7.74 – 7.63 (m, 2H), 7.448 (s, 1H), 5.00 – 4.93 (m, 1H), 3.36 (q, *J* = 6.6 Hz, 2H), 2.97 – 2.71 (m, 3H), 2.58 (t, *J* = 6.8 Hz, 2H), 2.20 – 2.10 (m, 1H), 1.90 – 1.81 (m, 2H), 1.42 (s, 9H). MS (ESI) m/z $[M+H]^+$ calcd for $C_{23}H_{26}N_3O_6$: 440.2, found: 440.3.

Tert-butyl 5-(2-(2,6-dioxopiperidin-3-yl)-1-oxoisoindolin-5-yl)pent-4-ynoate (**3d**). The title compound was prepared according to the GP-3 from 3-(5-bromo-1-oxoisoindolin-2-

yl)piperidine-2,6-dione and *tert*-butyl pent-4-ynoate, as a yellow solid in 77% yield. ^{1}H NMR (400 MHz, $CDCl_3$) δ 8.16 (s, 1H), 7.80 (d, J = 7.8 Hz, 1H), 7.51 – 7.45 (m, 2H), 5.21 (dd, J = 13.3, 5.1 Hz, 1H), 4.47 (d, J = 16.0 Hz, 1H), 4.30 (d, J = 16.0 Hz, 1H), 2.96 – 2.77 (m, 2H), 2.71 (t, J = 7.4 Hz, 2H), 2.55 (t, J = 7.3 Hz, 2H), 2.40 – 2.28 (m, 1H), 2.24 – 2.16 (m, 1H), 1.47 (s, 9H). MS (ESI) m/z $[M+H]^+$ calcd for $C_{22}H_{25}N_2O_5$: 397.2, found: 397.3.

Tert-butyl 5-(2-(1-methyl-2,6-dioxopiperidin-3-yl)-1-oxoisoindolin-5-yl)pent-4-ynoate (**3e**). The title compound was prepared according to the GP-3 from 3-(5-bromo-1-oxoisoindolin-2-yl)-1-methylpiperidine-2,6-dione and *tert*-butyl pent-4-ynoate, as a yellow solid in 90% yield. ^{1}H NMR (400 MHz, $CDCl_3$) δ 7.80 (d, J = 7.9 Hz, 1H), 7.70 – 7.63 (m, 1H), 7.50 – 7.44 (m, 2H), 5.18 (dd, J = 13.5, 5.1 Hz, 1H), 4.44 (d, J = 16.0 Hz, 1H), 4.30 (d, J = 16.1 Hz, 1H), 3.19 (s, 3H), 2.96 – 2.77 (m, 2H), 2.71 (t, J = 7.4 Hz, 2H), 2.55 (t, J = 7.3 Hz, 2H), 2.38 – 2.25 (m, 1H), 2.23 – 2.16 (m, 1H), 1.47 (s, 9H). MS (ESI) m/z $[M+H]^+$ calcd for $C_{23}H_{27}N_2O_5$: 411.2, found: 411.2.

General procedure for Boc deprotection. The corresponding intermediates **4a-g** or **5a-d** (1 mmol) was dissolved in DCM (5 mL) and then trifluoroacetic acid (1 mL) was added dropwise at 0 ℃. The mixture was then stirred at rt for 2 h. Then the solvent was removed. The orange oily residue was further dried under vacuum.

General Procedure for amide coupling (GP-4). A mixture of 1 (0.1 mmol, 1.0 equiv.), deprotected amine (0.1 mmol, 1.1 equiv.), HATU (1.5 equiv.) and DIPEA (2 equiv.) were dissolved in 2 mL DMSO. After that, the mixture was poured into water (10 mL) and extracted by EtOAc (3 × 10 mL). The organic phase was collected, dried over anhydrous $MgSO_4$ and evaporated under reduced pressure. The residue was purified by reserved phase preparative HPLC (Water/ACN with 0.1% TFA) to obtain corresponding final products.

General Procedure for nucleophilic aromatic substitution (GP-5). Fluorinated CRBN ligands (0.1 mmol, 1 equiv.), DIPEA (0.5 mmol, 5 equiv.) and the intermediate amine (0.1 mmol, 1 equiv.) were dissolved in DMSO. The mixture was stirred at 130 ^oC for 1 h under microwave. After cooling to room temperature, the mixture was diluted with water (10 mL) and extracted by EtOAc (3×10 mL). The organic phase was collected, dried over anhydrous $MgSO_4$ and evaporated under reduced pressure. The residue was purified by

reserved phase preparative HPLC (Water/ACN with 0.1% TFA) to obtain corresponding final products.

5-(3-(2,6-Dimethyl-4-(5-(trifluoromethyl)-1,2,4-oxadiazol-3-yl)phenoxy)propyl)-N-(5-(2-(2,6-dioxopiperidin-3-yl)-1,3-dioxoisoindolin-4-yl)pent-4-yn-1-yl)isoxazole-3-carboxamide (**Jun14956**). The title compound was prepared according to the GP-4 from 1 and 5c, as a white solid in 48% yield. ^{1}H NMR (400 MHz, $CDCl_3$) δ 8.52 (brs, 1H), 7.80 – 7.7 (m, 3H), 7.70 – 7.62 (m, 2H), 7.42 (t, J = 6.2 Hz, 1H), 6.51 (s, 1H), 5.02 (dd, J = 12.2, 5.2 Hz, 1H), 3.87 (t, J = 6.0 Hz, 2H), 3.73 – 3.64 (m, 2H), 3.08 (t, J = 7.7 Hz, 2H), 2.93 – 2.71 (m, 3H), 2.63 (t, J = 6.6 Hz, 2H), 2.32 (s, 6H), 2.25 – 2.19 (m, 2H), 2.19 – 2.12 (m, 1H), 2.01 (d, J = 6.6 Hz, 2H). ^{13}C NMR (101 MHz, $CDCl_3$) δ 174.24, 171.02, 168.82, 168.04, 166.40, 166.31, 165.53 (q, J = 44.4 Hz), 159.29, 159.02, 158.74, 137.99, 133.84, 132.13, 131.99, 130.94, 128.43, 122.61, 121.26, 120.25, 115.97 (q, J = 274.9 Hz), 100.91, 98.07, 70.46, 49.30, 38.58, 31.33, 28.13, 27.47, 23.46, 22.50, 17.40, 16.30. HRMS (ESI) m/z $[M+H]^+$ calcd for $C_{36}H_{32}F_3N_6O_8$: 733.2234, found: 733.2236.

4-(4-(5-(3-(2,6-Dimethyl-4-(5-(trifluoromethyl)-1,2,4-oxadiazol-3-yl)phenoxy)propyl)isoxazole-3-carbonyl)piperazin-1-yl)-2-(2,6-dioxopiperidin-3-yl)isoindoline-1,3-dione (**Jun1522**). The title compound was prepared according to the GP-4 from 1 and 5a, as a yellow solid in 50% yield. ^{1}H NMR (400 MHz, $CDCl_3$) δ 8.43 (brs, 1H), 7.79 (s, 2H), 7.64 (t, J = 7.8 Hz, 1H), 7.46 (d, J = 7.2 Hz, 1H), 7.18 (d, J = 8.4 Hz, 1H), 6.44 (s, 1H), 4.98 (dd, J = 12.1, 5.3 Hz, 1H), 4.16 – 4.08 (m, 2H), 4.06 – 3.98 (m, 2H), 3.91 (t, J = 6.0 Hz, 2H), 3.46 – 3.34 (m, 4H), 3.13 (t, J = 7.7 Hz, 2H), 2.93 – 2.71 (m, 3H), 2.34 (s, 6H), 2.27 (p, J = 6.6 Hz, 2H), 2.16 – 2.10 (m, 1H). ^{13}C NMR (101 MHz, $CDCl_3$) δ 173.24, 170.95, 168.83, 168.17, 167.10, 166.56, 165.54 (q, J = 44.3 Hz), 159.69, 159.05, 158.52, 149.74, 135.81, 134.12, 133.04, 131.99, 128.90, 128.45, 123.35, 120.26, 118.19, 116.50, 115.97 (q, J = 274.8 Hz), 107.08, 102.53, 70.50, 51.69, 50.71, 49.19, 46.99, 42.55, 31.35, 28.17, 23.37, 22.61, 16.32. HRMS (ESI) m/z $[M+H]^+$ calcd for $C_{35}H_{33}F_3N_7O_8$: 736.2343, found: 736.2347.

5-(3-(2,6-Dimethyl-4-(5-(trifluoromethyl)-1,2,4-oxadiazol-3-yl)phenoxy)propyl)-N-(6-((2-(2,6-dioxopiperidin-3-yl)-1,3-dioxoisoindolin-4-yl)amino)hexyl)isoxazole-3-carboxamide (**Jun1554**). The title compound was prepared according to the GP-4 from 1 and 5b, as a

yellow solid in 63% yield. ^{1}H NMR (400 MHz, $CDCl_3$) δ 8.56 (brs, 1H), 7.78 (s, 2H), 7.48 (t, J = 7.6 Hz, 1H), 7.08 (d, J = 7.1 Hz, 1H), 6.96 (t, J = 6.0 Hz, 1H), 6.87 (d, J = 8.5 Hz, 1H), 6.54 (s, 1H), 6.24 (t, J = 5.6 Hz, 1H), 4.94 (dd, J = 11.8, 5.3 Hz, 1H), 3.89 (t, J = 6.0 Hz, 2H), 3.44 (q, J = 6.8 Hz, 2H), 3.26 (q, J = 6.6 Hz, 2H), 3.15 – 3.06 (m, 2H), 2.92 – 2.68 (m, 3H), 2.33 (s, 6H), 2.25 (p, J = 6.3 Hz, 2H), 2.16 – 2.10 (m, 1H), 1.72 – 1.61 (m, 4H), 1.50 – 1.39 (m, 4H). ^{13}C NMR (101 MHz, $CDCl_3$) δ 174.36, 171.22, 169.50, 168.82, 168.51, 167.57, 165.51 (q, J = 44.6 Hz), 159.02, 158.97, 158.75, 146.91, 136.04, 132.46, 131.99, 128.42, 120.24, 116.58, 115.96 (q, J = 274.9 Hz), 111.33, 109.88, 100.95, 70.44, 48.84, 42.47, 39.27, 31.35, 29.25, 28.97, 28.15, 26.49, 26.46, 23.48, 22.74, 16.29. HRMS (ESI) m/z $[M+H]^+$ calcd for $C_{37}H_{39}F_3N_7O_8$: 766.2812, found: 766.2813.

5-(3-(2,6-Dimethyl-4-(5-(trifluoromethyl)-1,2,4-oxadiazol-3-yl)phenoxy)propyl)-N-(2-(2-(2-((2-(2,6-dioxopiperidin-3-yl)-1,3-dioxoisoindolin-4-yl)amino)ethoxy)ethoxy)ethyl)isoxazole-3-carboxamide (**Jun15183**). The title compound was prepared according to the GP-5 from 4d and 2-(2,6-dioxopiperidin-3-yl)-4-fluoroisoindoline-1,3-dione, as a green solid in 44% yield. ^{1}H NMR (400 MHz, $CDCl_3$) δ 8.48 (brs, 1H), 7.78 (s, 2H), 7.48 (t, J = 7.8 Hz, 1H), 7.38 (d, J = 5.3 Hz, 1H), 7.10 (d, J = 7.1 Hz, 1H), 6.90 (d, J = 8.5 Hz, 1H), 6.55 (t, J = 5.5 Hz, 1H), 6.51 (s, 1H), 4.96 (dd, J = 11.9, 5.4 Hz, 1H), 3.88 (t, J = 6.0 Hz, 2H), 3.75 (t, J = 5.2 Hz, 2H), 3.71 – 3.60 (m, 8H), 3.50 – 3.44 (m, 2H), 3.08 (t, J = 7.6 Hz, 2H), 2.89 – 2.70 (m, 3H), 2.33 (s, 6H), 2.27 – 2.20 (m, 2H), 2.16 – 2.10 (m, 1H). ^{13}C NMR (101 MHz, $CDCl_3$) δ 174.32, 171.11, 169.38, 168.55, 165.53 (q, J = 44.4 Hz), 167.61, 159.18, 159.09, 158.79, 146.81, 136.01, 132.58, 132.06, 128.50, 120.32, 116.73, 115.96 (q, J = 274.9 Hz), 111.63, 110.41, 101.05, 70.73, 70.52, 70.33, 69.58, 69.30, 48.91, 42.33, 39.37, 31.40, 28.20, 23.54, 22.86, 16.37. HRMS (ESI) m/z $[M+H]^+$ calcd for $C_{37}H_{39}F_3N_7O_{10}$: 798.2711, found: 798.2721.

5-(3-(2,6-Dimethyl-4-(5-(trifluoromethyl)-1,2,4-oxadiazol-3-yl)phenoxy)propyl)-N-(8-((2-(2,6-dioxopiperidin-3-yl)-1,3-dioxoisoindolin-4-yl)amino)octyl)isoxazole-3-carboxamide (**Jun15191**). The title compound was prepared according to the GP-5 from 4b and 2-(2,6-dioxopiperidin-3-yl)-4-fluoroisoindoline-1,3-dione, as a green solid in 77% yield. ^{1}H NMR (400 MHz, $CDCl_3$) δ 8.13 (brs, 1H), 7.78 (s, 2H), 7.52 – 7.47 (m, 1H), 7.09 (d, J = 7.1 Hz, 1H), 6.88 (d, J = 8.4 Hz, 2H), 6.54 (s, 1H), 4.93 (dd, J = 12.0, 5.3 Hz, 1H), 3.89 (t, J = 6.0

Hz, 2H), 3.43 (q, J = 6.8 Hz, 2H), 3.27 (t, J = 6.9 Hz, 2H), 3.12 (t, J = 7.6 Hz, 2H), 2.93 – 2.69 (m, 4H), 2.33 (s, 6H), 2.28 – 2.21 (m, 2H), 2.18 – 2.11 (m, 1H), 1.70 – 1.56 (m, 4H), 1.46 – 1.30 (m, 8H). ^{13}C NMR (101 MHz, $CDCl_3$) δ 174.47, 170.95, 169.51, 168.89, 168.37, 167.62, 165.52 (q, J = 44.3 Hz), 159.08, 158.75, 147.04, 136.11, 132.51, 132.05, 128.50, 120.32, 116.66, 115.97 (q, J = 274.9 Hz), 111.38, 109.88, 101.00, 70.48, 48.87, 42.57, 39.54, 31.40, 29.30, 29.03, 29.00, 28.21, 26.73, 26.66, 23.55, 22.82, 16.35. HRMS (ESI) m/z $[M+H]^+$ calcd for $C_{39}H_{43}F_3N_7O_8$: 794.3125, found: 794.3129.

5-(3-(2,6-Dimethyl-4-(5-(trifluoromethyl)-1,2,4-oxadiazol-3-yl)phenoxy)propyl)-N-(2-(2-((2-(2,6-dioxopiperidin-3-yl)-1,3-dioxoisoindolin-5-yl)amino)ethoxy)ethyl) isoxazole-3-carboxamide (**Jun15373**). The title compound was prepared according to the GP-5 from 4c and 2-(2,6-dioxopiperidin-3-yl)-5-fluoroisoindoline-1,3-dione, as a green solid in 65% yield. ^{1}H NMR (400 MHz, $CDCl_3$) δ 8.59 (brs, 1H), 7.77 (s, 2H), 7.55 (t, J = 7.7 Hz, 1H), 7.26 – 7.18 (m, 1H), 6.94 (s, 1H), 6.79 – 6.70 (m, 1H), 6.55 (s, 1H), 5.27 (s, 1H), 4.94 (dd, J = 11.9, 5.6 Hz, 1H), 3.91 – 3.87 (m, 2H), 3.83 – 3.52 (m, 8H), 3.42 – 3.31 (m, 2H), 3.13 (t, J = 7.6 Hz, 2H), 2.92 – 2.66 (m, sH), 2.32 (s, 6H), 2.27 – 2.22 (d, J = 7.1 Hz, 2H), 2.14 – 2.07 (m, 1H). ^{13}C NMR (101 MHz, $CDCl_3$) δ 174.71, 174.17, 168.84, 168.64, 167.85, 167.37, 165.52 (q, J = 44.3 Hz), 159.38, 159.04, 158.57, 153.60, 134.51, 132.02, 128.46, 125.39, 120.28, 118.44, 116.44, 115.99 (q, J = 274.8 Hz), 106.29, 100.93, 70.48, 69.52, 68.69, 49.02, 42.96, 38.98, 31.41, 28.15, 23.55, 22.73, 16.32. HRMS (ESI) m/z $[M+H]^+$ calcd for $C_{35}H_{35}F_3N_7O_9$: 754.2448, found: 754.2430.

5-(3-(2,6-Dimethyl-4-(5-(trifluoromethyl)-1,2,4-oxadiazol-3-yl)phenoxy)propyl)-N-(2-(2-(2-((2-(2,6-dioxopiperidin-3-yl)-1,3-dioxoisoindolin-5-yl)amino)ethoxy)ethoxy)ethyl)isoxazole-3-carboxamide (**Jun15192**). The title compound was prepared according to the GP-5 from 4d and 2-(2,6-dioxopiperidin-3-yl)-5-fluoroisoindoline-1,3-dione, as a green solid in 72% yield. ^{1}H NMR (400 MHz, $CDCl_3$) δ 8.04 (brs, 1H), 7.78 (s, 2H), 7.59 (d, J = 8.3 Hz, 1H), 7.00 (d, J = 2.2 Hz, 1H), 6.79 (dd, J = 8.3, 2.2 Hz, 1H), 6.54 (s, 1H), 5.23 (brs, 1H), 4.92 (dd, J = 12.1, 5.3 Hz, 1H), 3.88 (t, J = 6.0 Hz, 2H), 3.75 (t, J = 5.1 Hz, 2H), 3.69 – 3.64 (m, 8H), 3.44 (t, J = 5.2 Hz, 2H), 3.11 (t, J = 7.7 Hz, 2H), 2.91 – 2.67 (m, 3H), 2.33 (s, 6H), 2.24 (p, J = 7.1 Hz, 2H), 2.15 – 2.08 (m, 1H). ^{13}C NMR (101 MHz, $CDCl_3$) δ 174.58, 170.90, 168.88, 168.30, 167.88, 167.36,

165.52 (q, *J* = 44.3 Hz), 159.07, 159.03, 158.73, 153.70, 134.63, 132.04, 128.49, 125.43, 120.32, 118.56, 116.74, 115.98 (q, *J* = 274.7 Hz), 106.28, 101.05, 70.49, 70.44, 70.30, 69.44, 69.11, 49.07, 43.13, 39.15, 31.43, 28.20, 23.56, 22.77, 16.35. HRMS (ESI) m/z $[M+H]^+$ calcd for $C_{37}H_{39}F_3N_7O_{10}$: 798.2711, found: 798.2719.

5-(3-(2,6-Dimethyl-4-(5-(trifluoromethyl)-1,2,4-oxadiazol-3-yl)phenoxy)propyl)-N-(3-(2-(2-(3-((2-(2,6-dioxopiperidin-3-yl)-1,3-dioxoisoindolin-5-yl)amino)propoxy)ethoxy)ethoxy)propyl)isoxazole-3-carboxamide (**Jun15294**). The title compound was prepared according to the GP-5 from 4e and 2-(2,6-dioxopiperidin-3-yl)-5-fluoroisoindoline-1,3-dione, as a green solid in 81% yield. ^1H NMR (400 MHz, $CDCl_3$) δ 8.17 (brs, 1H), 7.78 (s, 2H), 7.60 (d, *J* = 8.3 Hz, 1H), 7.42 (d, *J* = 6.1 Hz, 1H), 6.97 (d, *J* = 2.2 Hz, 1H), 6.73 (dd, *J* = 8.4, 2.2 Hz, 1H), 6.51 (s, 1H), 4.94 (dd, *J* = 12.1, 5.2 Hz, 1H), 3.88 (t, *J* = 6.0 Hz, 2H), 3.73 – 3.69 (m, 3H), 3.67 – 3.49 (m, 10H), 3.38 – 3.33 (m, 2H), 3.10 (t, *J* = 7.6 Hz, 2H), 2.92 – 2.69 (m, 3H), 2.33 (s, 6H), 2.23 (p, *J* = 6.8 Hz, 2H), 2.15 – 2.08 (m, 1H), 1.97 – 1.83 (m, 4H). ^{13}C NMR (101 MHz, $CDCl_3$) δ 174.24, 170.96, 168.48, 168.00, 167.46, 165.53 (q, *J* = 44.4 Hz), 159.08, 158.98, 158.89, 154.05, 134.59, 132.05, 128.50, 125.56, 120.32, 117.78, 115.66, 106.61, 100.99, 70.59, 70.51, 70.40, 70.25, 70.04, 69.83, 49.05, 42.26, 37.77, 31.45, 28.95, 28.22, 23.54, 22.79, 16.36. HRMS (ESI) m/z $[M+H]^+$ calcd for $C_{41}H_{47}F_3N_7O_{11}$: 870.3286, found: 870.3290.

5-(3-(2,6-Dimethyl-4-(5-(trifluoromethyl)-1,2,4-oxadiazol-3-yl)phenoxy)propyl)-N-(14-((2-(2,6-dioxopiperidin-3-yl)-1,3-dioxoisoindolin-5-yl)amino)-3,6,9,12-tetraoxatetradecyl)isoxazole-3-carboxamide (**Jun15374**). The title compound was prepared according to the GP-5 from 4f and 2-(2,6-dioxopiperidin-3-yl)-5-fluoroisoindoline-1,3-dione, as a green solid in 68% yield. ^1H NMR (400 MHz, $CDCl_3$) δ 8.33 (brs, 1H), 7.78 (s, 2H), 7.59 (d, *J* = 8.3 Hz, 1H), 7.32 (t, *J* = 5.0 Hz, 1H), 6.98 (d, *J* = 2.2 Hz, 1H), 6.77 (dd, *J* = 8.4, 2.2 Hz, 1H), 6.52 (s, 1H), 5.36 (t, *J* = 5.3 Hz, 1H), 4.93 (dd, *J* = 12.1, 5.3 Hz, 1H), 3.88 (t, *J* = 6.0 Hz, 2H), 3.74 (t, *J* = 5.0 Hz, 2H), 3.69 – 3.58 (m, 16H), 3.39 (q, *J* = 5.1 Hz, 2H), 3.11 (t, *J* = 7.6 Hz, 2H), 2.92 – 2.65 (m, 3H), 2.33 (s, 6H), 2.24 (p, *J* = 6.6 Hz, 2H), 2.16 – 2.09 (m, 1H). ^{13}C NMR (101 MHz, $CDCl_3$) δ 174.28, 171.08, 168.86, 168.49, 167.91, 167.38, 165.56 (q, *J* = 45.6 Hz), 159.11, 159.06, 158.68, 153.77, 134.57, 132.02, 128.47, 125.44, 120.29, 118.33, 116.38, 106.45, 115.99 (q, *J* =

274.8 Hz), 100.97, 70.64, 70.57, 70.52, 70.48, 70.36, 70.34, 69.47, 68.82, 49.05, 43.10, 39.21, 31.43, 28.19, 23.51, 22.75, 16.33. HRMS (ESI) m/z $[M+H]^+$ calcd for $C_{41}H_{47}F_3N_7O_{12}$: 886.3235, found: 886.3245.

5-(3-(2,6-Dimethyl-4-(5-(trifluoromethyl)-1,2,4-oxadiazol-3-yl)phenoxy)propyl)-N-(17-((2-(2,6-dioxopiperidin-3-yl)-1,3-dioxoisoindolin-5-yl)amino)-3,6,9,12,15-pentaoxaheptadecyl)isoxazole-3-carboxamide (**Jun15412**). The title compound was prepared according to the GP-5 from 4g and 2-(2,6-dioxopiperidin-3-yl)-5-fluoroisoindoline-1,3-dione, as a green solid in 74% yield. ^{1}H NMR (400 MHz, $CDCl_3$) δ 8.48 (brs, 1H), 7.78 (s, 2H), 7.60 (d, J = 8.3 Hz, 1H), 7.52 (s, 1H), 7.00 (d, J = 2.1 Hz, 1H), 6.80 (dd, J = 8.4, 2.2 Hz, 1H), 6.55 (s, 1H), 4.98 – 4.90 (m, 1H), 3.89 (t, J = 6.0 Hz, 2H), 3.75 (t, J = 5.0 Hz, 2H), 3.70 – 3.63 (m, 20H), 3.40 (t, J = 5.0 Hz, 2H), 3.12 (t, J = 7.6 Hz, 2H), 2.95 – 2.65 (m, 3H), 2.33 (s, 6H), 2.27 – 2.21 (m, 2H), 2.18 – 2.10 (m, 1H). ^{13}C NMR (101 MHz, $CDCl_3$) δ 174.68, 171.56, 168.86, 168.72, 167.94, 167.49, 165.58 (q, J = 44.5 Hz), 159.77, 159.04, 158.27, 153.70, 134.52, 132.03, 128.48, 125.54, 120.30, 118.36, 116.67, 106.54, 115.99 (q, J = 274.6 Hz), 100.97, 70.46, 70.44, 70.41, 70.19, 69.25, 68.81, 49.03, 43.09, 39.42, 31.37, 28.17, 23.54, 22.70, 16.31. HRMS (ESI) m/z $[M+H]^+$ calcd for $C_{43}H_{51}F_3N_7O_{13}$: 930.3497, found: 930.3510.

5-(3-(2,6-Dimethyl-4-(5-(trifluoromethyl)-1,2,4-oxadiazol-3-yl)phenoxy)propyl)-N-(2-(2-(2-(2-((2-(2,6-dioxopiperidin-3-yl)-1,3-dioxoisoindolin-4-yl)oxy)ethoxy)ethoxy) acetamido)ethyl)isoxazole-3-carboxamide (**Jun15322**). The title compound was prepared according to the GP-4 from 4a and 2-(2-(2-((2-(2,6-dioxopiperidin-3-yl)-1,3-dioxoisoindolin-4-yl)oxy)ethoxy)ethoxy)acetic acid, as a white solid in 63% yield. ^{1}H NMR (400 MHz, $CDCl_3$) δ 8.89 (s, 1H), 7.92 (t, J = 5.9 Hz, 1H), 7.78 (s, 3H), 7.70 (dd, J = 8.4, 7.3 Hz, 1H), 7.49 (d, J = 7.2 Hz, 1H), 7.24 (d, J = 8.5 Hz, 1H), 6.52 (s, 1H), 5.05 – 4.98 (m, 1H), 4.40 – 4.32 m, 2H), 4.11 (s, 2H), 4.01 – 3.93 (m, 2H), 3.88 (t, J = 5.9 Hz, 2H), 3.83 – 3.78 (m, 2H), 3.75 – 3.70 (m, 2H), 3.61 – 3.56 (m, 2H), 3.55 – 3.49 (q, J = 5.3 Hz, 2H), 3.10 (t, J = 7.7 Hz, 2H), 2.93 – 2.84 (m, 1H), 2.83 – 2.76 (m, 2H), 2.33 (s, 6H), 2.26 – 2.20 (m, 2H), 2.17 – 2.11 (m, 1H). ^{13}C NMR (101 MHz, $CDCl_3$) δ 174.95, 173.30, 171.86, 168.86, 168.82, 166.84, 166.10, 165.58 (q, J = 44.4 Hz), 160.34, 159.02, 158.01, 156.03, 136.81, 133.75, 132.04, 128.48, 120.30, 119.11, 117.21, 116.53, 115.99 (q, J = 274.6

Hz), 100.87, 70.81, 70.60, 70.48, 69.53, 69.22, 68.87, 49.19, 39.41, 38.79, 31.26, 28.10, 23.55, 22.52, 16.30. HRMS (ESI) m/z $[M+H]^+$ calcd for $C_{39}H_{41}F_3N_7O_{12}$: 856.2765, found: 856.2770.

5-(3-(2,6-Dimethyl-4-(5-(trifluoromethyl)-1,2,4-oxadiazol-3-yl)phenoxy)propyl)-N-(14-((2-(2,6-dioxopiperidin-3-yl)-1,3-dioxoisoindolin-4-yl)oxy)-4-oxo-6,9,12-trioxa-3-azatetradecyl)isoxazole-3-carboxamide (**Jun15323**). The title compound was prepared according to the GP-4 from 4a and 2-(2-(2-(2-((2-(2,6-dioxopiperidin-3-yl)-1,3-dioxoisoindolin-4-yl)oxy)ethoxy)ethoxy)ethoxy)acetic acid, as a white solid in 55% yield. ^{1}H NMR (400 MHz, $CDCl_3$) δ 8.88 (brs, 1H), 8.04 (t, J = 5.8 Hz, 1H), 7.83 (t, J = 5.6 Hz, 1H), 7.78 (s, 2H), 7.68 (t, J = 7.9 Hz, 1H), 7.47 (d, J = 7.3 Hz, 1H), 7.24 (d, J = 8.5 Hz, 1H), 6.52 (s, 1H), 5.04 – 4.97 (m, 1H), 4.34 (t, J = 4.3 Hz, 2H), 4.06 (s, 2H), 3.99 – 3.94 (m, 2H), 3.88 (t, J = 6.0 Hz, 2H), 3.85 – 3.81 (m, 2H), 3.72 – 3.69 (m, 2H), 3.68 – 3.65 (m, 4H), 3.61 (t, J = 5.1 Hz, 2H), 3.56 (t, J = 4.9 Hz, 2H), 3.10 (t, J = 7.7 Hz, 2H), 2.93 – 2.85 (m, 1H), 2.84 – 2.75 (m, 2H), 2.33 (s, 7H), 2.26 – 2.20 (m, 2H), 2.18 – 2.12 (m, 1H). ^{13}C NMR (101 MHz, $CDCl_3$) δ 174.87, 173.49, 171.89, 168.85, 168.81, 166.89, 165.92, 165.56 (q, J = 44.4 Hz), 160.35, 159.02, 158.03, 156.14, 136.71, 133.69, 132.02, 128.46, 120.28, 119.06, 117.11, 116.32, 115.98 (q, J = 274.4 Hz), 100.85, 70.96, 70.77, 70.47, 70.24, 69.93, 69.46, 69.18, 69.01, 49.12, 39.52, 38.82, 31.24, 28.09, 23.53, 22.51, 16.28. HRMS (ESI) m/z $[M+H]^+$ calcd for $C_{41}H_{45}F_3N_7O_{13}$: 900.3027, found: 900.3030.

5-(3-(2,6-Dimethyl-4-(5-(trifluoromethyl)-1,2,4-oxadiazol-3-yl)phenoxy)propyl)-N-(17-((2-(2,6-dioxopiperidin-3-yl)-1,3-dioxoisoindolin-4-yl)oxy)-4-oxo-6,9,12,15-tetraoxa-3-azaheptadecyl)isoxazole-3-carboxamide (**Jun15331**). The title compound was prepared according to the GP-4 from 4a and 14-((2-(2,6-dioxopiperidin-3-yl)-1,3-dioxoisoindolin-4-yl)oxy)-3,6,9,12-tetraoxatetradecanoic acid, as a white solid in 60% yield. ^{1}H NMR (400 MHz, $CDCl_3$) δ 8.81 (brs, 1H), 8.04 (t, J = 5.7 Hz, 1H), 7.85 – 7.80 (m, 1H), 7.78 (s, 2H), 7.68 (t, J = 7.9 Hz, 1H), 7.46 (d, J = 7.3 Hz, 1H), 7.23 (d, J = 8.5 Hz, 1H), 6.51 (s, 1H), 5.03 – 4.96 (m, 1H), 4.33 (t, J = 4.4 Hz, 2H), 4.06 (s, 2H), 3.97 – 3.92 (m, 2H), 3.88 (t, J = 5.9 Hz, 2H), 3.82 – 3.78 (m, 2H), 3.69 – 3.58 (m, 14H), 3.10 (t, J = 7.6 Hz, 2H), 2.92 – 2.85 (m, 1H), 2.84 – 2.74 (m, 2H), 2.33 (s, 6H), 2.26 – 2.21 (m, 2H), 2.17 – 2.12 (m, 1H). ^{13}C NMR (101 MHz, $CDCl_3$) δ 174.66, 173.13, 171.61, 168.85, 168.67, 166.91, 165.79,

165.56 (q, J = 44.4 Hz), 160.13, 159.04, 158.64, 158.21, 156.22, 136.61, 133.73, 132.02, 128.46, 120.28, 119.14, 117.16, 116.22, 115.98 (q, J = 274.4 Hz), 100.82, 71.04, 70.94, 70.49, 70.41, 70.31, 70.26, 69.89, 69.53, 69.24, 69.11, 49.11, 39.54, 38.78, 31.28, 28.12, 23.53, 22.56, 16.29. HRMS (ESI) m/z $[M+H]^+$ calcd for $C_{43}H_{49}F_3N_7O_{14}$: 944.3290, found: 944.3298.

5-(3-(2,6-Dimethyl-4-(5-(trifluoromethyl)-1,2,4-oxadiazol-3-yl)phenoxy)propyl)-N-(2-(2-(2-(2-((2-(2,6-dioxopiperidin-3-yl)-1,3-dioxoisoindolin-4-yl)oxy)acetamido)ethoxy)ethoxy)ethyl)isoxazole-3-carboxamide (**Jun15375**). The title compound was prepared according to the GP-4 from 4d and 2-((2-(2,6-dioxopiperidin-3-yl)-1,3-dioxoisoindolin-4-yl)oxy)acetic acid, as a white solid in 46% yield. ^1H NMR (400 MHz, $CDCl_3$) δ 7.78 (s, 2H), 7.68 – 7.63 (m, 1H), 7.50 (d, J = 7.3 Hz, 1H), 7.40 – 7.35 (m, 1H), 7.10 (d, J = 8.4 Hz, 1H), 6.81 (t, J = 5.7 Hz, 1H), 6.54 (s, 1H), 5.11 (dd, J = 12.3, 5.5 Hz, 1H), 4.54 (d, J = 3.2 Hz, 1H), 3.87 (t, J = 6.0 Hz, 2H), 3.80 (s, 2H), 3.68 – 3.61 (m, 8H), 3.56 (t, J = 5.0 Hz, 2H), 3.51 – 3.44 (m, 2H), 3.12 (t, J = 7.6 Hz, 2H), 3.01 – 2.95 (m, 1H), 2.94 – 2.86 (m, 1H), 2.84 – 2.74 (m, 1H), 2.32 (s, 6H), 2.27 – 2.21 (m, 2H), 2.18 – 2.11 (m, 1H). ^{13}C NMR (101 MHz, $CDCl_3$) δ 174.75, 170.77, 168.88, 168.55, 168.33, 167.36, 166.68, 165.58 (q, J = 44.2 Hz), 159.32, 159.09, 158.61, 155.22, 136.39, 133.94, 132.07, 128.47, 120.27, 119.93, 117.75, 117.15, 115.98 (q, J = 274.4 Hz), 101.04, 70.51, 70.28, 70.02, 69.55, 69.15, 66.16, 49.61, 39.65, 39.24, 31.44, 28.15, 23.57, 21.96, 16.33. HRMS (ESI) m/z $[M+H]^+$ calcd for $C_{39}H_{41}F_3N_7O_{12}$: 856.2765, found: 856.2772.

5-(3-(2,6-Dimethyl-4-(5-(trifluoromethyl)-1,2,4-oxadiazol-3-yl)phenoxy)propyl)-N-(17-((2-(2,6-dioxopiperidin-3-yl)-1,3-dioxoisoindolin-4-yl)oxy)-10-oxo-3,6,12,15-tetraoxa-9-azaheptadecyl)isoxazole-3-carboxamide (**Jun15332**). The title compound was prepared according to the GP-4 from 4d and 2-(2-(2-((2-(2,6-dioxopiperidin-3-yl)-1,3-dioxoisoindolin-4-yl)oxy)ethoxy)ethoxy)acetic acid, as a white solid in 50% yield. ^1H NMR (400 MHz, $CDCl_3$) δ 8.83 (brs, 1H), 7.78 (s, 2H), 7.69 (t, J = 7.9 Hz, 1H), 7.61 (d, J = 5.8 Hz, 1H), 7.54 – 7.46 (m, 2H), 7.24 (d, J = 8.5 Hz, 1H), 6.56 (s, 1H), 5.02 – 4.94 (m, 1H), 4.35 (t, J = 4.4 Hz, 2H), 4.14 (s, 2H), 3.96 (t, J = 4.4 Hz, 2H), 3.89 (t, J = 6.0 Hz, 2H), 3.85 – 3.82 (m, 2H), 3.77 – 3.72 (m, 2H), 3.67 – 3.61 (m, 8H), 3.57 (t, J = 5.2 Hz, 2H), 3.51 – 3.45 (m, 2H), 3.11 (t, J = 7.7 Hz, 2H), 2.91 – 2.83 (m, 1H), 2.83 – 2.72 (m, 2H), 2.33 (s,

6H), 2.27 – 2.21 (m, 2H), 2.17 – 2.11 (m, 1H). ^{13}C NMR (101 MHz, $CDCl_3$) δ 174.79, 173.30, 171.53, 168.87, 168.71, 166.84, 165.92, 165.57 (q, J = 44.3 Hz), 159.71, 159.05, 158.31, 156.11, 136.68, 133.74, 132.04, 128.48, 120.29, 119.15, 117.26, 116.39, 115.99 (q, J = 274.5 Hz), 101.01, 70.86, 70.73, 70.49, 70.14, 70.03, 69.69, 69.26, 69.23, 69.12, 49.14, 39.37, 39.06, 31.29, 28.14, 23.55, 22.55, 16.31. HRMS (ESI) m/z $[M+H]^+$ calcd for $C_{43}H_{49}F_3N_7O_{14}$: 944.3290, found: 944.3300.

5-(3-(2,6-Dimethyl-4-(5-(trifluoromethyl)-1,2,4-oxadiazol-3-yl)phenoxy)propyl)-N-(20-((2-(2,6-dioxopiperidin-3-yl)-1,3-dioxoisoindolin-4-yl)oxy)-10-oxo-3,6,12,15,18-pentaoxa-9-azaicosyl)isoxazole-3-carboxamide (**Jun15333**). The title compound was prepared according to the GP-4 from 4d and 2-(2-(2-(2-((2-(2,6-dioxopiperidin-3-yl)-1,3-dioxoisoindolin-4-yl)oxy)ethoxy)ethoxy)ethoxy)acetic acid, as a white solid in 30% yield. ^1H NMR (400 MHz, $CDCl_3$) δ 8.95 (brs, 1H), 7.78 (s, 2H), 7.78 – 7.67 (m, 2H), 7.56 (s, 1H), 7.47 (d, J = 7.3 Hz, 1H), 7.25 (d, J = 8.5 Hz, 1H), 6.56 (s, 1H), 5.02 – 4.94 (m, 1H), 4.35 (t, J = 4.5 Hz, 2H), 4.11 (s, 2H), 3.98 – 3.94 (m, 2H), 3.89 (t, J = 6.0 Hz, 2H), 3.84 – 3.81 (m, 2H), 3.71 – 3.63 (m, 14H), 3.60 (t, J = 5.2 Hz, 2H), 3.52 – 3.47 (m, 2H), 3.12 (t, J = 7.7 Hz, 2H), 2.93 – 2.85 (m, 1H), 2.83 – 2.73 (m, 2H), 2.33 (s, 6H), 2.27 – 2.21 (m, 2H), 2.16 – 2.10 (m, 1H). ^{13}C NMR (101 MHz, $CDCl_3$) δ 174.85, 172.49, 171.86, 168.82, 168.68, 166.87, 165.91, 165.53 (q, J = 44.3 Hz), 159.84, 159.00, 158.18, 156.16, 136.65, 133.62, 132.01, 128.44, 120.25, 119.17, 117.11, 116.26, 100.92, 70.87, 70.44, 70.27, 70.10, 70.01, 69.99, 69.51, 69.12, 69.01, 49.07, 39.39, 39.08, 31.23, 28.10, 23.51, 22.47, 16.25. HRMS (ESI) m/z $[M+H]^+$ calcd for $C_{45}H_{53}F_3N_7O_{15}$: 988.3552, found: 988.3553.

5-(3-(2,6-Dimethyl-4-(5-(trifluoromethyl)-1,2,4-oxadiazol-3-yl)phenoxy)propyl)-N-(3-(2-(2-(3-((2-(2,6-dioxopiperidin-3-yl)-6-fluoro-1,3-dioxoisoindolin-5-yl)amino) propoxy)ethoxy)ethoxy)propyl)isoxazole-3-carboxamide (**Jun15616**). The title compound was prepared according to the GP-5 from 4f and 2-(2,6-dioxopiperidin-3-yl)-5,6-difluoroisoindoline-1,3-dione, as a green solid in 83% yield. ^1H NMR (400 MHz, $CDCl_3$) δ 8.56 (brs, 1H), 7.78 (s, 2H), 7.72 – 7.66 (m, 1H), 7.37 (d, J = 9.8 Hz, 1H), 7.07 (d, J = 7.1 Hz, 1H), 6.55 (s, 1H), 4.94 (dd, J = 12.0, 5.4 Hz, 1H), 3.88 (t, J = 6.0 Hz, 2H), 3.78 – 3.69 (m, 4H), 3.69 – 3.59 (m, 8H), 3.56 (q, J = 6.3 Hz, 2H), 3.40 (t, J = 6.4 Hz, 2H), 3.11 (t, J = 7.6 Hz, 2H), 2.92 – 2.70 (m, 3H), 2.33 (s, 6H), 2.27 – 2.19 (m, 2H), 2.17 – 2.10 (m, 1H),

1.97 (p, *J* = 6.0 Hz, 2H), 1.88 (p, *J* = 6.1 Hz, 2H). ^{13}C NMR (101 MHz, $CDCl_3$) *δ* 174.66, 171.52, 168.84, 168.64, 167.48, 166.94 (d, *J* = 2.90 Hz), 165.55 (q, *J* = 44.32 Hz), 159.64, 159.02, 158.35, 154.90, 152.43, 142.89 (d, *J* = 12.83 Hz), 132.01, 130.10 (d, *J* = 2.17 Hz), 128.45, 120.28, 117.90 (d, *J* = 8.74 Hz), 115.98 (q, *J* = 274.90 Hz), 109.91 (d, *J* = 22.58 Hz), 105.30 (d, *J* = 5.58 Hz), 100.91, 70.49, 70.44, 70.34, 70.30, 69.86, 69.66, 49.21, 41.55, 38.16, 31.35, 28.61, 28.45, 28.14, 23.51, 22.68, 16.28. HRMS (ESI) m/z $[M+H]^+$ calcd for $C_{41}H_{46}F_4N_7O_{11}$: 888.3191, found: 888.3192.

5-(3-(2,6-Dimethyl-4-(5-(trifluoromethyl)-1,2,4-oxadiazol-3-yl)phenoxy)propyl)-N-(1-(2-(2,6-dioxopiperidin-3-yl)-1-oxoisoindolin-5-yl)-4-oxo-9,12,15-trioxa-2,5-diazaoctadecan-18-yl)isoxazole-3-carboxamide (**Jun15692**). The title compound was prepared according to the GP-4 from 4f and ((2-(2,6-dioxopiperidin-3-yl)-1-oxoisoindolin-5-yl)methyl)glycine, as a white solid in 77% yield. ^1H NMR (400 MHz, $CDCl_3$) *δ* 9.10 (brs, 1H), 7.77 (s, 2H), 7.64 (s, 1H), 7.58 (t, *J* = 5.7 Hz, 2H), 7.52 (q, *J* = 7.9 Hz, 2H), 6.50 (s, 1H), 5.18 (dd, *J* = 13.6, 5.0 Hz, 1H), 4.35 (s, 4H), 3.91 (s, 1H), 3.88 (t, *J* = 6.0 Hz, 2H), 3.71 – 3.44 (m, 16H), 3.39 – 3.31 (m, 2H), 3.09 (t, *J* = 7.7 Hz, 2H), 2.96 – 2.78 (m, 3H), 2.32 (s, 6H), 2.26 – 2.17 (m, 3H), 1.87 – 1.81 (m, 2H), 1.76 – 1.68 (m, 2H). ^{13}C NMR (101 MHz, $CDCl_3$) *δ* 174.76, 171.72, 170.36, 168.97, 168.87, 165.59 (q, *J* = 44.74 Hz), 164.70, 160.15, 159.54, 159.06, 158.52, 142.50, 134.74, 132.24, 132.04, 130.16, 128.47, 120.30, 116.89, 116.01 (q, *J* = 274.79 Hz) 100.84, 70.52, 70.20, 70.13, 70.00, 69.62, 69.35, 69.35, 52.20, 51.44, 48.17, 47.47, 38.72, 38.28, 37.69, 31.32, 28.84, 28.25, 28.15, 23.53, 22.94, 16.29. HRMS (ESI) m/z $[M+H]^+$ calcd for $C_{44}H_{54}F_3N_8O_{11}$: 927.3864, found: 927.3870.

5-(3-(2,6-Dimethyl-4-(5-(trifluoromethyl)-1,2,4-oxadiazol-3-yl)phenoxy)propyl)-N-(1-(4-((2,6-dioxopiperidin-3-yl)oxy)phenyl)-1-oxo-6,9,12-trioxa-2-azapentadecan-15-yl)isoxazole-3-carboxamide (**Jun15693**). The title compound was prepared according to the GP-4 from 4f and 4-((2,6-dioxopiperidin-3-yl)oxy)benzoic acid, as a white solid in 68% yield. ^1H NMR (400 MHz, $CDCl_3$) *δ* 8.56 (brs, 1H), 7.80 – 7.75 (m, 4H), 7.63 (t, *J* = 5.6 Hz, 2H), 7.06 (d, *J* = 8.5 Hz, 2H), 6.53 (s, 1H), 4.98 (t, *J* = 6.5 Hz, 1H), 3.88 (t, *J* = 6.0 Hz, 2H), 3.70 – 3.58 (m, 10H), 3.54 – 3.46 (m, 6H), 3.10 (t, *J* = 7.6 Hz, 2H), 2.99 – 2.90 (m, 1H), 2.77 – 2.68 (m, 1H), 2.38 – 2.30 (m, 8H), 2.26 – 2.19 (m, 2H), 1.90 (p, *J* = 5.6 Hz, 2H), 1.82 (p, *J* = 6.2 Hz, 2H). ^{13}C NMR (101 MHz, $CDCl_3$) *δ* 174.67, 171.38, 169.56,

168.86, 168.06, 166.24, 165.58 (q, *J* = 44.67 Hz), 160.19, 159.48, 159.03, 158.49, 132.02, 129.16, 128.48, 127.53, 120.30, 115.68, 114.64, 100.97, 72.67, 70.87, 70.47, 70.32, 70.21, 70.14, 70.13, 69.70, 39.80, 37.92, 28.76, 28.63, 28.34, 28.15, 24.59, 23.54, 16.30. HRMS (ESI) m/z $[M+H]^+$ calcd for $C_{40}H_{48}F_3N_6O_{11}$: 845.3333, found: 845.3339.

5-(3-(2,6-Dimethyl-4-(5-(trifluoromethyl)-1,2,4-oxadiazol-3-yl)phenoxy)propyl)-N-(1-(2-(2,6-dioxopiperidin-3-yl)-1,3-dioxoisoindolin-5-yl)-1-oxo-6,9,12-trioxa-2-azapentadecan-15-yl)isoxazole-3-carboxamide (**Jun15701**). The title compound was prepared according to the GP-4 from 4f and thalidomide-5-COOH, as a green solid in 53% yield. ^{1}H NMR (400 MHz, $CDCl_3$) δ 8.91 (brs, 1H), 8.30 – 8.23 (m, 2H), 7.94 (d, *J* = 7.3 Hz, 2H), 7.78 (s, 2H), 7.66 (t, *J* = 5.8 Hz, 1H), 6.53 (s, 1H), 5.04 (dd, *J* = 12.3, 5.2 Hz, 1H), 3.89 (t, *J* = 6.0 Hz, 2H), 3.74 – 3.59 (m, 10H), 3.54 – 3.44 (m, 6H), 3.11 (t, *J* = 7.7 Hz, 2H), 2.94 – 2.75 (m, 3H), 2.33 (s, 6H), 2.27 – 2.16 (m, 3H), 1.94 (p, *J* = 6.0 Hz, 2H), 1.83 (p, *J* = 6.2 Hz, 2H). ^{13}C NMR (101 MHz, $CDCl_3$) δ 174.68, 171.30, 168.83, 168.34, 166.46, 165.77, 165.33, 159.52, 159.01, 158.42, 140.34, 133.95, 133.67, 132.01, 131.88, 128.45, 124.04, 122.26, 120.27, 115.98 (q, *J* = 274.91 Hz), 100.96, 70.78, 70.46, 70.18, 70.10, 70.04, 69.52, 49.54, 39.91, 37.82, 31.31, 28.64, 28.27, 28.12, 23.52, 22.46, 16.28. HRMS (ESI) m/z $[M+H]^+$ calcd for $C_{42}H_{47}F_3N_7O_{12}$: 898.3235, found: 898.3240.

5-(3-(2,6-Dimethyl-4-(5-(trifluoromethyl)-1,2,4-oxadiazol-3-yl)phenoxy)propyl)-N-(19-(2-(2,6-dioxopiperidin-3-yl)-1-oxoisoindolin-5-yl)-15-oxo-4,7,10-trioxa-14-azanonadec-18-yn-1-yl)isoxazole-3-carboxamide (**Jun15702**). The title compound was prepared according to the GP-4 from 4f and 5d, as a white solid in 36% yield. ^{1}H NMR (400 MHz, $CDCl_3$) δ 8.63 (brs, 1H), 7.81 – 7.76 (m, 3H), 7.64 (t, *J* = 5.8 Hz, 1H), 7.50 – 7.46 (m, 2H), 7.19 (t, *J* = 5.4 Hz, 1H), 6.54 (s, 1H), 5.25 (dd, *J* = 13.2, 5.1 Hz, 1H), 4.48 (d, *J* = 16.5 Hz, 1H), 4.34 (d, *J* = 16.4 Hz, 1H), 3.88 (t, *J* = 5.9 Hz, 2H), 3.72 – 3.65 (m, 4H), 3.64 – 3.52 (m, 10H), 3.44 (q, *J* = 5.9 Hz, 2H), 3.11 (t, *J* = 7.7 Hz, 2H), 2.96 – 2.83 (m, 2H), 2.79 (t, *J* = 7.2 Hz, 2H), 2.57 (t, *J* = 7.2 Hz, 2H), 2.39 – 2.29 (m, 7H), 2.27 – 2.19 (m, 3H), 1.90 – 1.77 (m, 4H). ^{13}C NMR (101 MHz, $CDCl_3$) δ 174.75, 172.97, 171.39, 169.59, 169.43, 168.84, 165.57 (q, *J* = 44.4 Hz), 159.61, 159.01, 158.37, 141.48, 132.00, 131.86, 130.07, 128.46, 127.82, 126.04, 124.02, 120.30, 115.98 (q, *J* = 274.76 Hz), 100.88, 91.10, 81.08, 70.45, 70.31, 70.28, 70.25, 70.12, 69.90, 69.75, 52.13, 47.21, 38.92, 38.02, 35.07, 31.38,

28.66, 28.26, 28.14, 23.52, 23.22, 16.27, 16.08. HRMS (ESI) m/z $[M+H]^+$ calcd for $C_{46}H_{53}F_3N_7O_{11}$: 936.3755, found: 936.3755.

5-(3-(2,6-Dimethyl-4-(5-(trifluoromethyl)-1,2,4-oxadiazol-3-yl)phenoxy)propyl)-N-(3-(2-(2-(3-((2-(1-methyl-2,6-dioxopiperidin-3-yl)-1,3-dioxoisoindolin-5-yl)amino)propoxy)ethoxy)ethoxy)propyl)isoxazole-3-carboxamide (**Jun15551**). The title compound was prepared according to the GP-5 from 4f and 5-fluoro-2-(1-methyl-2,6-dioxo-3-piperidinyl)-1*H*-Isoindole-1,3(2*H*)-dione, as a green solid in 40% yield. ^{1}H NMR (400 MHz, $CDCl_3$) δ 7.78 (s, 2H), 7.59 (d, *J* = 8.3 Hz, 1H), 7.39 (t, *J* = 5.5 Hz, 1H), 6.96 (d, *J* = 2.2 Hz, 1H), 6.74 (dd, *J* = 8.4, 2.2 Hz, 1H), 6.51 (s, 1H), 5.51 (t, *J* = 5.3 Hz, 1H), 4.94 (dd, *J* = 12.2, 5.3 Hz, 1H), 3.88 (t, *J* = 6.0 Hz, 2H), 3.74 – 3.68 (m, 4H), 3.66 – 3.60 (m, 8H), 3.55 (d, *J* = 6.1 Hz, 2H), 3.38 – 3.33 (m, 2H), 3.19 (s, 3H), 3.10 (t, *J* = 7.6 Hz, 2H), 2.99 – 2.91 (m, 1H), 2.83 – 2.70 (m, 2H), 2.33 (s, 6H), 2.26 – 2.21 (m, 2H), 2.11 – 2.06 (m, 1H), 1.95 – 1.86 (m, 4H). ^{13}C NMR (101 MHz, $CDCl_3$) δ 174.20, 171.31, 169.12, 168.86, 168.15, 167.63, 159.07, 158.96, 158.86, 153.97, 134.68, 132.02, 128.48, 125.43, 120.30, 117.92, 115.84, 106.31, 100.90, 70.57, 70.49, 70.45, 70.24, 69.92, 69.87, 49.80, 42.12, 37.84, 31.94, 28.93, 28.29, 28.21, 27.16, 23.51, 22.10, 16.34. HRMS (ESI) m/z $[M+H]^+$ calcd for $C_{42}H_{49}F_3N_7O_{11}$: 884.3442, found: 884.3452.

5-(3-(2,6-Dimethyl-4-(5-(trifluoromethyl)-1,2,4-oxadiazol-3-yl)phenoxy)propyl)-N-(19-(2-(1-methyl-2,6-dioxopiperidin-3-yl)-1-oxoisoindolin-5-yl)-15-oxo-4,7,10-trioxa-14-azanonadec-18-yn-1-yl)isoxazole-3-carboxamide (**Jun15953**). The title compound was prepared according to the GP-4 from 4f and 5e, as a white solid in 39% yield. ^{1}H NMR (400 MHz, $CDCl_3$) δ 7.87 – 7.69 (m, 3H), 7.55 – 7.43 (m, 3H), 7.01 (t, *J* = 5.3 Hz, 1H), 6.52 (s, 1H), 5.21 (dd, *J* = 13.5, 5.1 Hz, 1H), 4.45 (d, *J* = 16.3 Hz, 1H), 4.32 (d, *J* = 16.3 Hz, 1H), 3.88 (t, *J* = 6.0 Hz, 2H), 3.75 – 3.65 (m, 4H), 3.64 – 3.50 (m, 10H), 3.43 (q, *J* = 5.9 Hz, 2H), 3.18 (s, 3H), 3.10 (t, *J* = 7.7 Hz, 2H), 3.05 – 2.29 (m, 1H), 2.92 – 2.83 (m, 1H), 2.78 (t, *J* = 7.3 Hz, 2H), 2.55 (t, *J* = 7.3 Hz, 2H), 2.39 – 2.28 (m, 7H), 2.27 – 2.17 (m, 3H), 1.83 (m, 4H). ^{13}C NMR (101 MHz, $CDCl_3$) δ 174.62, 172.53, 171.14, 169.86, 169.30, 168.86, 165.19 (q, *J* = 37.8 Hz), 159.47, 159.04, 158.54, 141.48, 132.02, 131.79, 130.45, 128.48, 127.64, 126.00, 124.00, 120.32, 117.94 (q, *J* = 300.2 Hz), 100.85, 91.10, 81.05, 70.47, 70.26, 70.19, 69.97, 69.85, 69.66, 52.70, 47.19, 38.51, 37.73, 35.17, 31.95, 28.82,

28.47, 28.18, 27.18, 23.54, 22.67, 16.31, 16.05. HRMS (ESI) m/z $[M+H]^+$ calcd for $C_{47}H_{55}F_3N_7O_{11}$: 950.3912, found: 950.3918.

Chemicals, cells, and viruses. Pleconaril was purchased from Sigma-Aldrich (Cat # SML0307). Human rhabdomyosarcoma (RD; ATCC CCL-136) was maintained at 37 °C in a 5% CO_2 atmosphere and cultured in Dulbecco's modified Eagle's medium (DMEM) supplemented with 10% fetal bovine serum (FBS) and 1% penicillin−streptomycin (PS) antibiotics. The antibody (Rabbit Enterovirus D68 VP1 antibody) was purchased from GeneTex (Cat# GTX132313). The antibody (Alexa Flour 488 goat anti-rabbit IgG) was purchased from Fisher Scientific (Cat # A11034). Trans-Blot Turbo Packs (Cat# 1704156) and other consumables, including 2× Laemmli sample buffer (Bio-Rad, #1610737), Pierce 660 nm Protein Assay Kit (Thermo Fisher Scientific, #22660), 5% nonfat dry milk (Santa Cruz Biotechnology, #sc-2324), horseradish peroxidase (HRP)-conjugated anti-rabbit IgG secondary antibody (Abcam, #ab6721, 1:2000), and HRP-conjugated anti-β-actin mouse antibody (Thermo Fisher Scientific, #MA5-15739-HRP, 1:2000), and Pierce ECL Western Blotting Substrate (Thermo Fisher Scientific, #32106) for western blot were purchased accordingly. Hoechst dye for DNA stains was purchased from Biotium Cat# 40046). The following EV-D68 viruses were obtained through BEI Resources, NIAID, NIH: EV-D68 US/MO/14-18949, NR-49130; EV-D68 US/MO/14-18947, NR-49129; EV-D68 US/IL/14-18952, NR-49131; EV-D68 US/KY/14-18953, NR-49132; EV-D68 US/IL/14-18956, NR-49133. All viruses were amplified in RD cells prior to infection assays.

Cytopathic effect (CPE) assay. The CPE assay was performed with RD cells using the neutral red uptake method.[1, 2] RD cells (about 84,000 cells/mL) grown in DMEM with 10% FBS and 1% PS were dispensed into 96-well cell culture plates at 100μL/well. The plates were incubated at 37 °C in a 5% CO_2 atmosphere for 24 h. Then, the growth DMEM medium was aspirated and washed with 100 μL PBS buffer. After aspiration, the cells were infected with the virus diluted in 100 μL DMEM with 2% FBS, 1% PS antibiotic, and 30 mM $MgCl_2$. The plates were then incubated for 1 h at 33 °C in a 5% CO_2 atmosphere, followed by adding 1 μL testing compounds and 100 μL fresh DMEM medium with 2% FBS, 1% PS antibiotic, and 30 mM $MgCl_2$. After 60 h of incubation at 33 °C in a 5% CO_2 atmosphere, the medium was aspirated and replaced with 100 μL serum-free DMEM

medium containing 66 μg/mL neutral red and incubated at 37 °C in a 5% CO_2 atmosphere for 2 h.[1] After aspiration and washing with 100 μL PBS buffer, 100 μL neutral red destain solution was added to extract the neutral red from the cells.[1] The plate was put on an orbital shaker until a homogeneous solution formed in each well, and the amount of neutral red taken up was determined by measuring the absorbance at 540 nm using a BioTek Microplate Epoch 2 Reader (Agilent). The EC_{50} values were calculated from best-fit dose-response curves with the variable slope using GraphPad Prism software.

Cytotoxicity assay. The cytotoxicity of compounds was determined following a similar procedure of the CPE assay without the virus infection. RD cells (about 84,000 cells/mL) grown in DMEM with 10% FBS and 1% PS were dispensed into 96-well cell culture plates at 100μL/well. The plates were incubated at 37 °C in a 5% CO_2 atmosphere for 24 h. After the washing, PBS buffer was aspirated, 100 μL fresh DMEM medium with 2% FBS and 1% PS was first added, followed by adding 1 μL serially diluted compounds. Another 100 μL fresh DMEM medium with 2% FBS and 1% PS was then added. After 60 h of incubation at 33 °C in a 5% CO_2 atmosphere, the cells were stained with 66 μg/mL neutral red in serum-free DMEM medium. The same procedure was followed as in the CPE assay to measure the absorbance at 540 nm. The CC_{50} values were calculated from best-fit dose-response curves with the variable slope using GraphPad Prism software.

Plaque assay. For plaque assay, RD cells (about 420,000 cells/mL) grown in DMEM with 10% FBS and 1% PS were dispensed into 6-well cell culture plates at 3 mL/well and incubated at 37 °C in a 5% CO_2 atmosphere. After 24 h, the growth DMEM medium was aspirated and washed with 2 mL PBS buffer. After aspiration, cells were infected with 500 μL of virus diluted in DMEM with 2% FBS, 1% PS antibiotic, and 30 mM $MgCl_2$. The plates were then incubated for 1 hour at 33 °C in a 5% CO_2 atmosphere. During the incubation, a 1.2% Avicel microcrystalline cellulose (FMC BioPolymer, Philadelphia, PA) overlay in DMEM media with 2% FBS, 30 mM $MgCl_2$, and different concentrations of compounds were prepared. After aspiration, 4 mL avicel overlay solution was added to each well. After 60 h incubation at 33 °C in a 5% CO_2 atmosphere, the overlay was aspirated in each well and washed with PBS. Then, the cells were stained with crystal

violet dye solution (0.2% crystal violet, 20% methanol). Plaque areas were quantified using ImageJ.[3]

Time-of-addition assay. The time-of-addition assay was performed following the procedure previously described.[4] Approximately 90% of confluent RD cells were infected with EV-D68 US/MO/14-18947 at the MOI of 1 for 1 hour, which occurred during 0 to 1 hpi. For full course time-of-addition experiments, the cells were treated with 10 μM VP1 PROTAC degraders or Pleconaril every one hour since 1 h before infection (-1 hpi). For the post-entry phase only (2 to 8 h), the testing degrader or Pleconaril was only present starting from 2 hpi. Cells were fixed at 8 hpi and processed for immunofluorescence analysis of intracellular VP1 levels.

Immunofluorescence Staining. RD cells grown on glass coverslips were fixed with 4% paraformaldehyde at 8 hpi. After permeabilization with 0.1% Triton X-100 with Glycine, cells were blocked in 3% BSA for overnight and then incubated with rabbit anti-EV-D68 VP1 antibody (1:500), followed by Alexa Fluor 488-conjugated secondary antibody. Nuclei were counterstained with Hoechst 33342. Images were acquired using a fluorescence microscope and quantified using ImageJ.

Western Blot Analysis. RD cells (approximately 4.2×10^5 cells/mL) were cultured in Dulbecco's modified Eagle's medium (DMEM) supplemented with 10% fetal bovine serum (FBS) and 1% penicillin–streptomycin (PS). Cells were seeded into 6-well plates at 3 mL per well and incubated overnight at 37 °C in a humidified atmosphere containing 5% CO_2. At approximately 90% confluency, cells were infected with EV-D68 (US/MO/14-18947) at a multiplicity of infection (MOI) of 1 for 1 h. Following viral adsorption, the inoculum was removed, cells were washed once with 2 mL of phosphate-buffered saline (PBS), and 4 mL of fresh viral diluent was added. Cells were incubated for an additional 1 h at 33 °C in 5% CO_2, after which test compounds or degraders were added at 2 h post-infection (hpi). Following an additional 8 h incubation, cells were lysed directly in 2× Laemmli sample buffer supplemented with 200 mM dithiothreitol (DTT). Lysates were boiled prior to storage at −80 °C. Protein concentrations were quantified using the Pierce 660 nm Protein Assay Kit, with samples diluted 50-fold in deionized water prior to measurement. Equal amounts of protein were

resolved by SDS-PAGE and transferred to membranes using a Trans-Blot Turbo Transfer System (Bio-Rad). Membranes were blocked with 5% nonfat dry milk in PBS containing 0.1% Tween-20 (PBS-T) for 2 h at room temperature. Membranes were incubated overnight at 4 °C with rabbit anti-EV-D68 VP1 primary antibody diluted 1:1000 in PBS-T. After washing, membranes were probed with horseradish peroxidase (HRP)-conjugated anti-rabbit IgG secondary antibody and HRP-conjugated anti-β-actin mouse antibody. Protein bands were visualized using Pierce ECL Western Blotting Substrate and imaged with the Invitrogen iBright Imaging System. Band intensities were quantified using ImageJ software, normalized to β-actin, and plotted using GraphPad Prism.

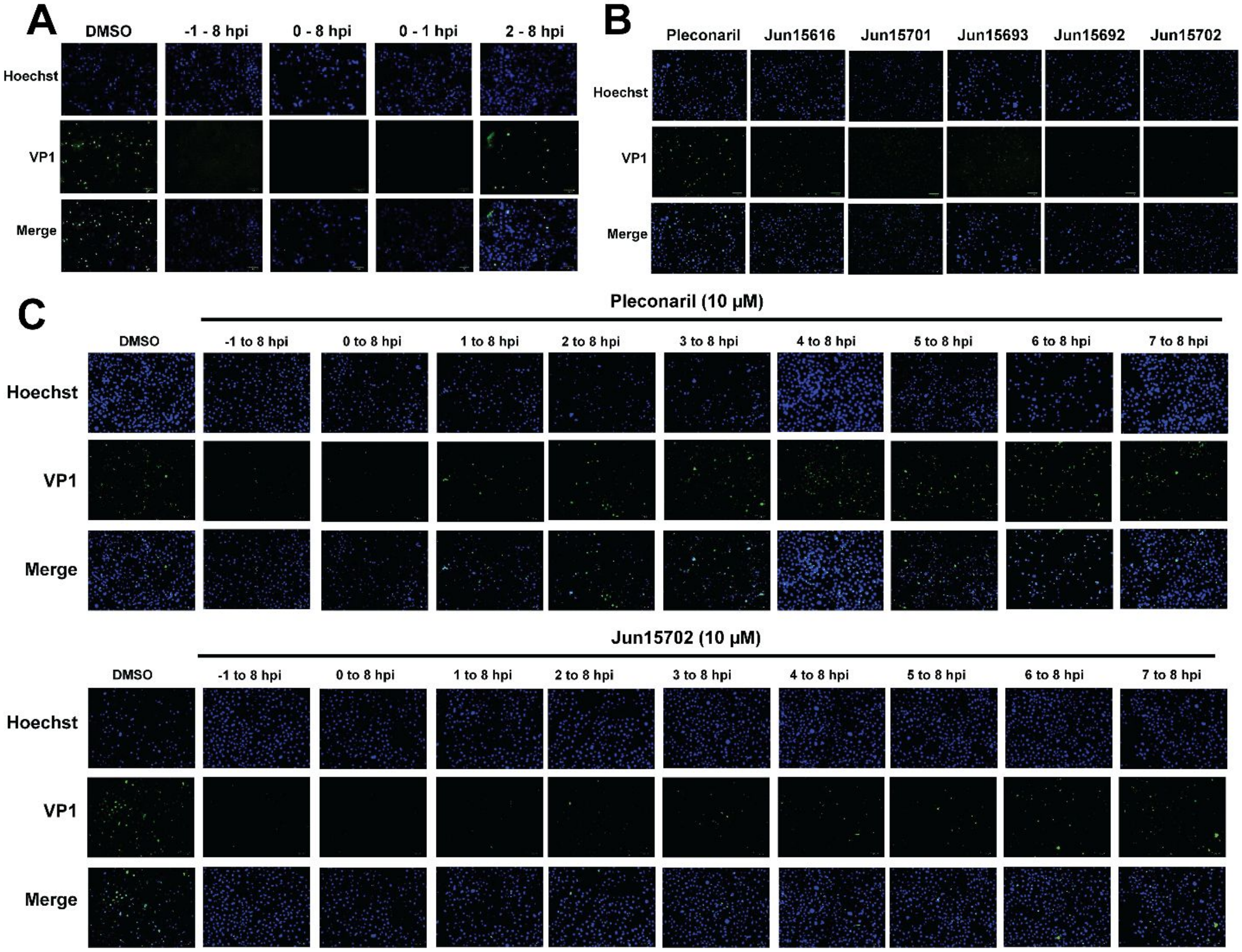

Figure S1. Time-of-addition assay and immunofluorescence assay to quantify intracellular EV-D68 capsid VP1 levels. (A) Time-of-addition assay performed with pleconaril to define the antiviral window of action. (B) Immunofluorescence analysis of VP1 following treatment with candidate VP1 PROTAC degraders during the post-entry phase (2-8 hpi). VP1 was detected using an Alexa Fluor 488-conjugated secondary antibody (green), and nuclei were counterstained with Hoechst (blue). Representative images from three independent experiments are shown. (C) Full-course time-of-addition assay comparing pleconaril and **Jun15702**.

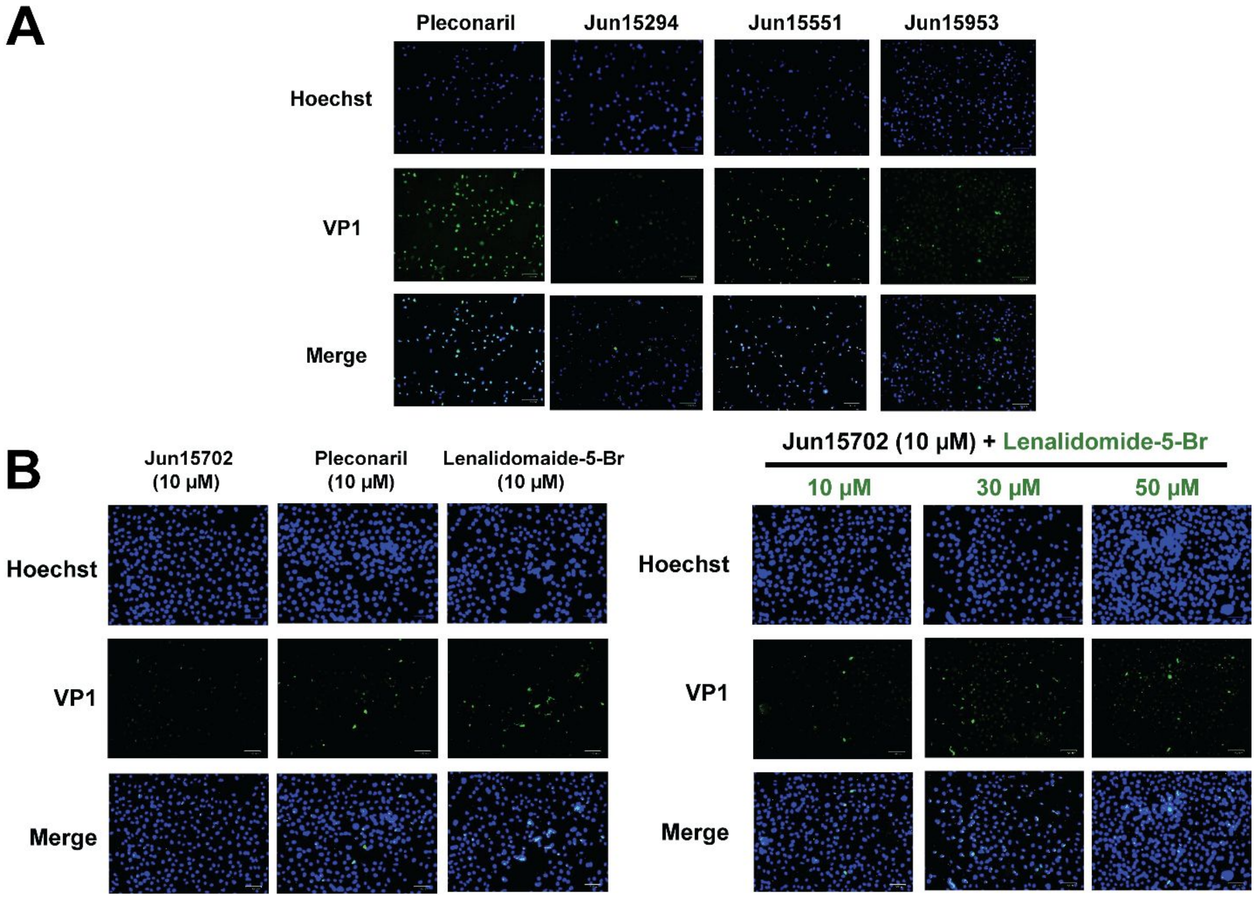

Figure S2. Immunofluorescence assay and competition assays with immunofluorescence staining to quantify intracellular EV-D68 capsid VP1 levels. (A) Immunofluorescence assay of VP1 following treatment with candidate VP1 PROTAC degraders during the post-entry phase (2-8 hpi). Representative images from three independent experiments are shown. (B) Competition assay with immunofluorescence staining to evaluate cotreatment of **Jun15702** with lenalidomide-5-Br during the post-entry phase (2-8 hpi). VP1 was detected using an Alexa Fluor 488-conjugated secondary antibody (green), and nuclei were counterstained with Hoechst (blue). Representative images from two independent experiments are shown.

Spectral Data

^{1}H NMR of 1

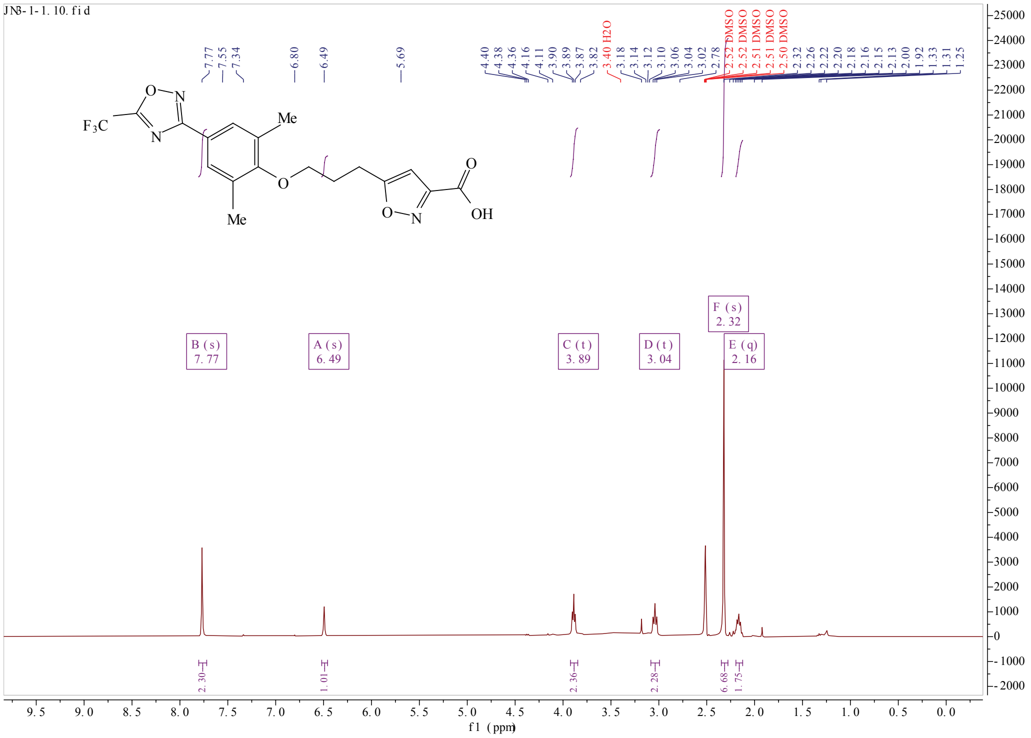

^{1}H NMR of 2a

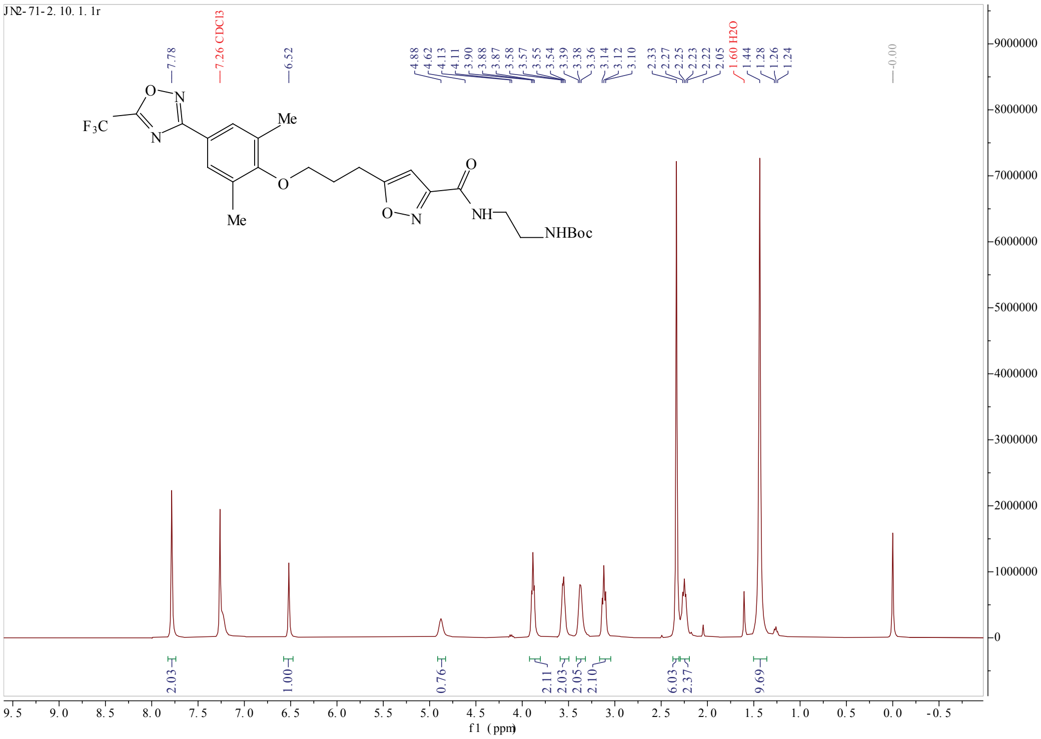

^{1}H NMR of 2b

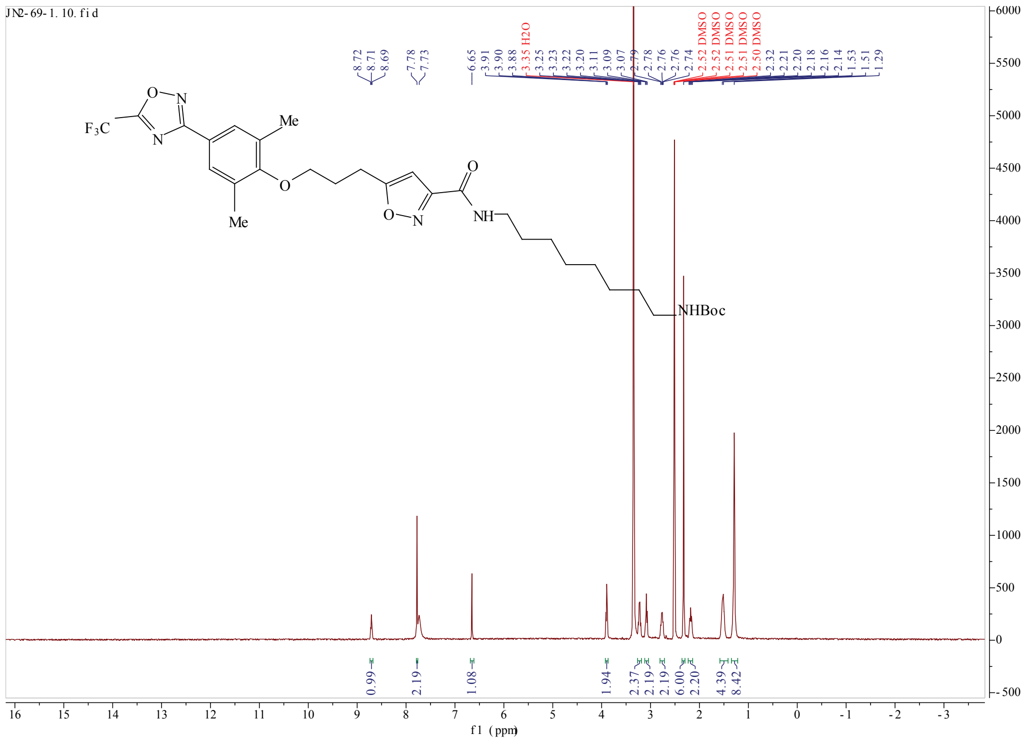

^{1}H NMR of 2c

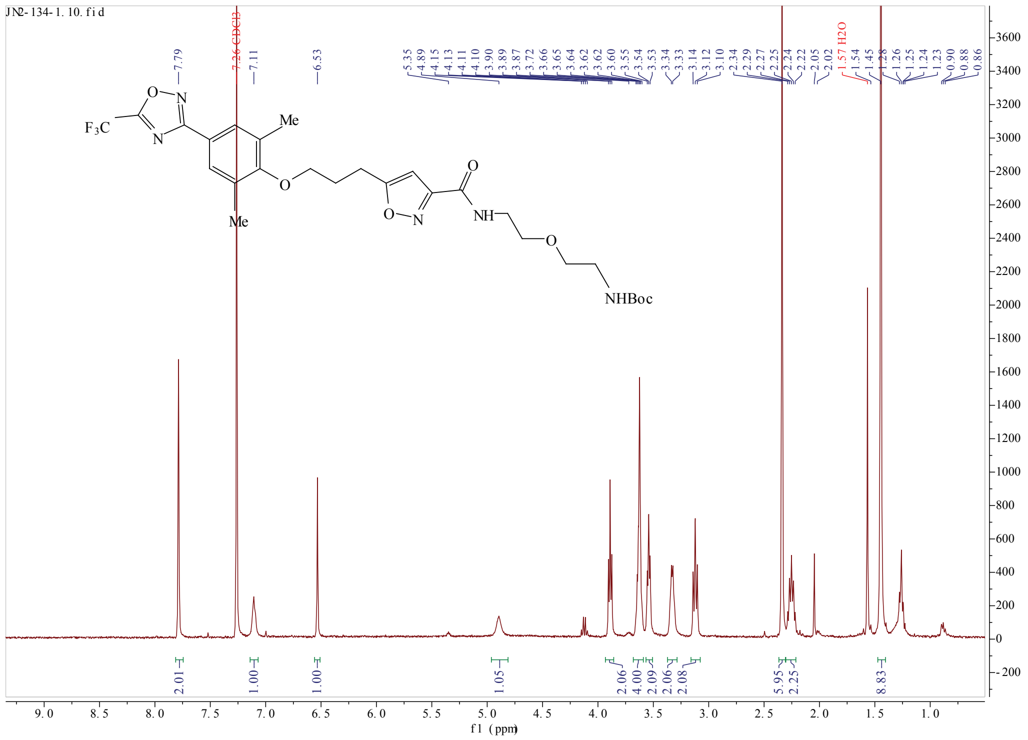

^{1}H NMR of 2d

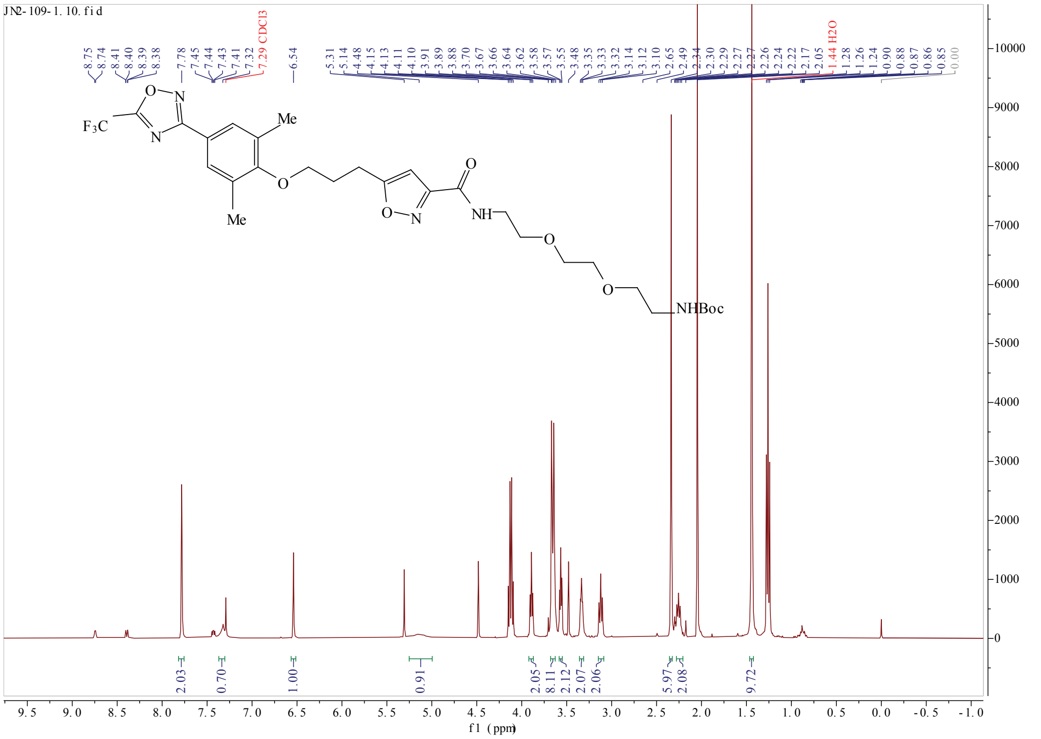

^{1}H NMR of 2e

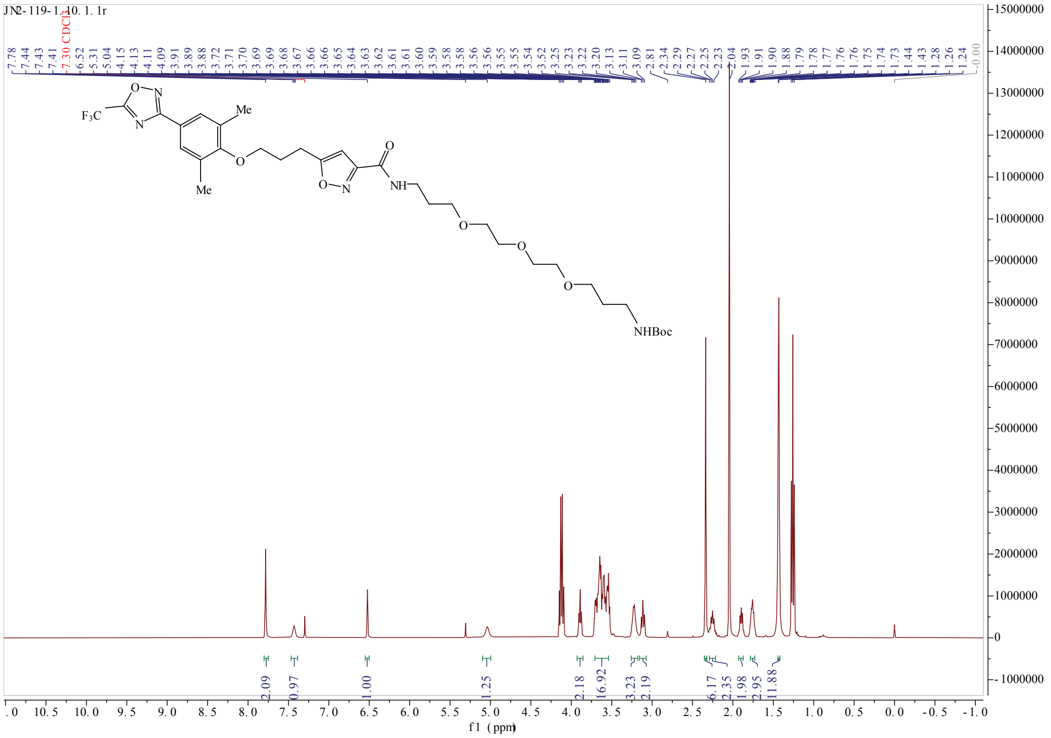

^{1}H NMR of 2f

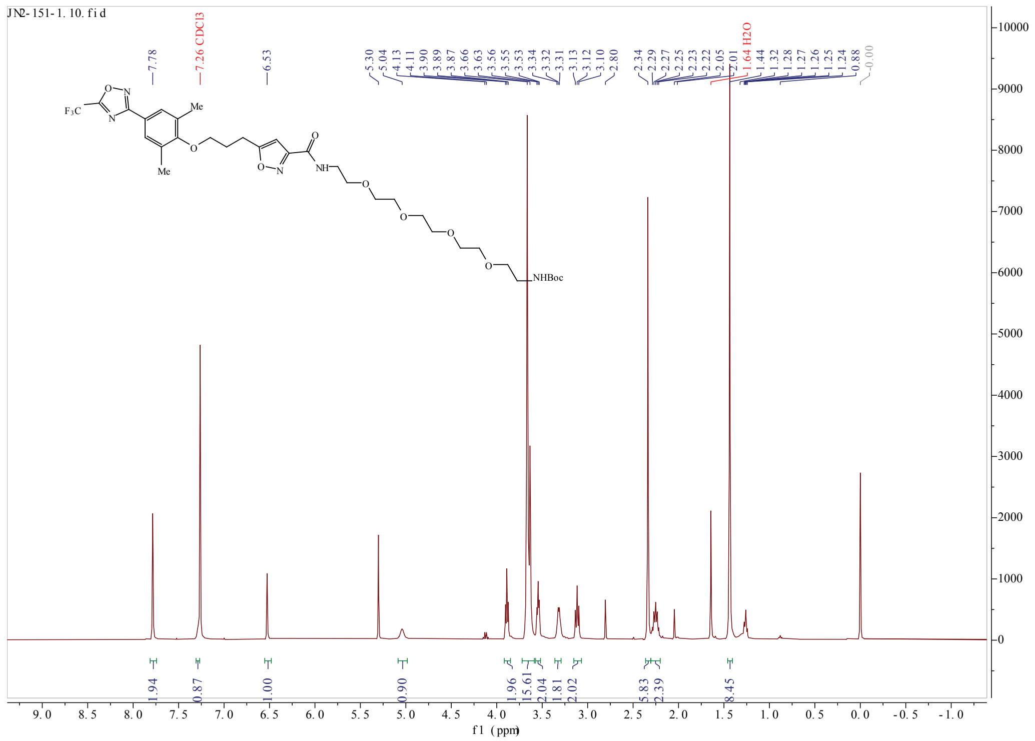

^{1}H NMR of 2g

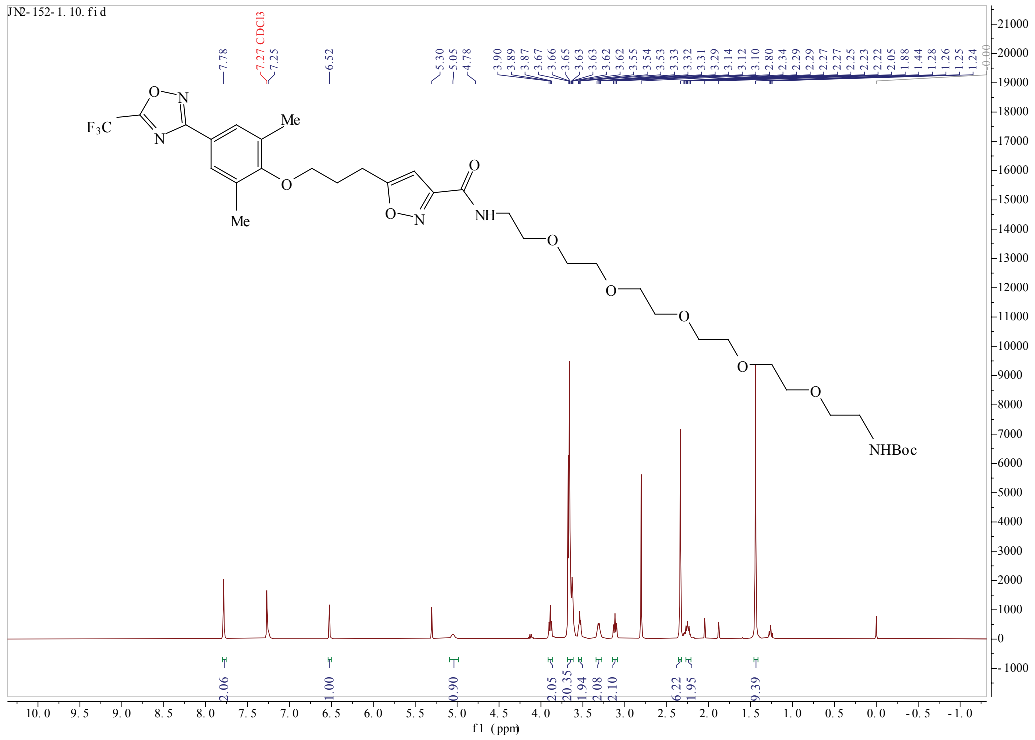

^{1}H NMR of 3a

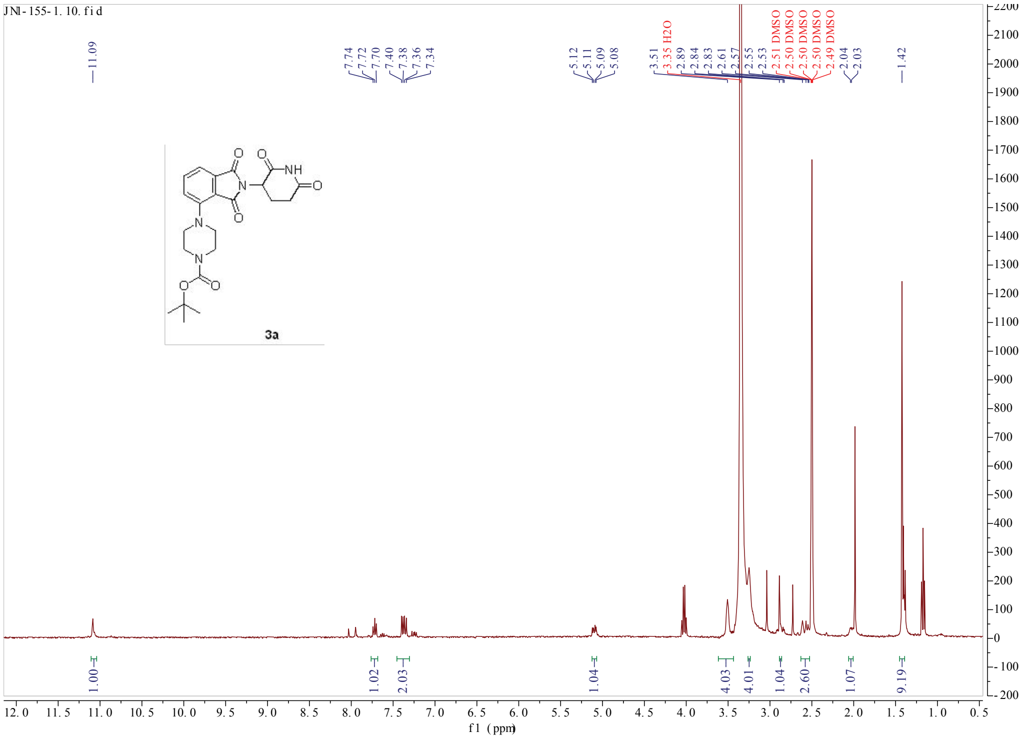

^{1}H NMR of 3b

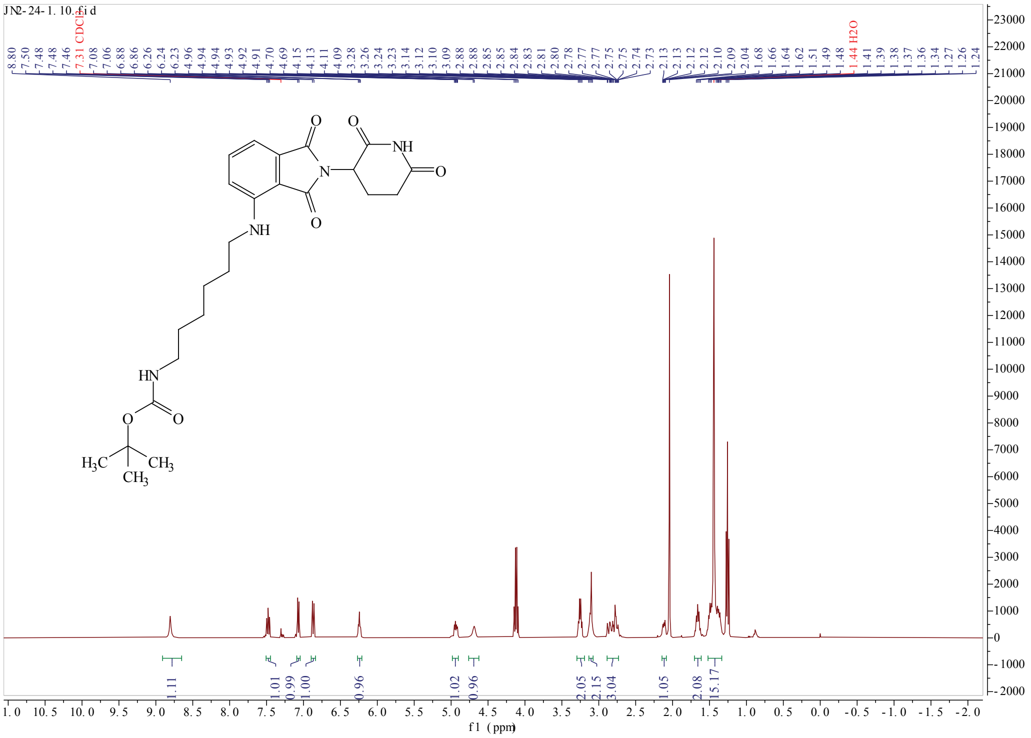

¹H NMR of 3c

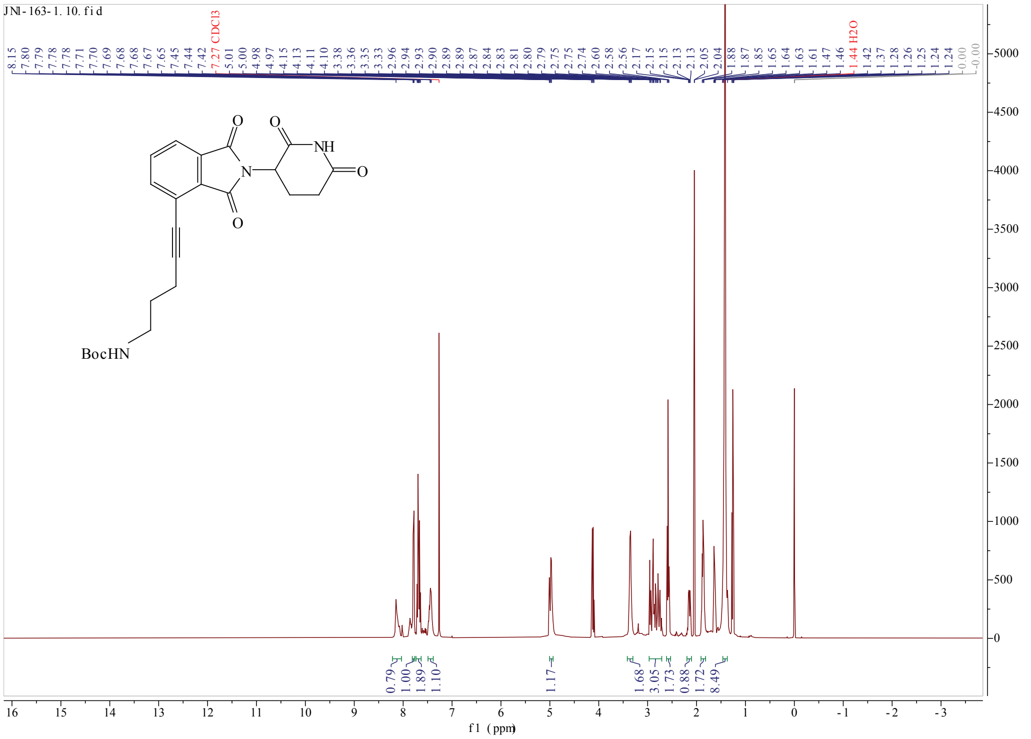

¹H NMR of 3d

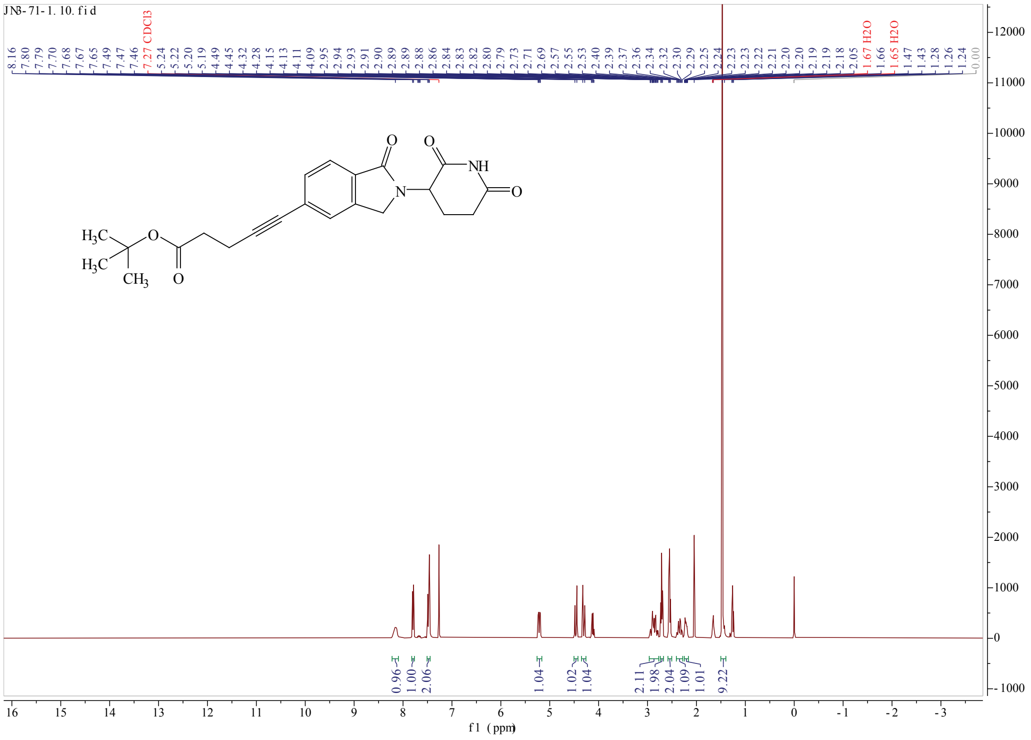

^{1}H NMR of 3e

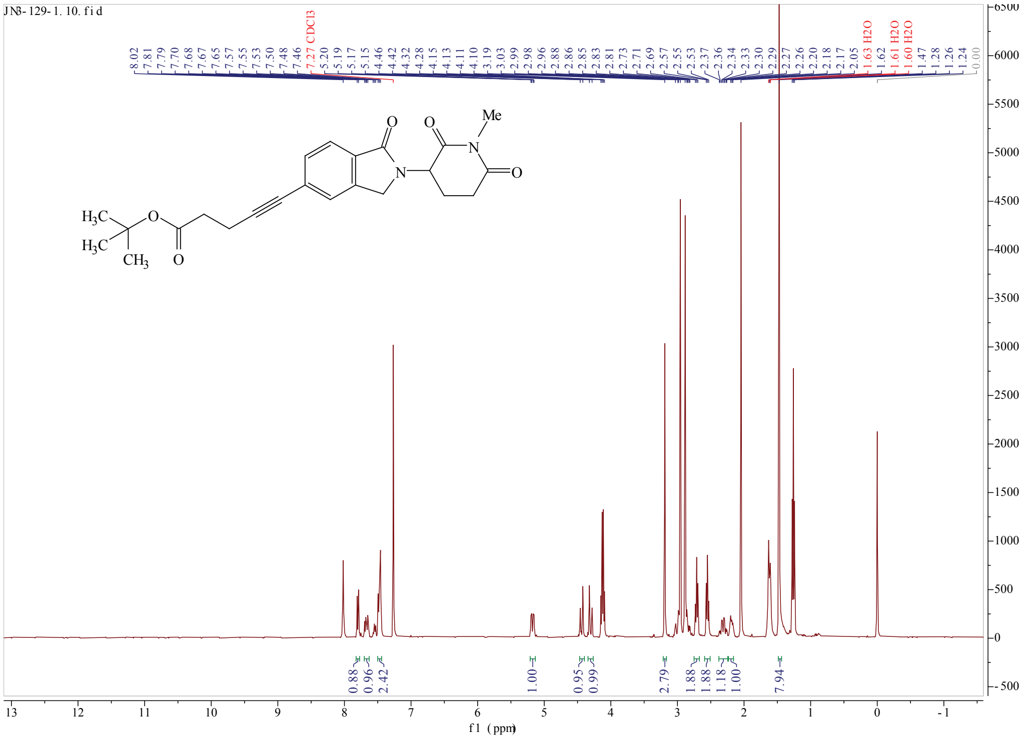

^{1}H NMR and ^{13}C NMR spectra of Jun14956

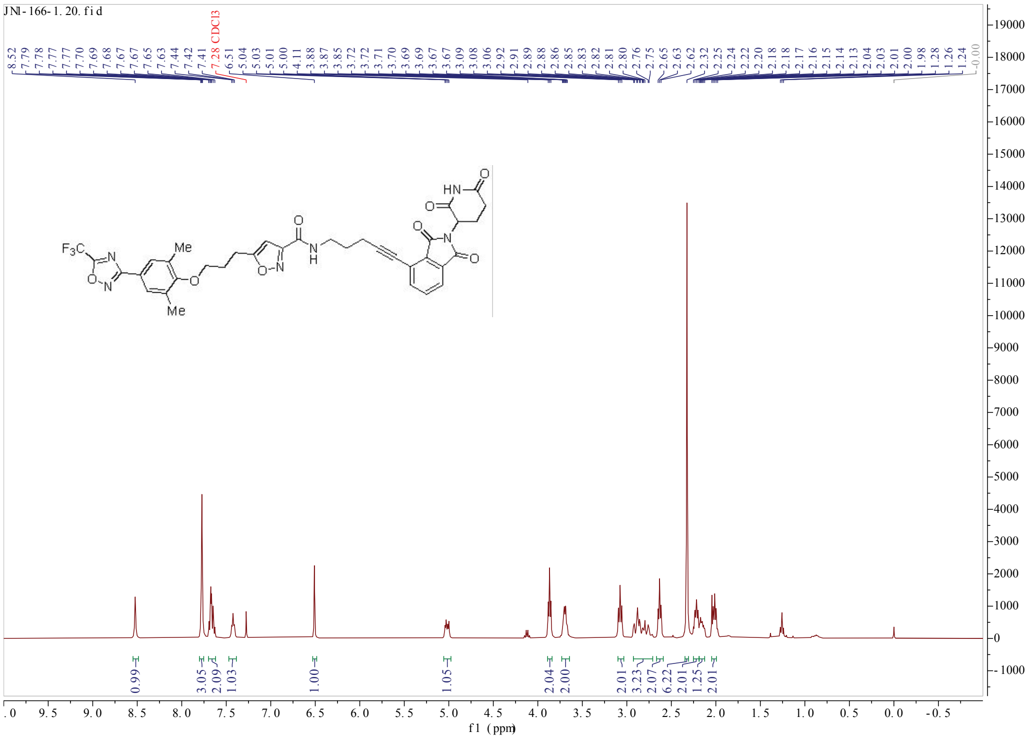

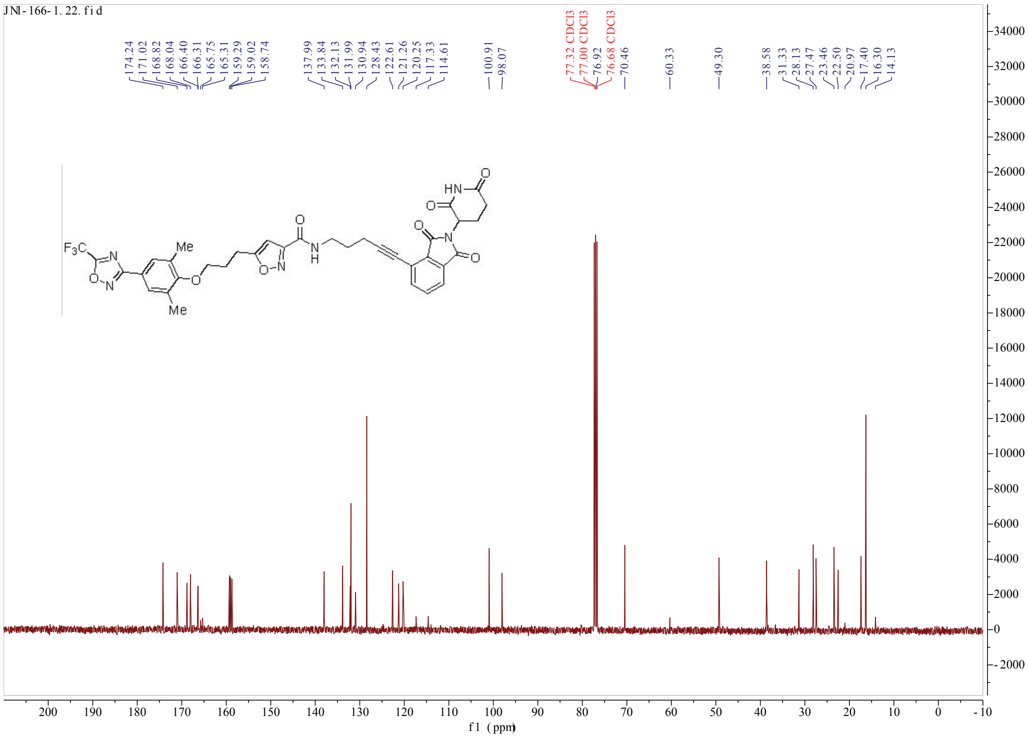

^1H NMR and ^{13}C NMR spectra of Jun1522

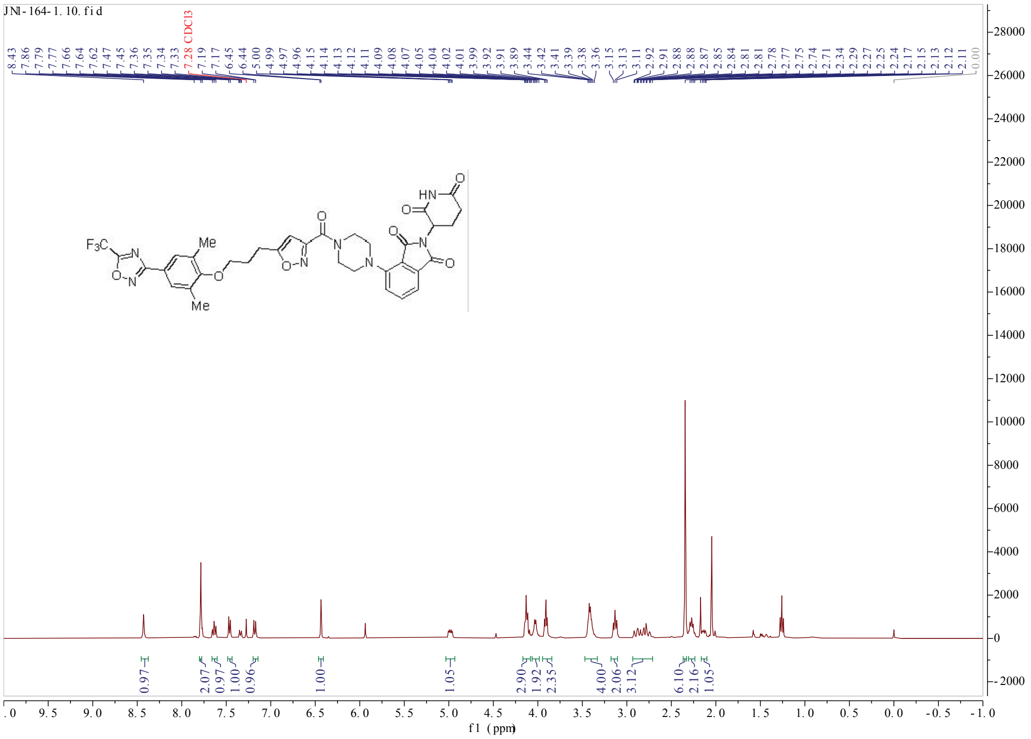

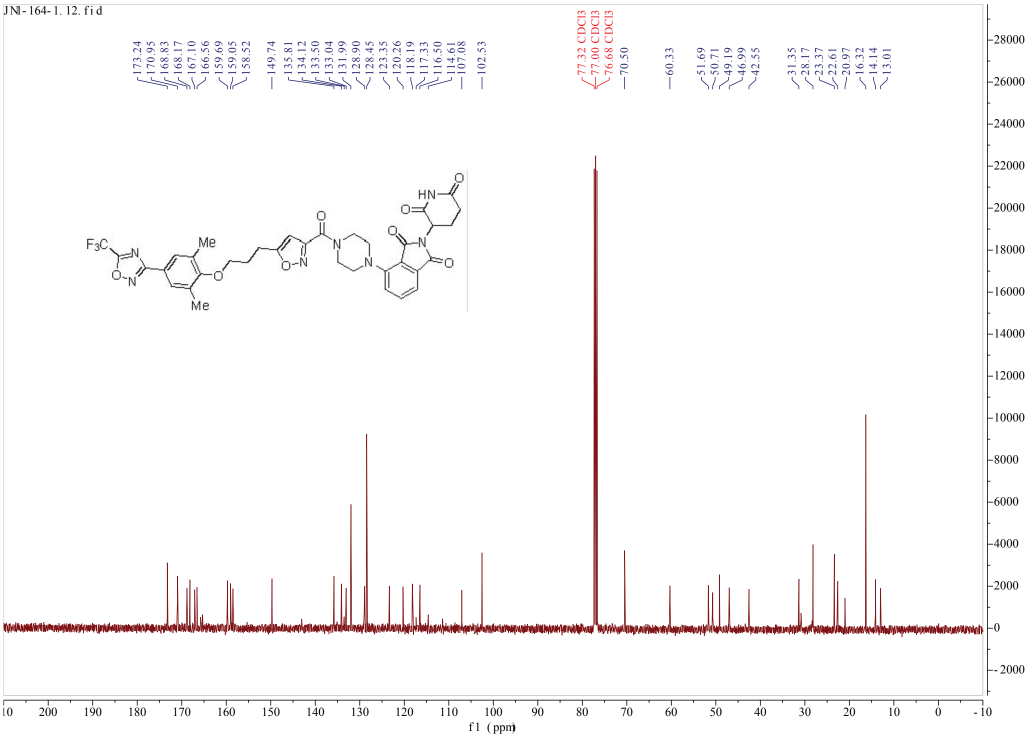

^{1}H NMR and ^{13}C NMR spectra of Jun1554

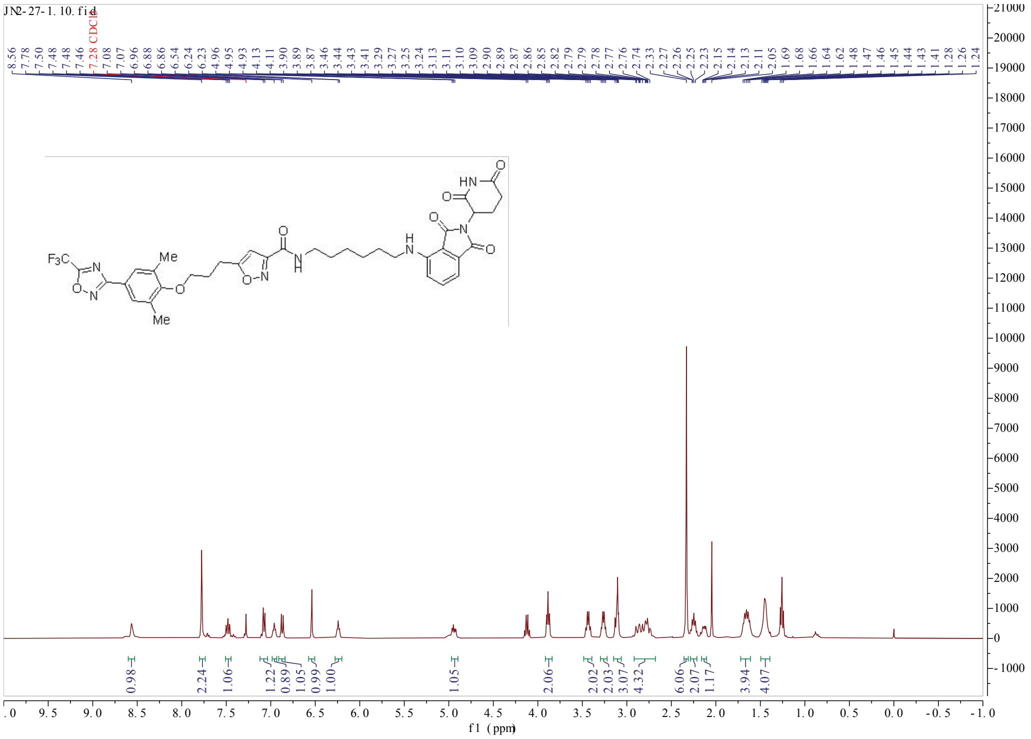

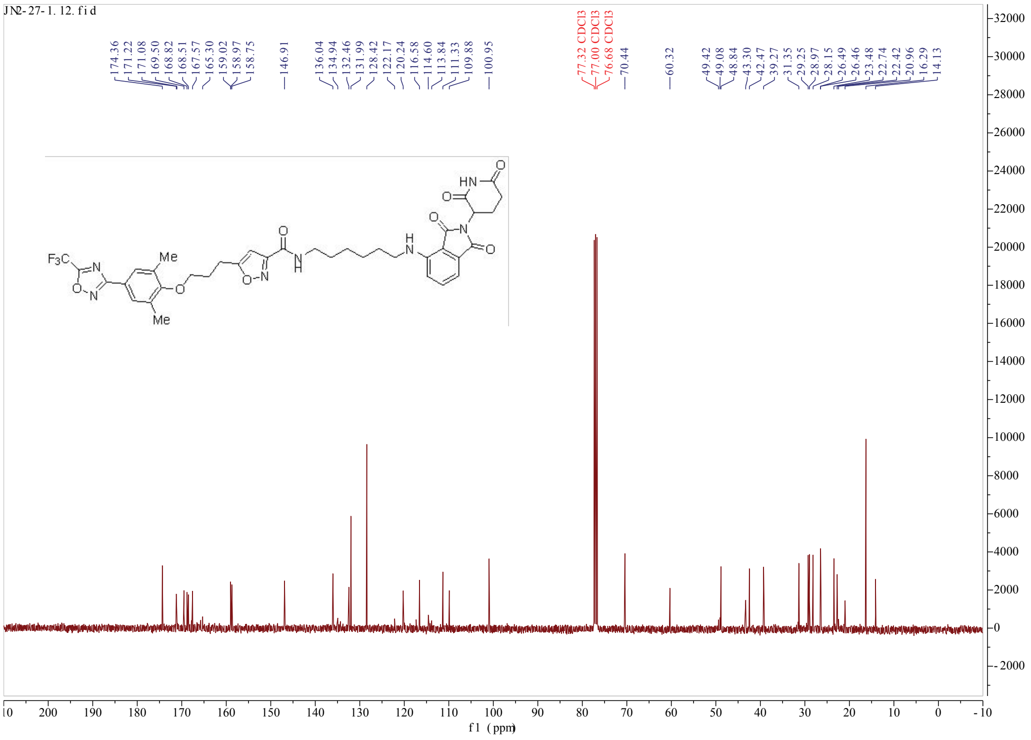

^{1}H NMR and ^{13}C NMR spectra of Jun15183

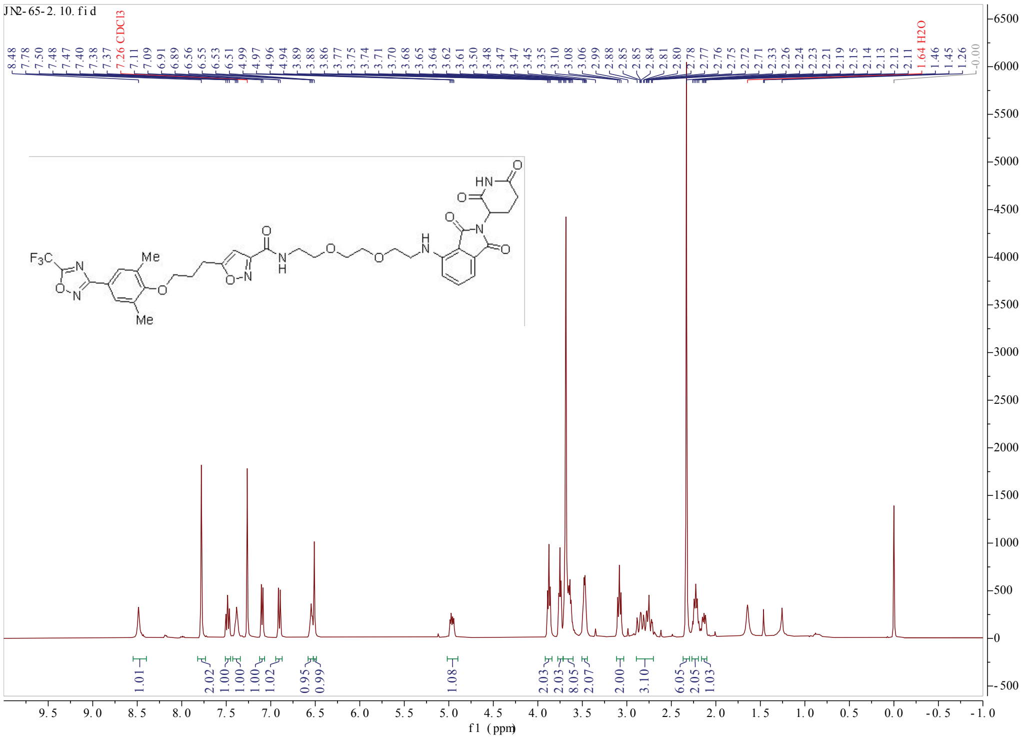

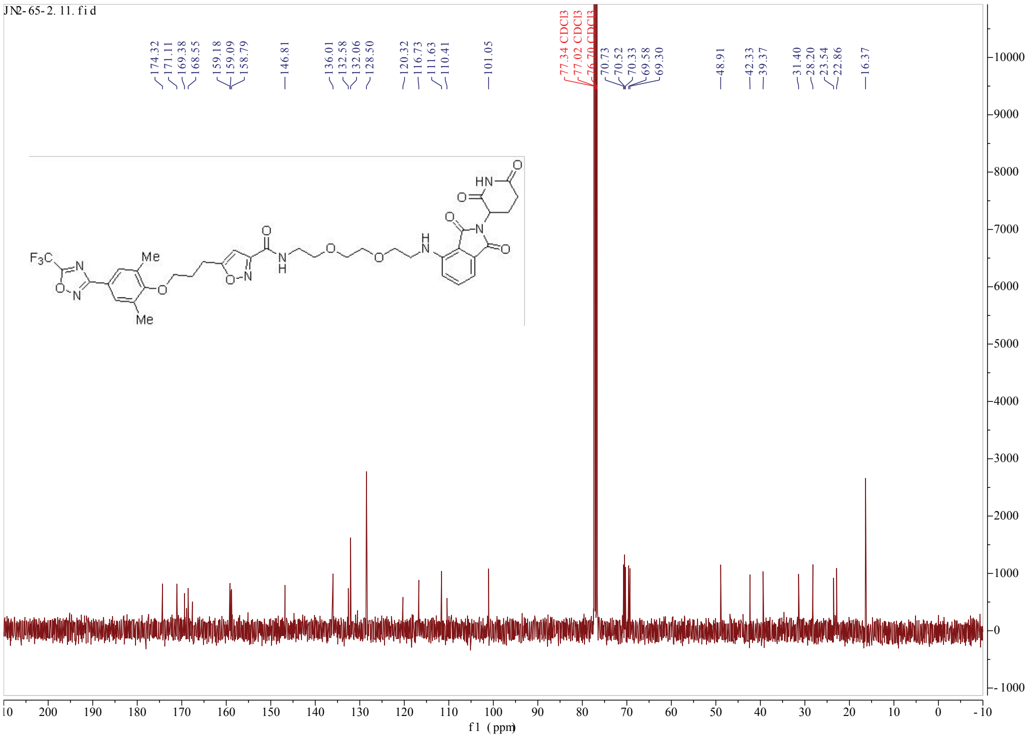

^{1}H NMR and ^{13}C NMR spectra of Jun15191

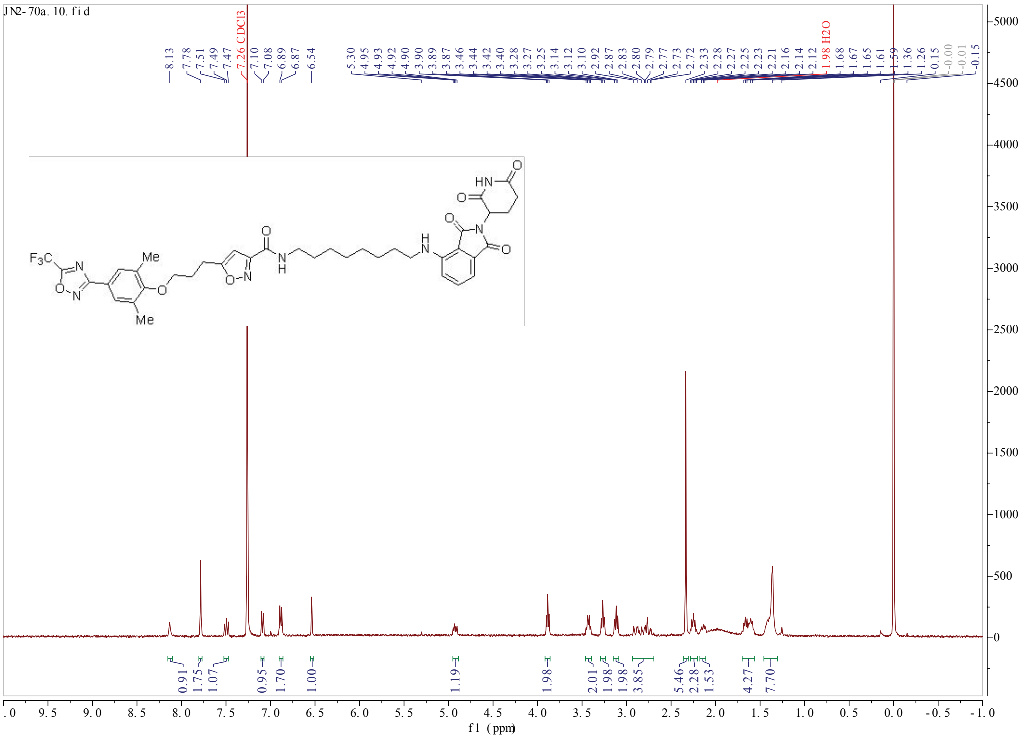

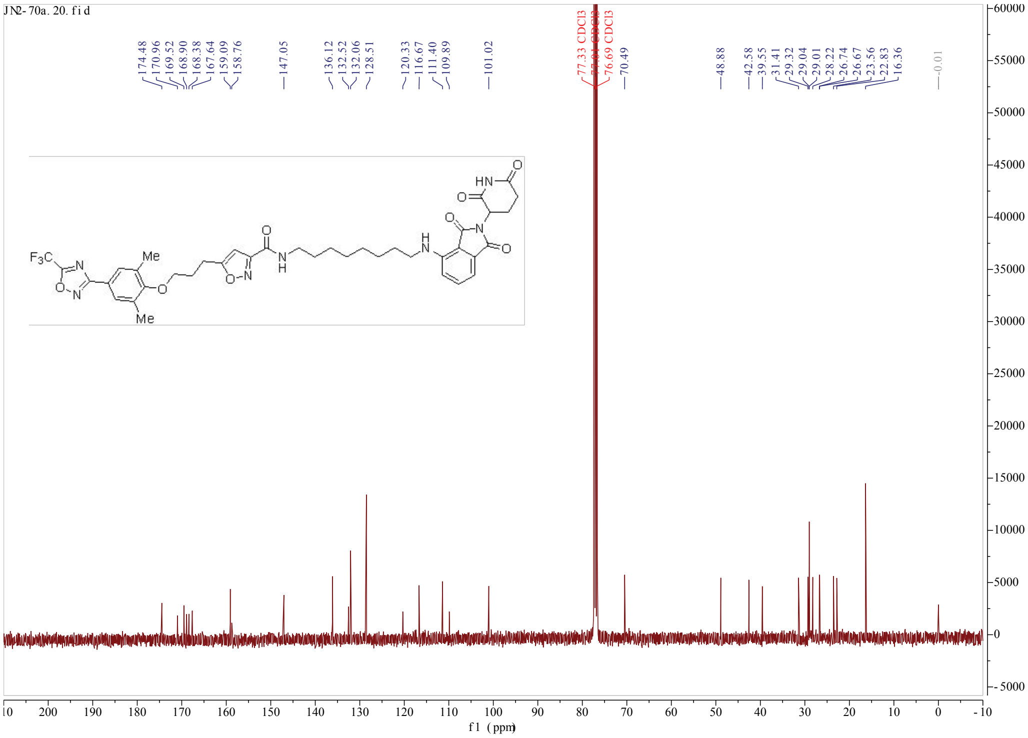

^{1}H NMR and ^{13}C NMR spectra of Jun15373

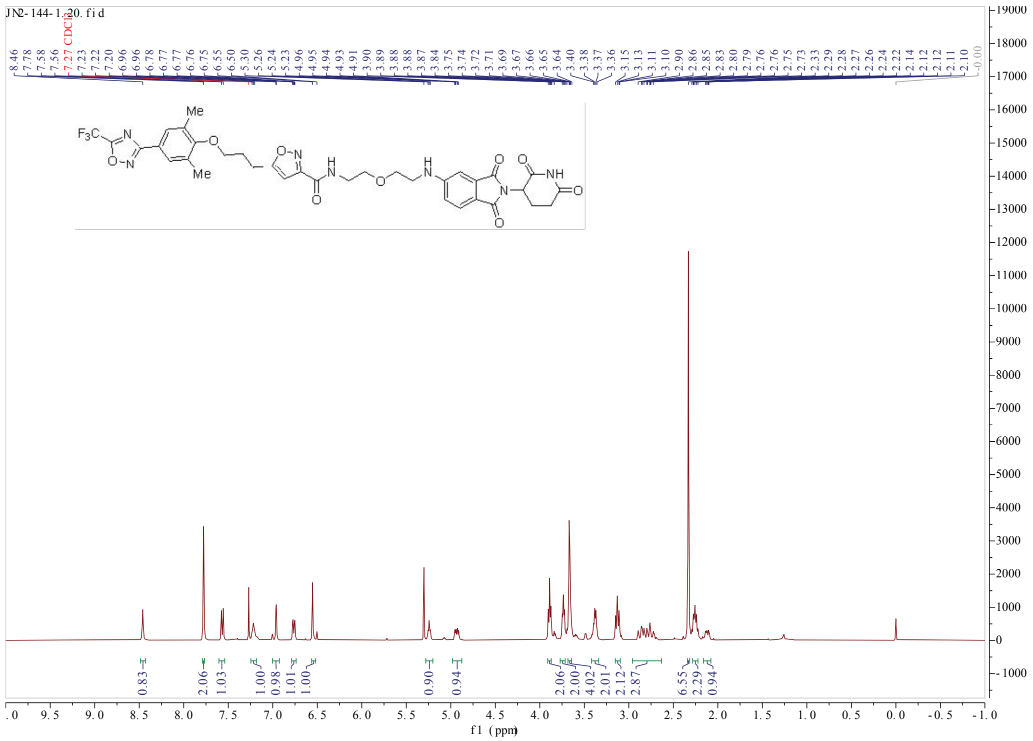

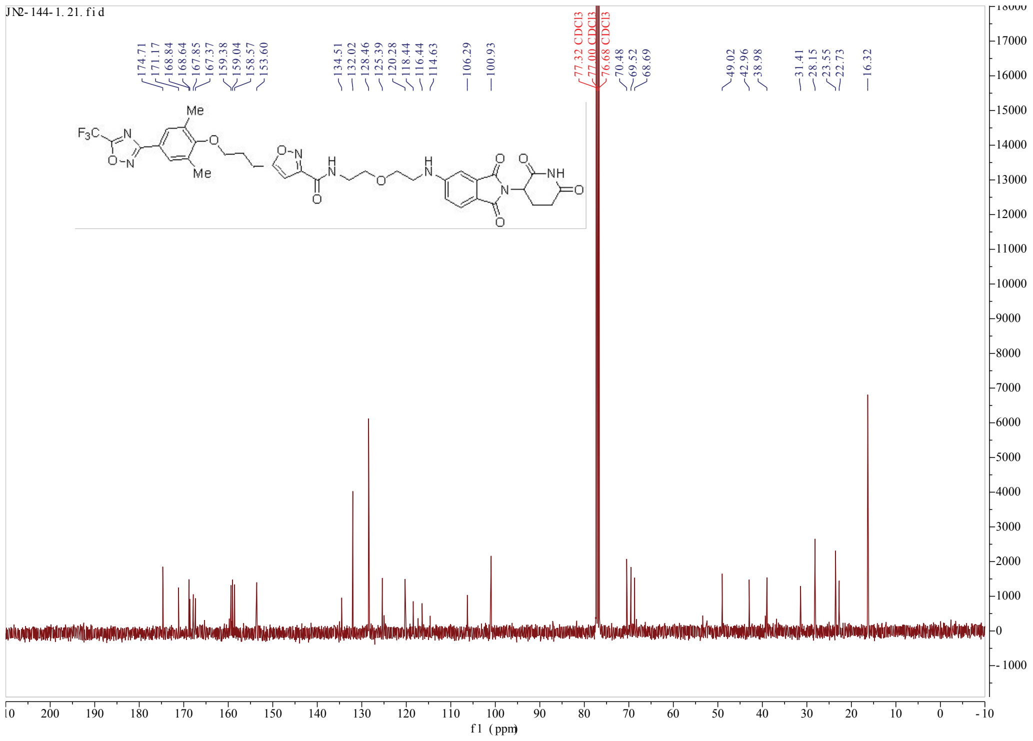

^{1}H NMR and ^{13}C NMR spectra of Jun15192

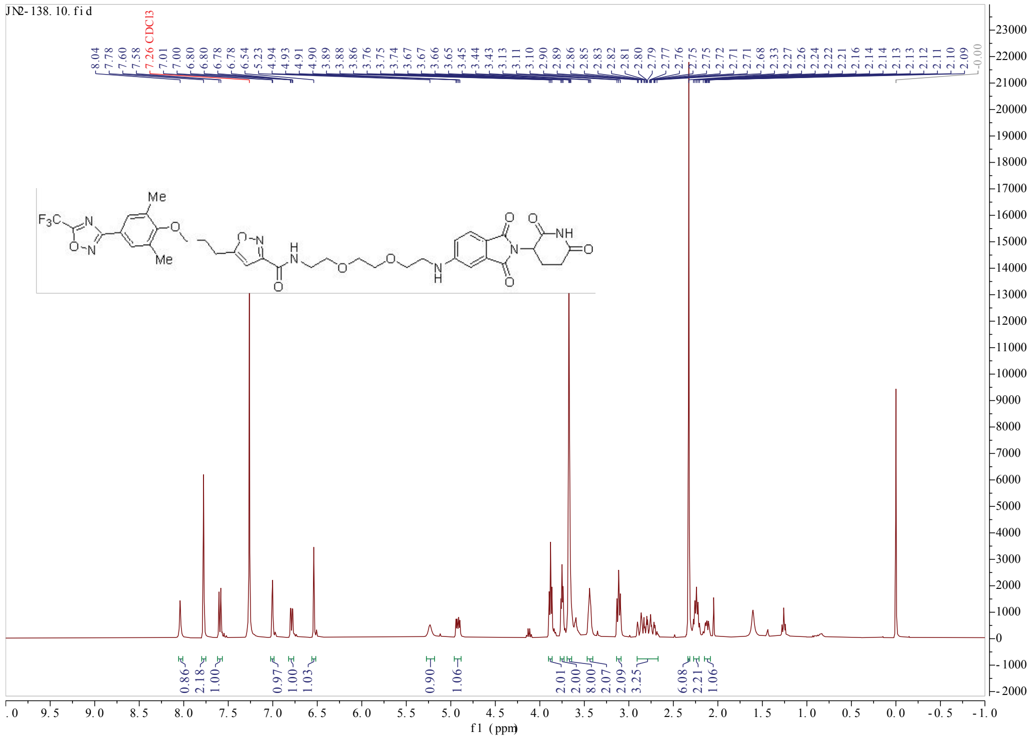

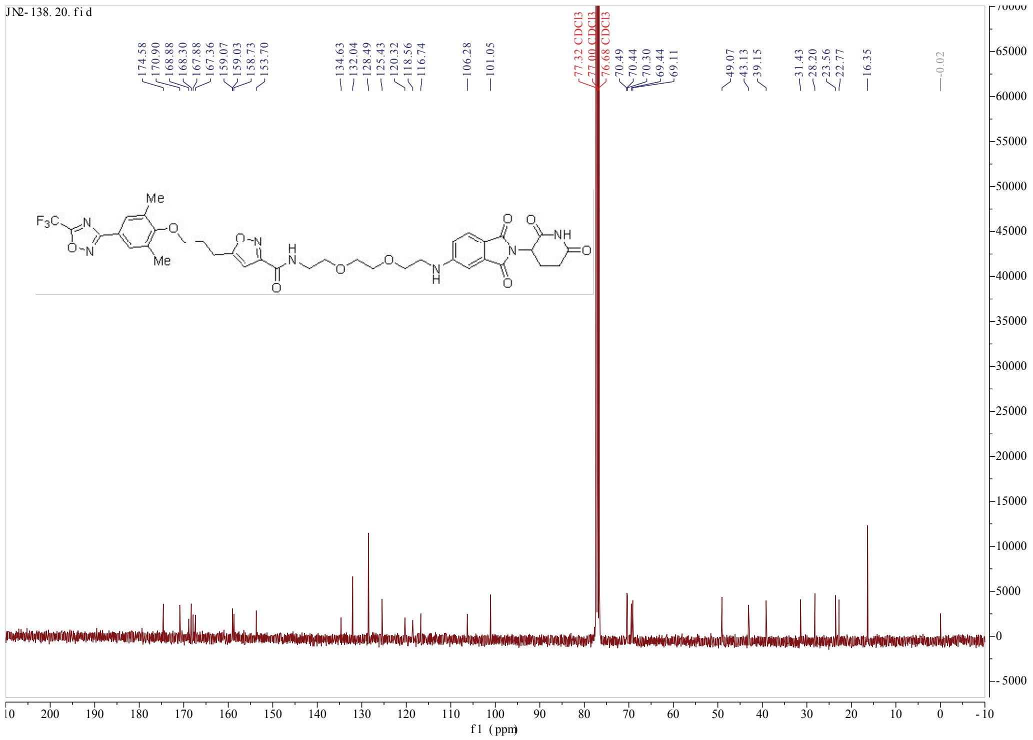

^{1}H NMR and ^{13}C NMR spectra of Jun15294

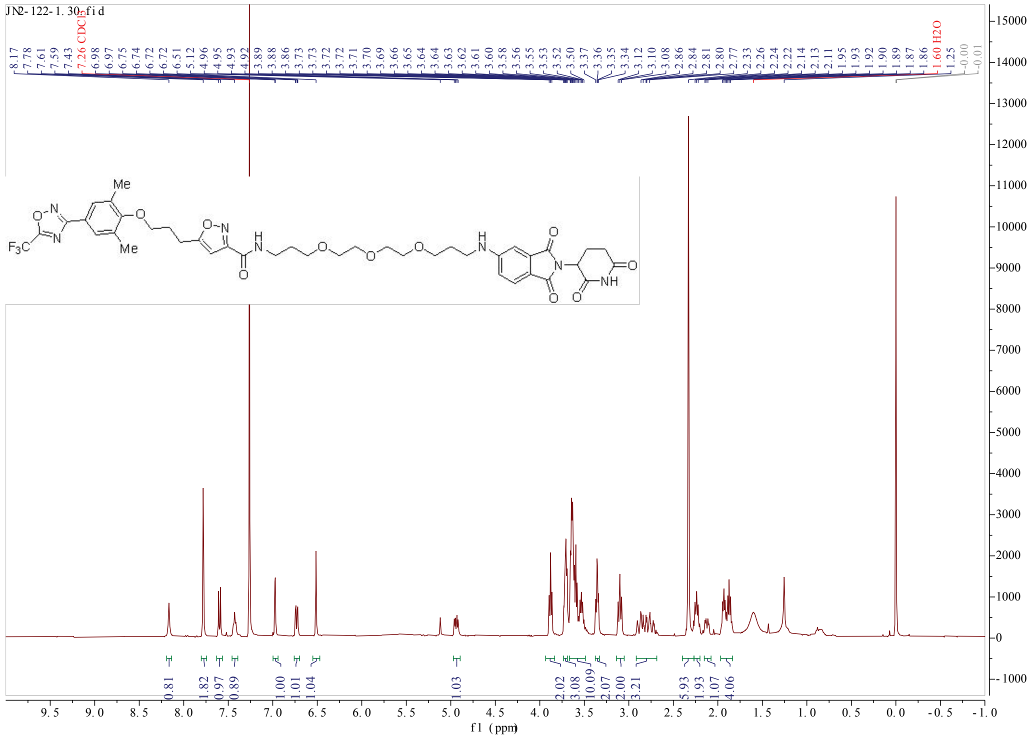

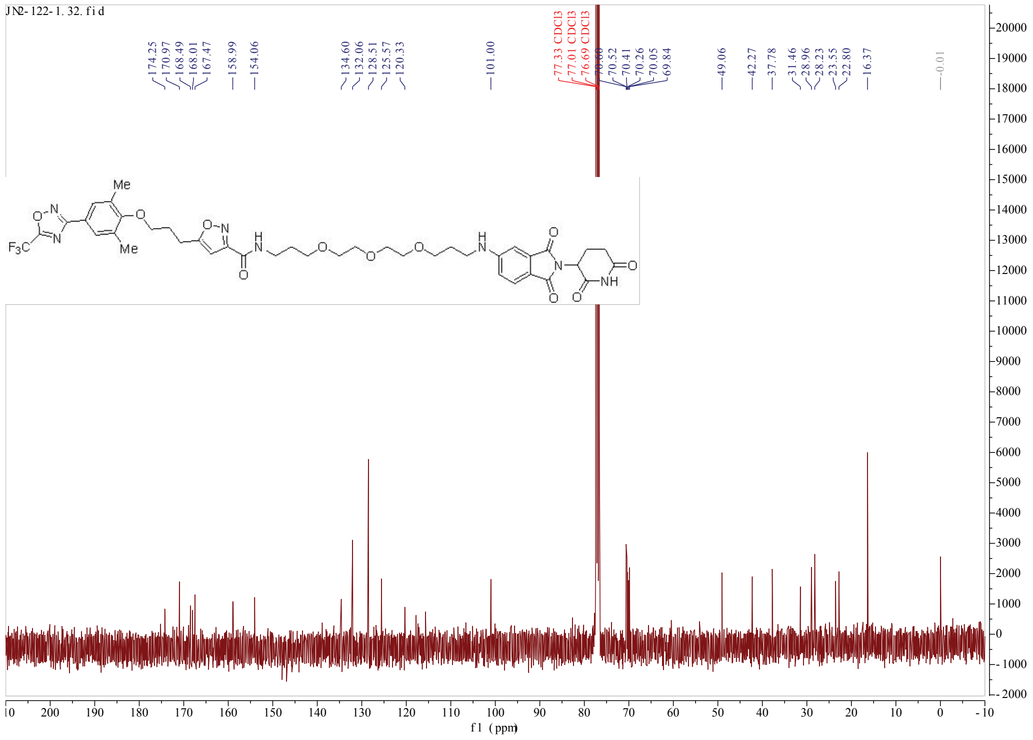

^{1}H NMR and ^{13}C NMR spectra of Jun15374

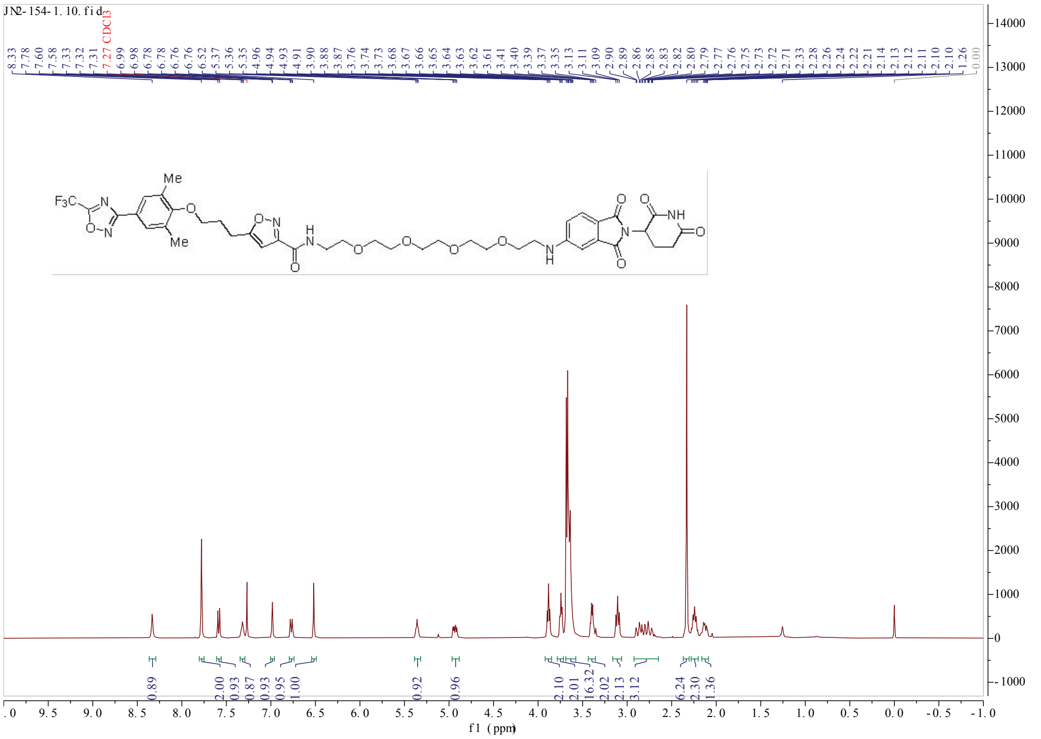

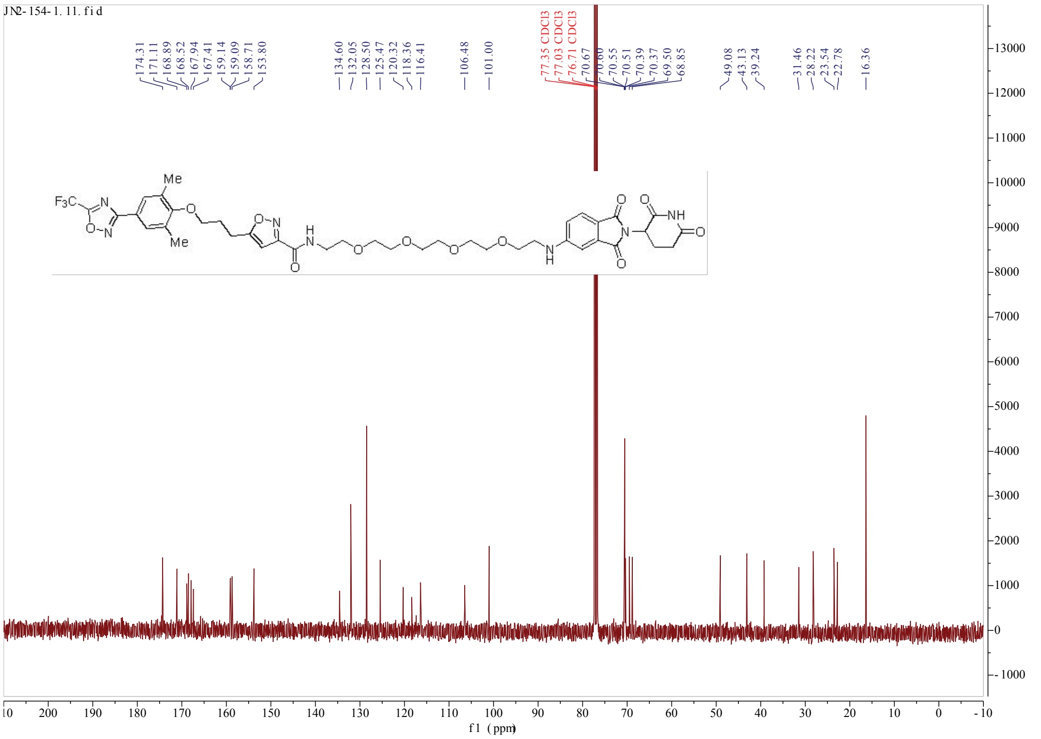

^{1}H NMR and ^{13}C NMR spectra of Jun15412

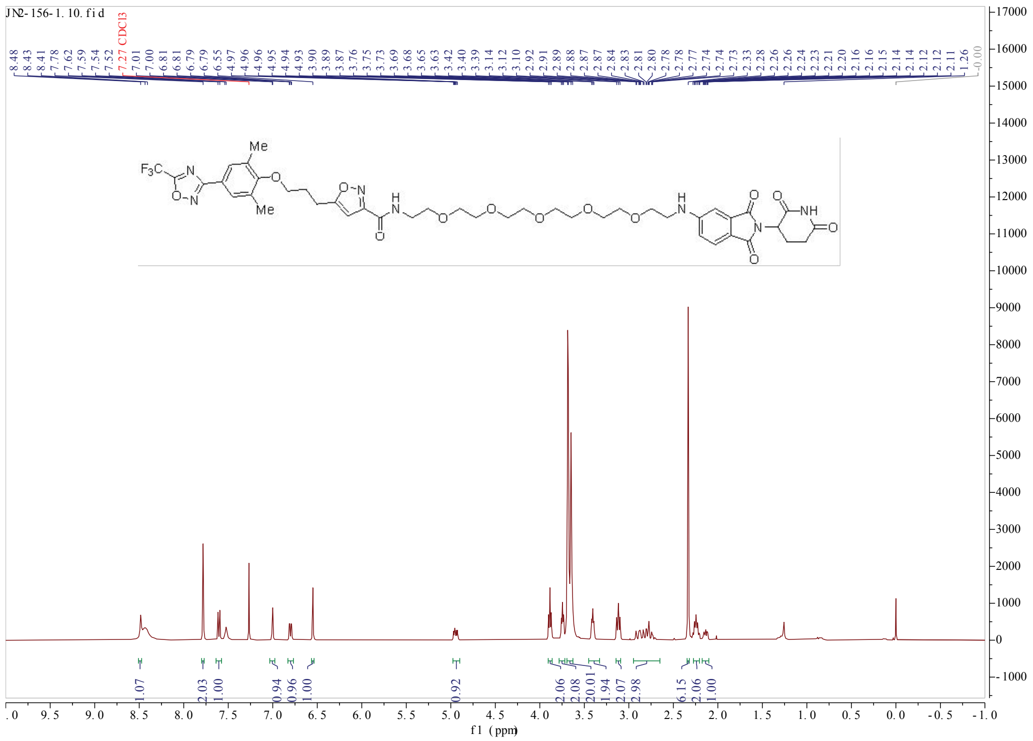

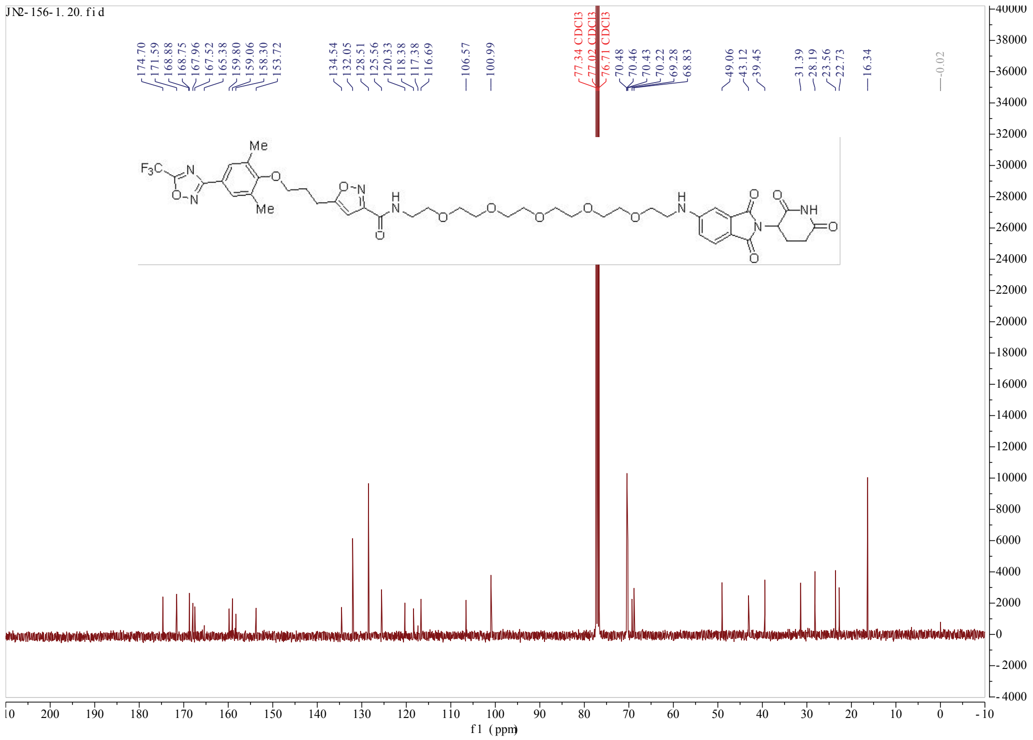

^{1}H NMR and ^{13}C NMR spectra of Jun15322

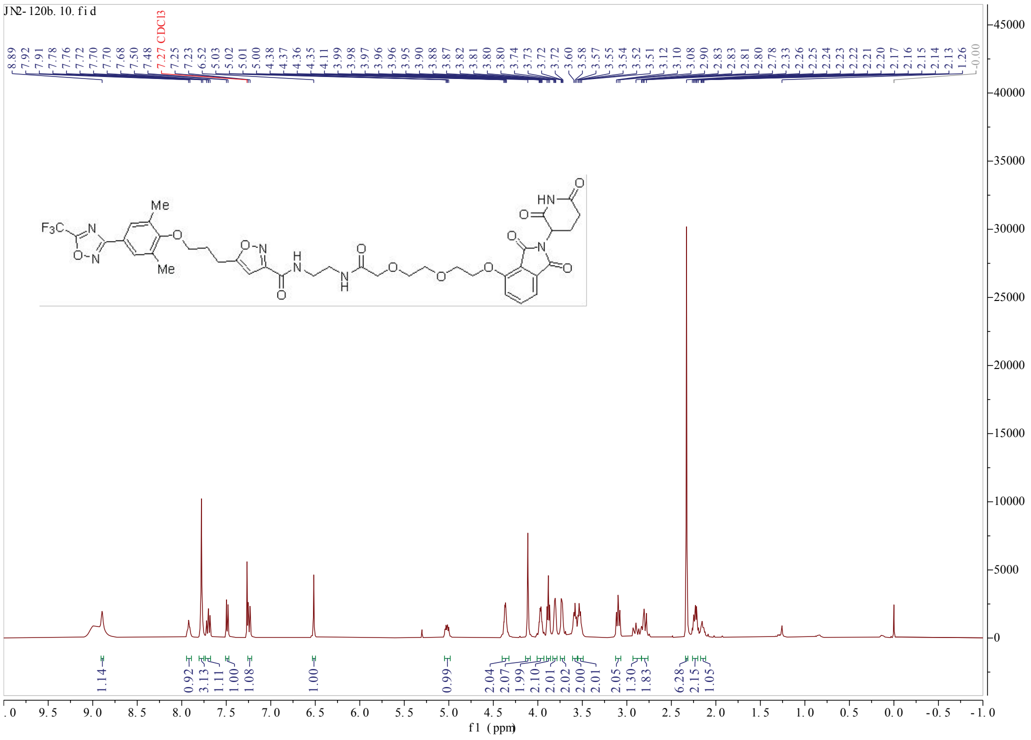

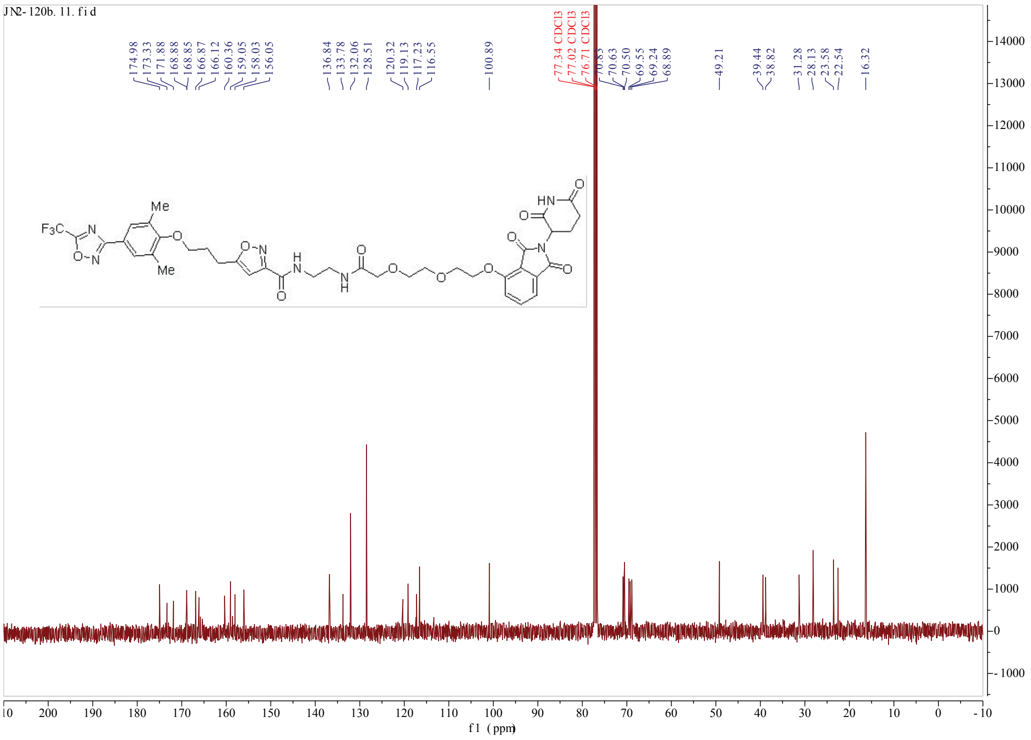

^{1}H NMR and ^{13}C NMR spectra of Jun15323

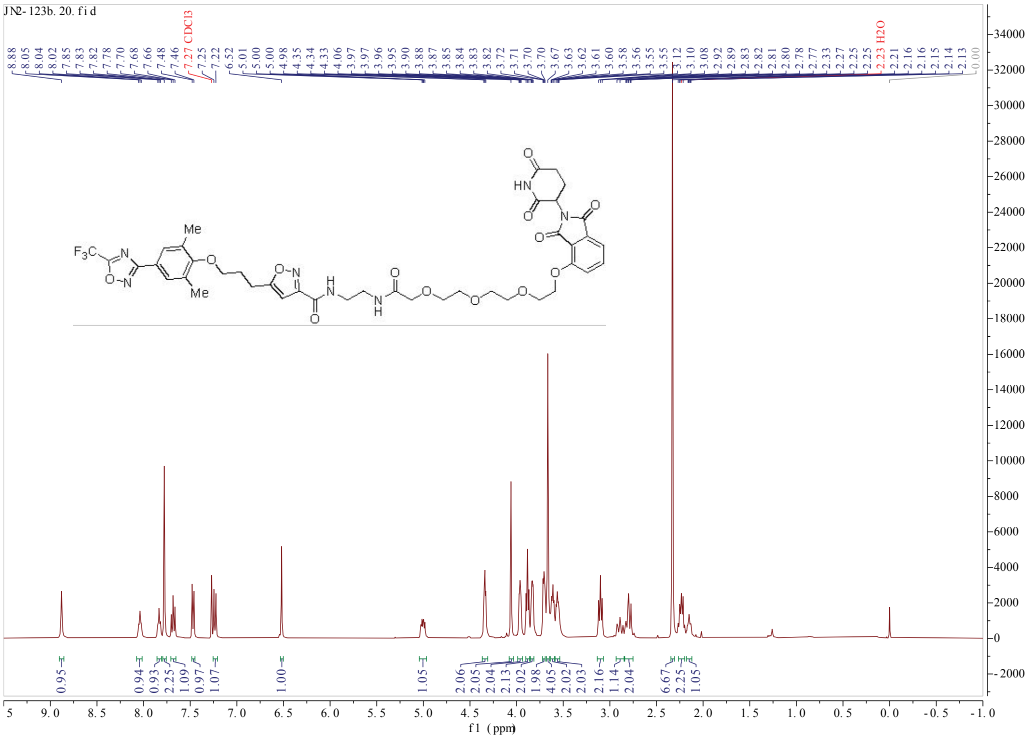

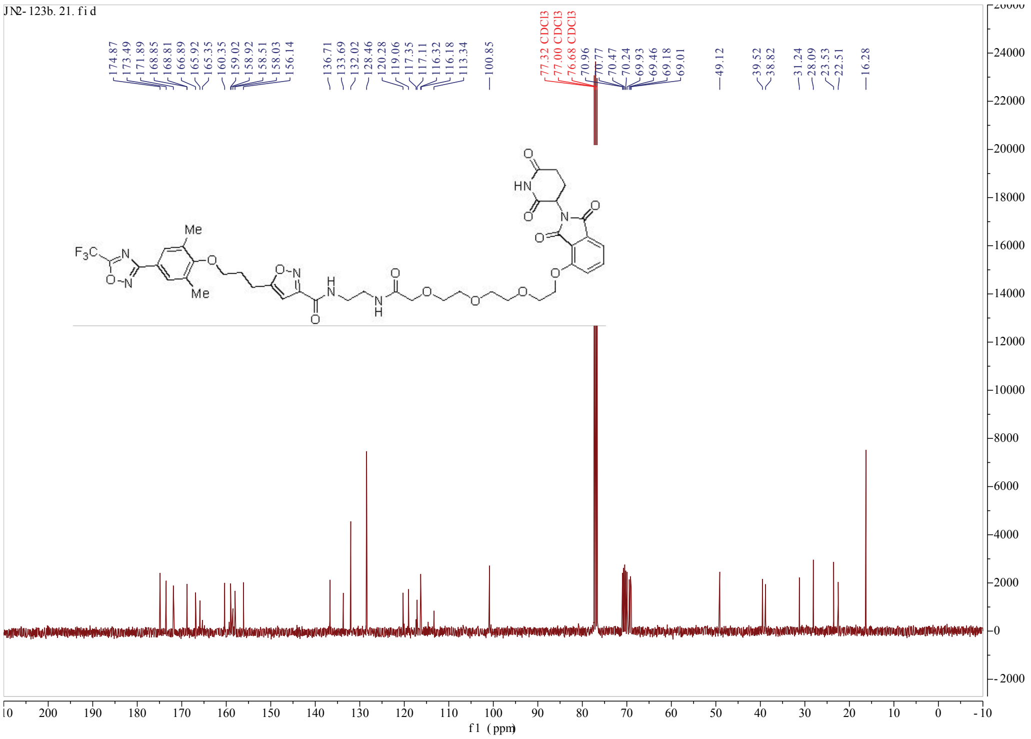

^{1}H NMR and ^{13}C NMR spectra of Jun15331

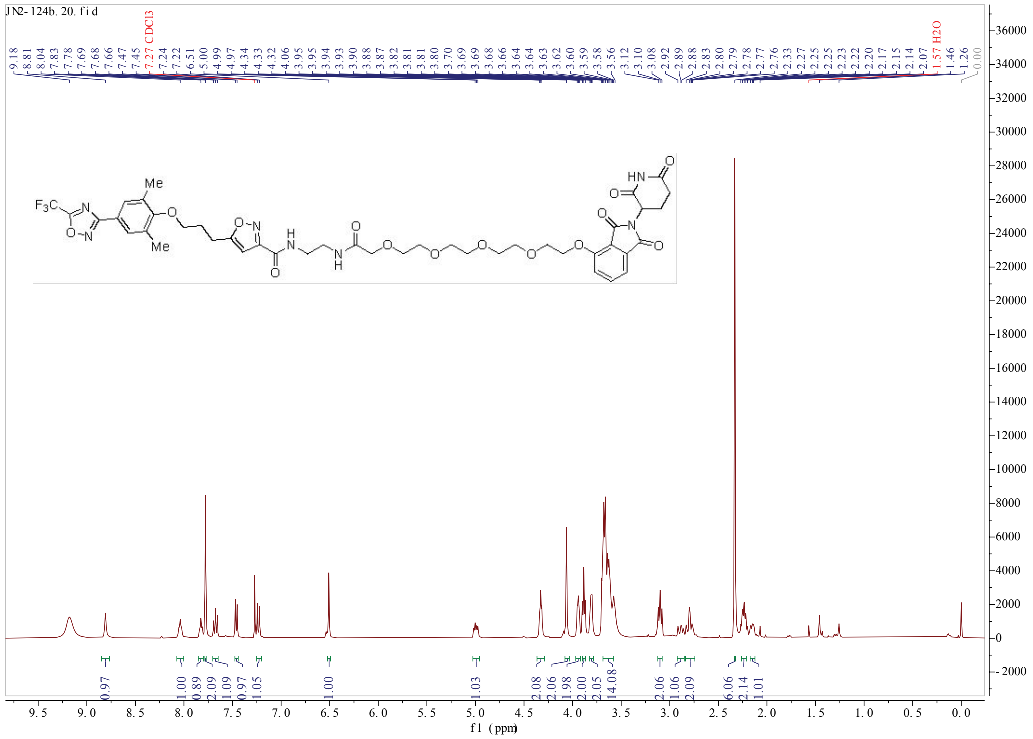

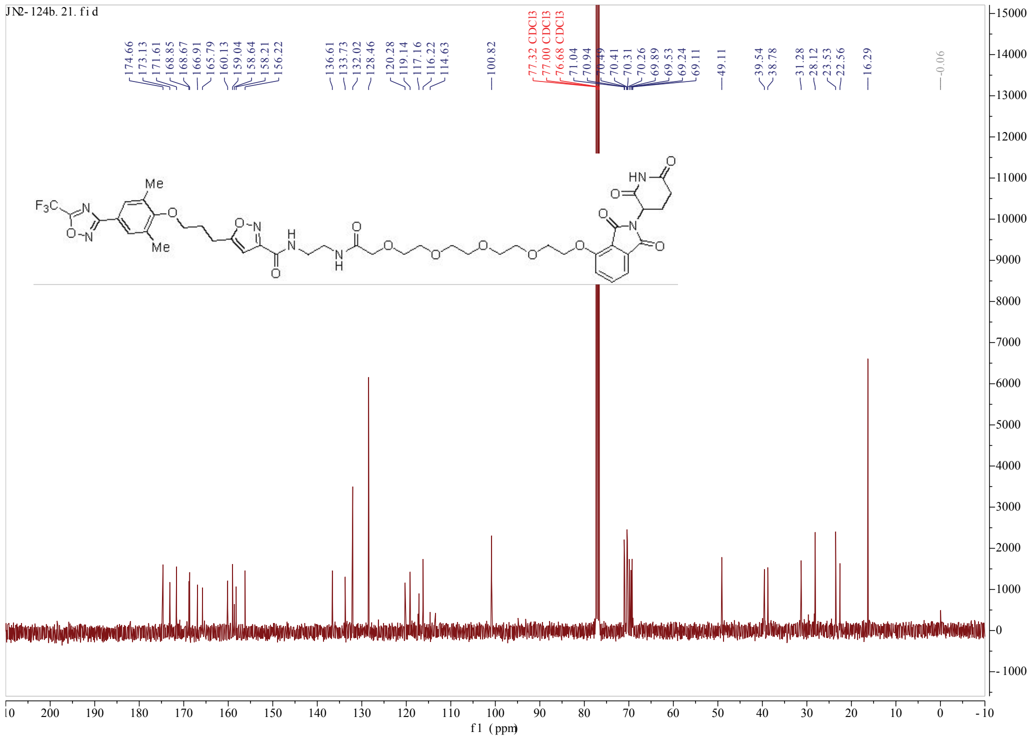

^{1}H NMR and ^{13}C NMR spectra of Jun15375

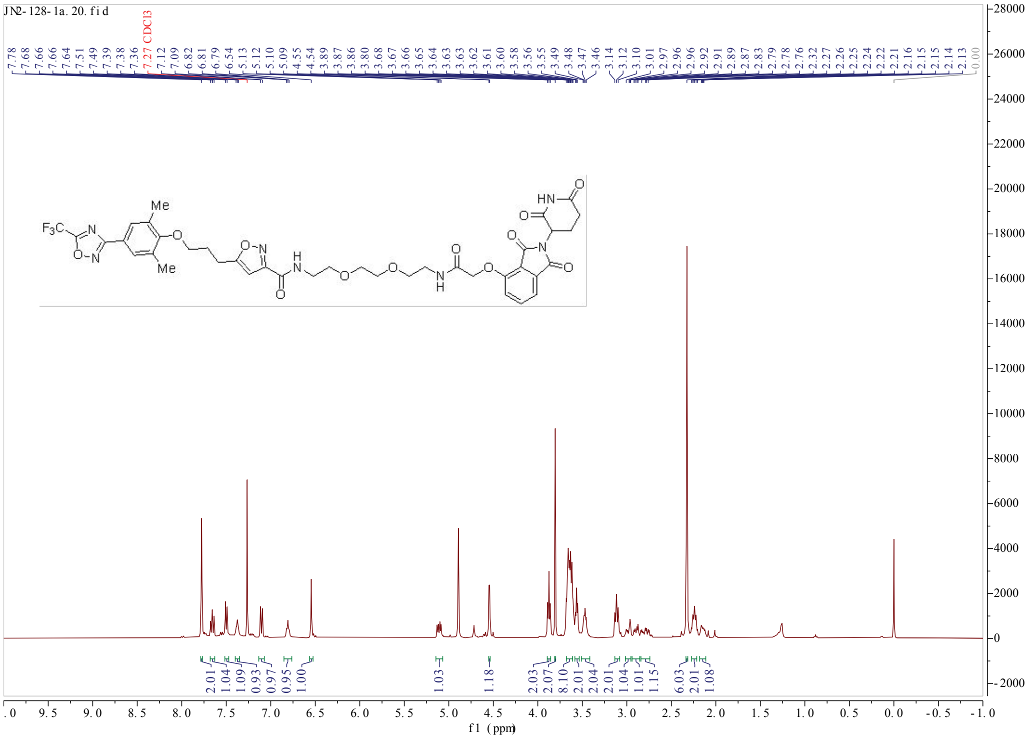

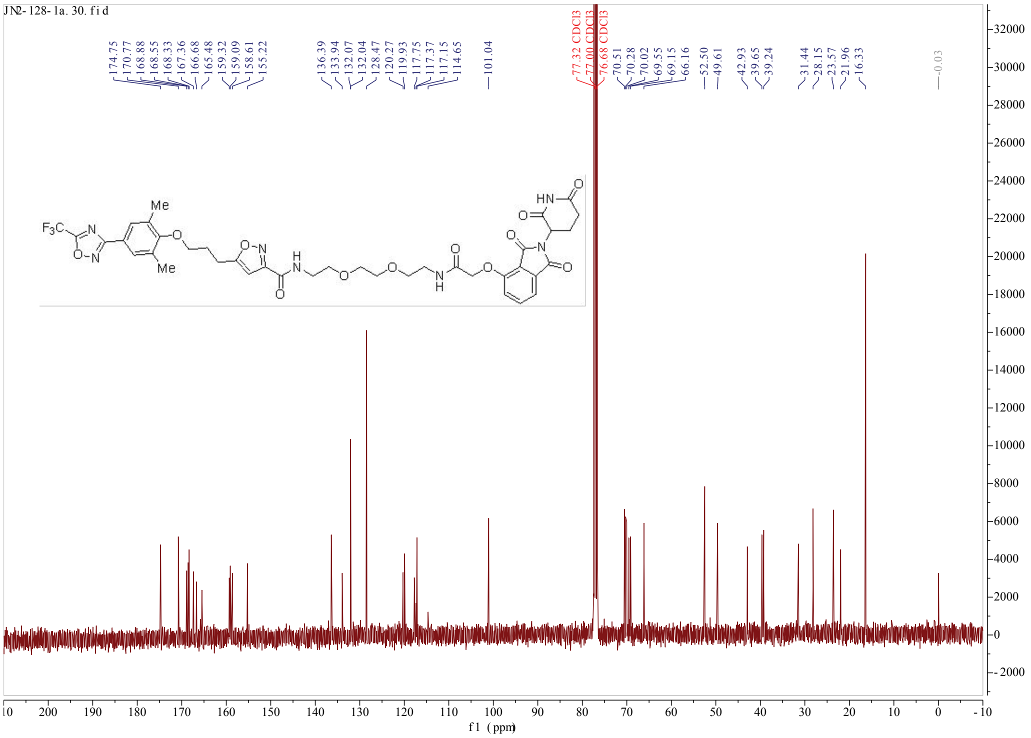

^{1}H NMR and ^{13}C NMR spectra of Jun15332

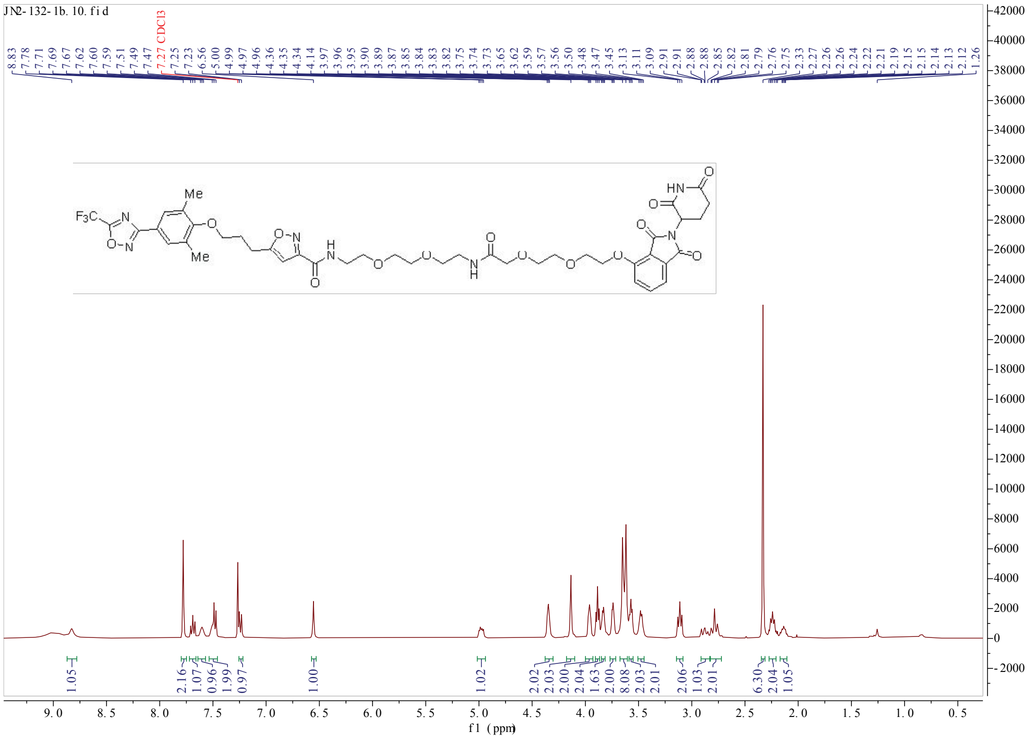

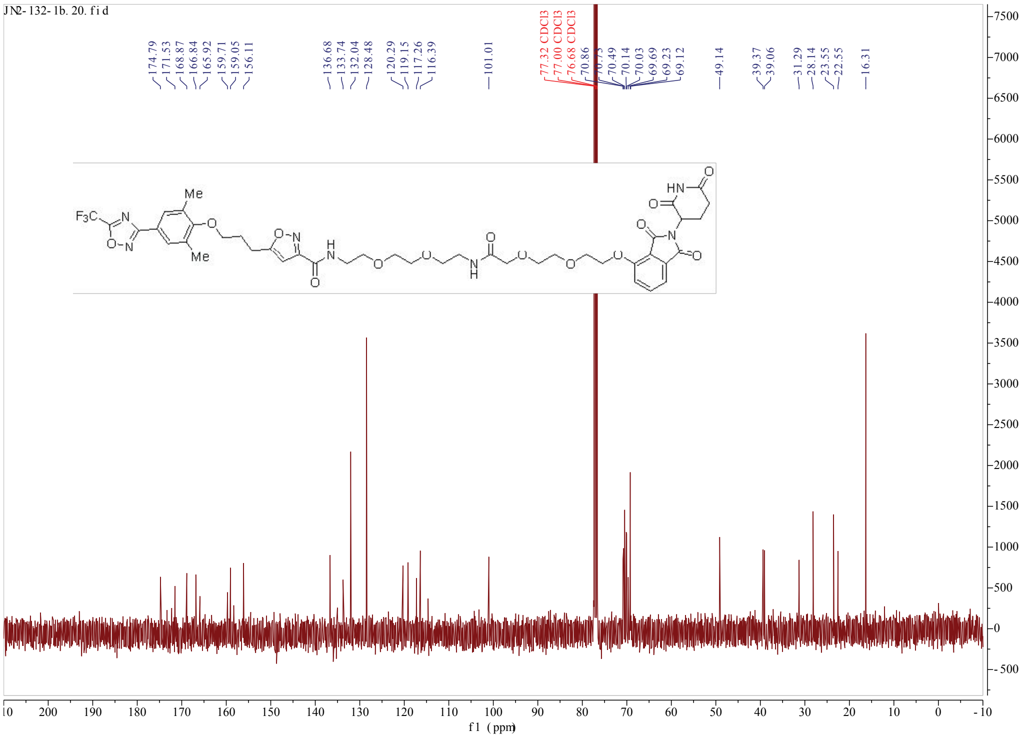

^{1}H NMR and ^{13}C NMR spectra of Jun15333

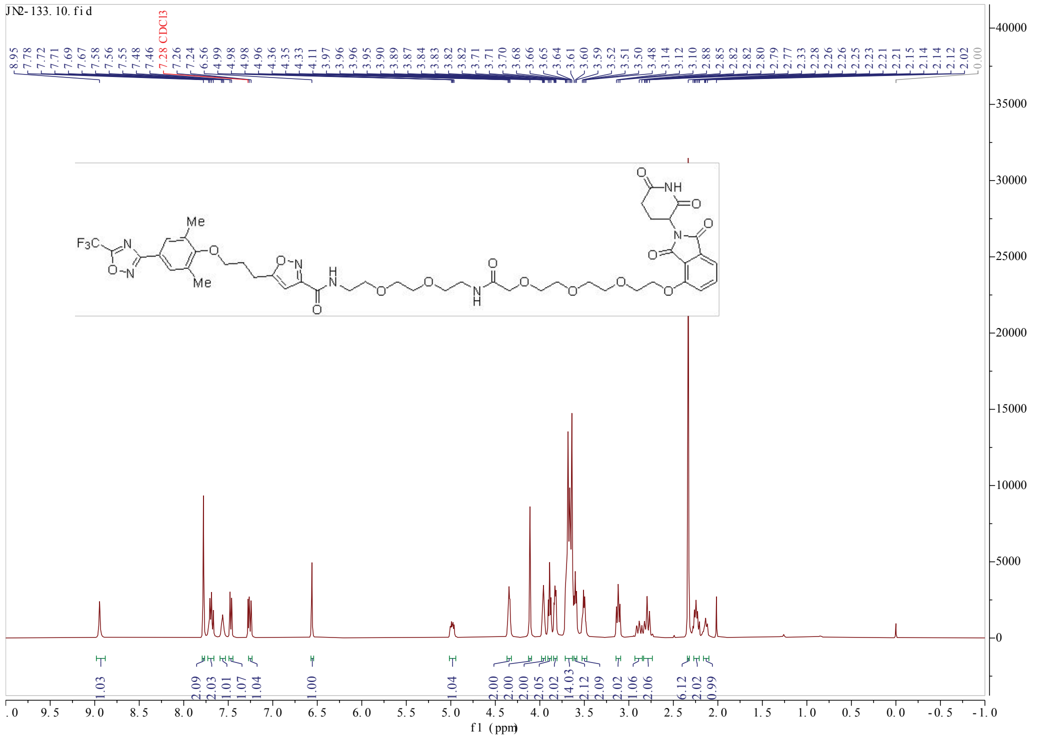

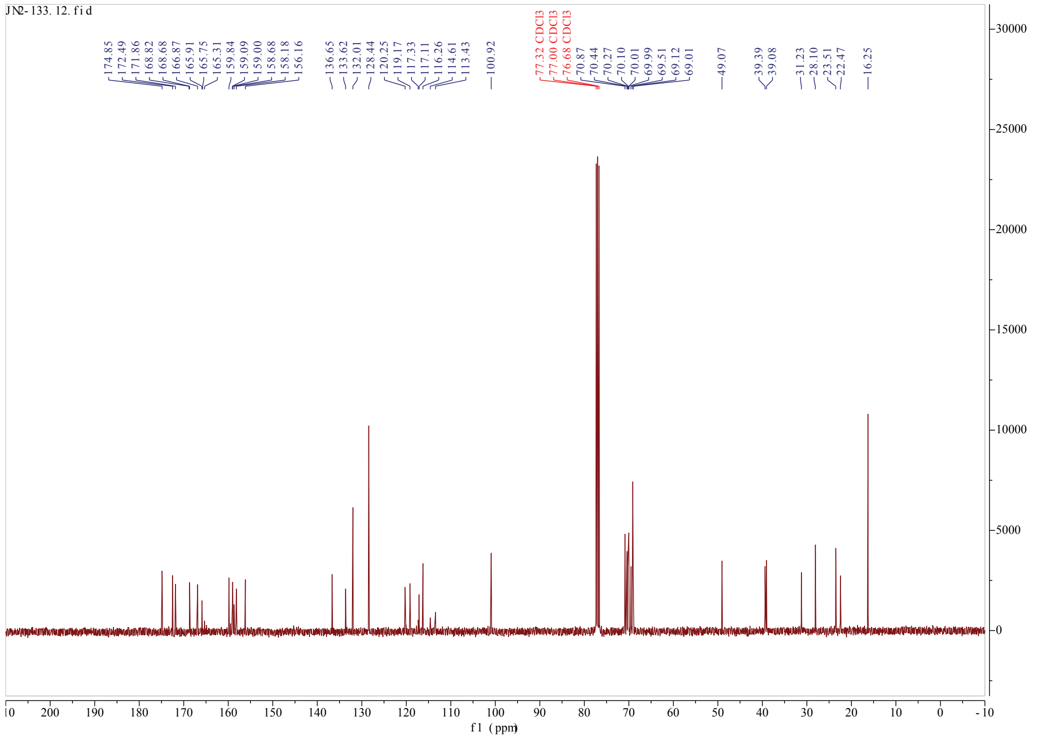

^{1}H NMR and ^{13}C NMR spectra of Jun15616

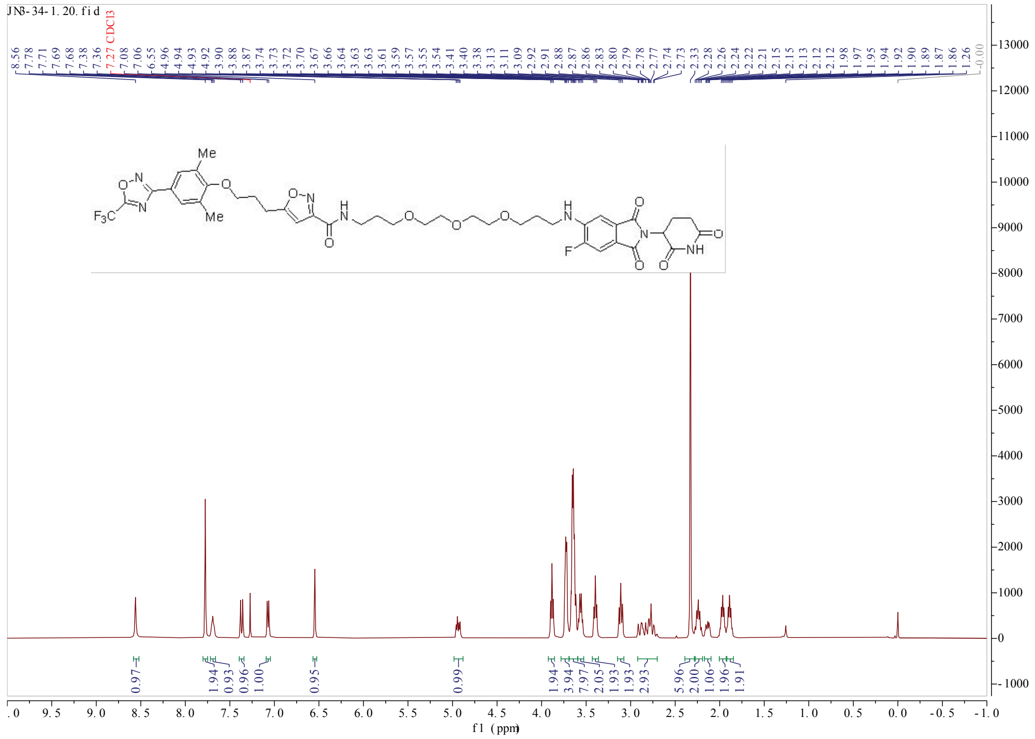

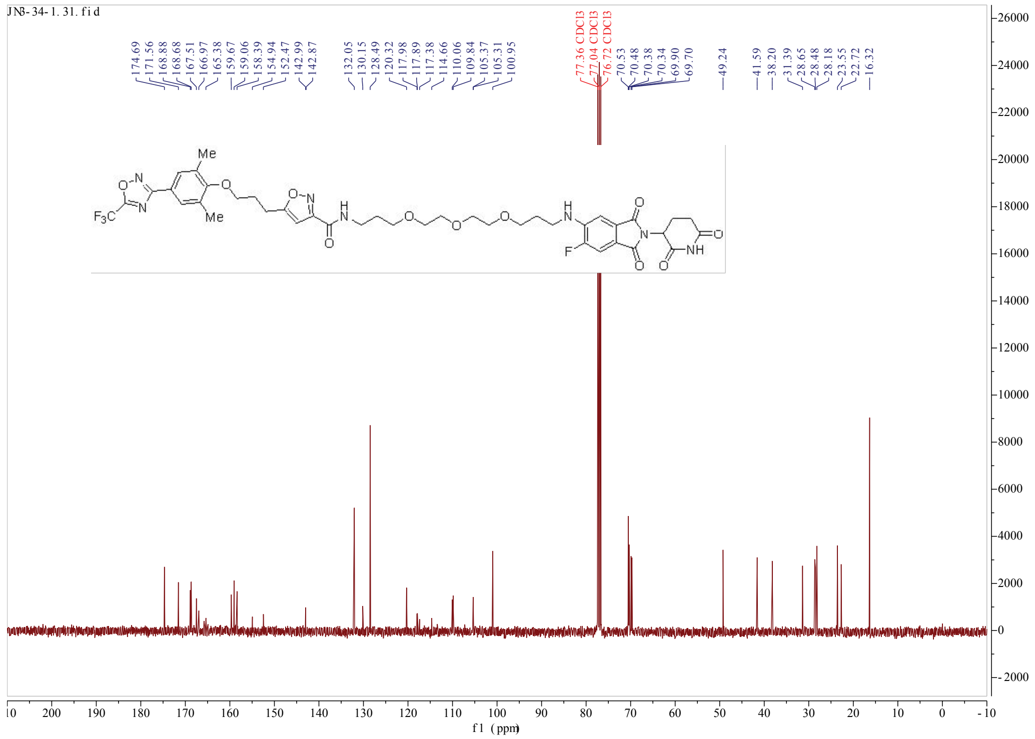

^{1}H NMR and ^{13}C NMR spectra of Jun15692

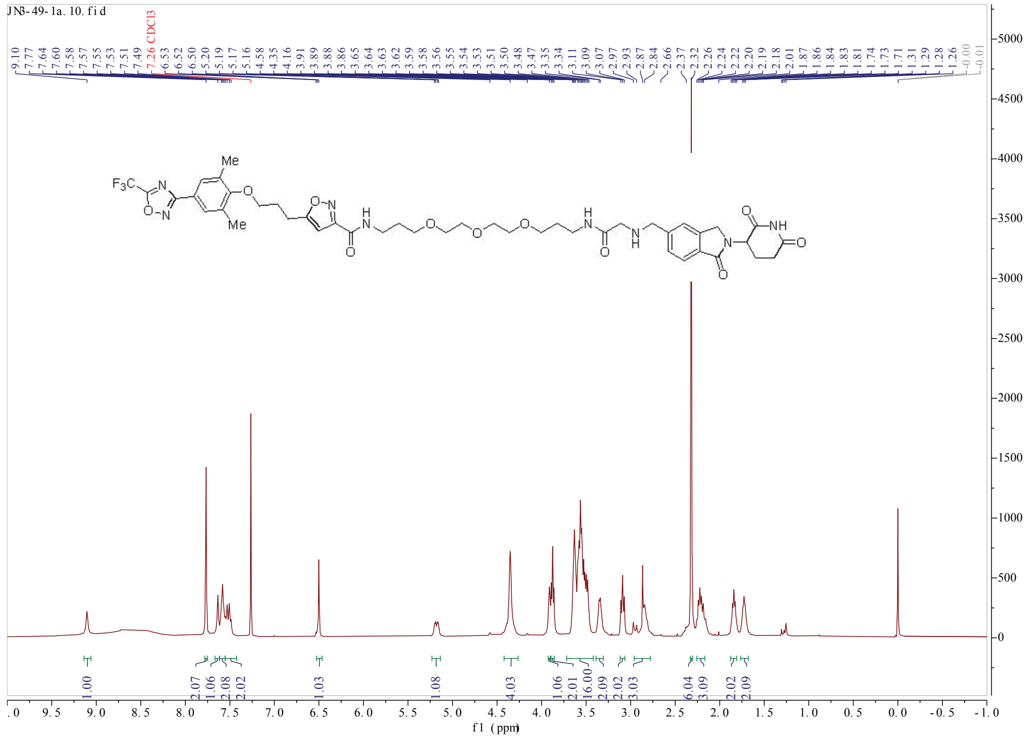

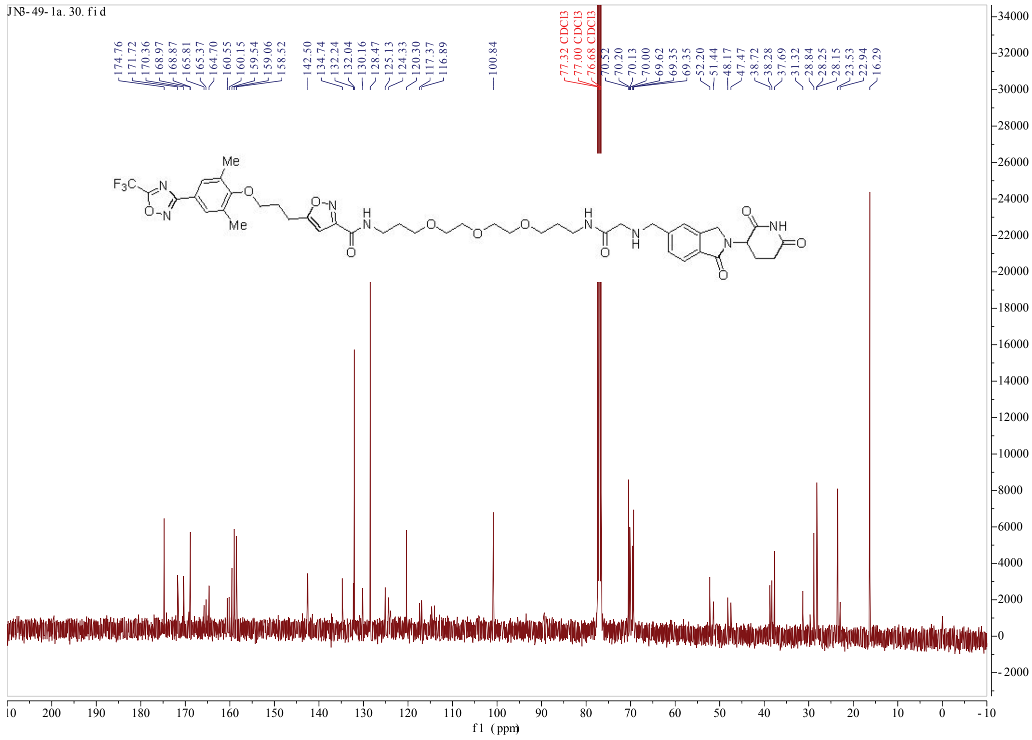

^{1}H NMR and ^{13}C NMR spectra of Jun15693

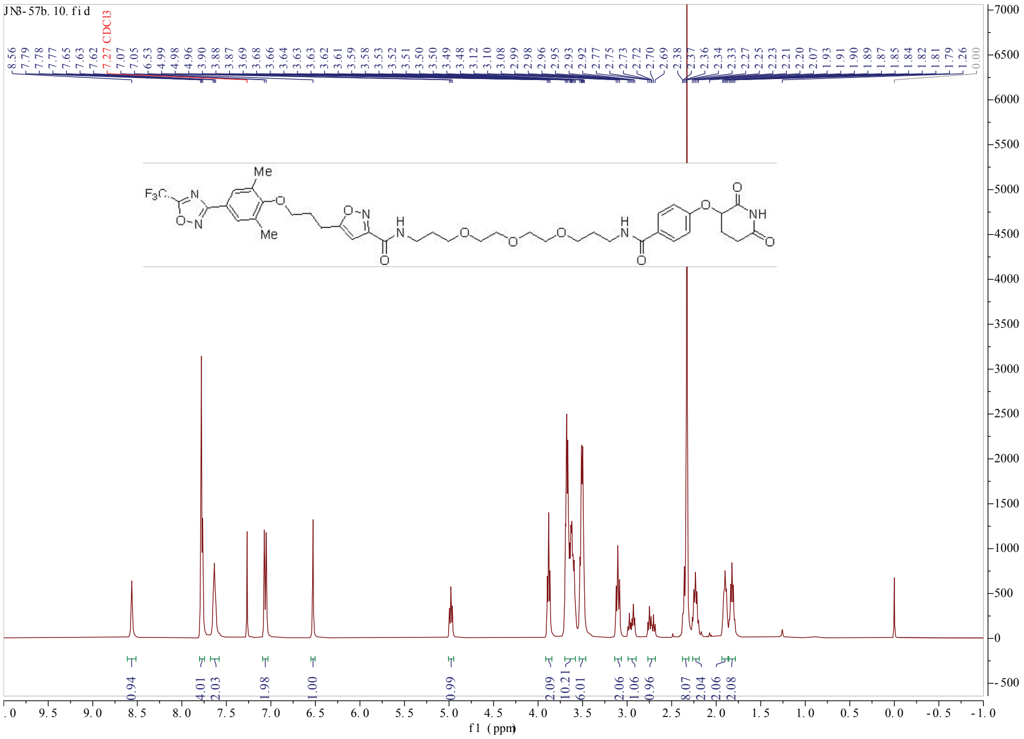

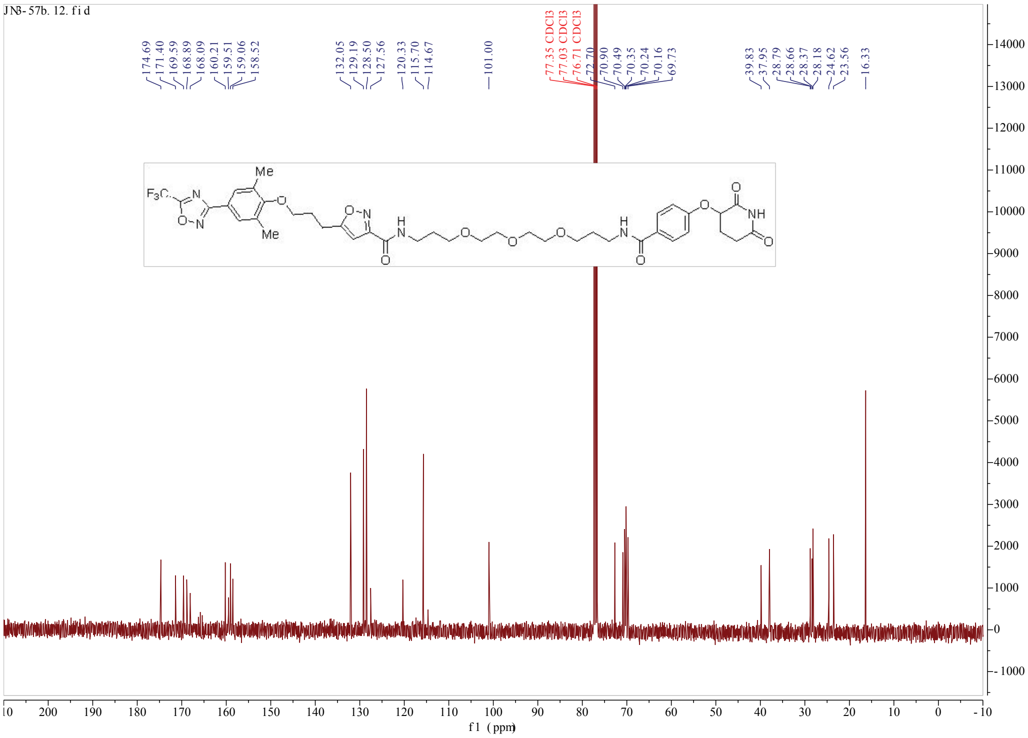

^{1}H NMR and ^{13}C NMR spectra of Jun15701

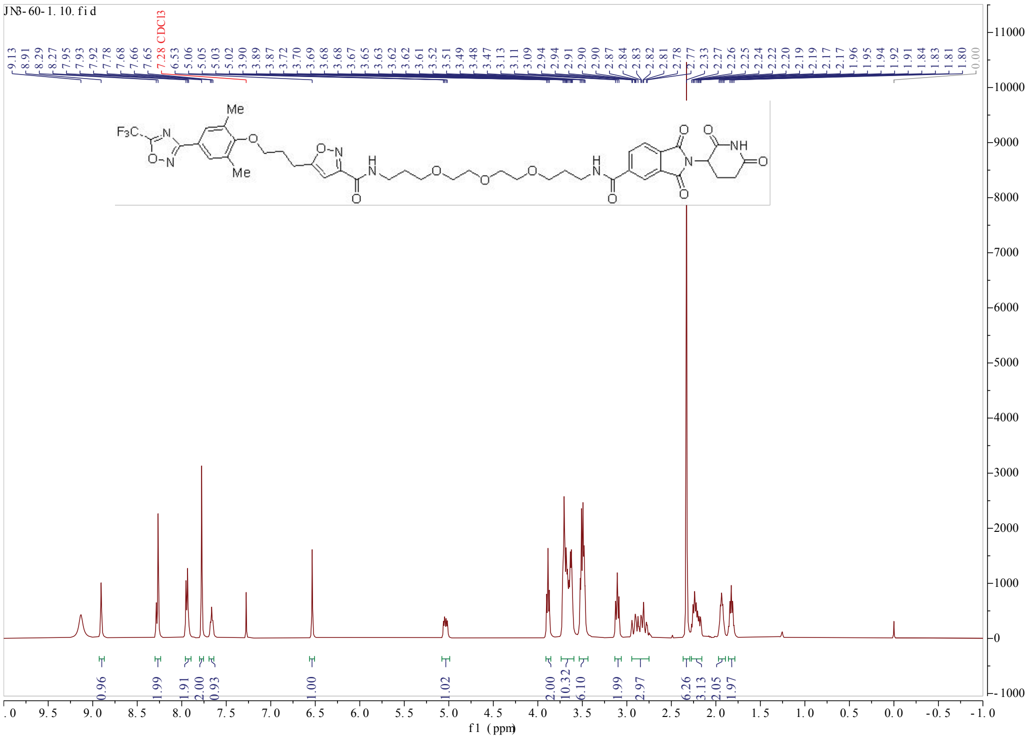

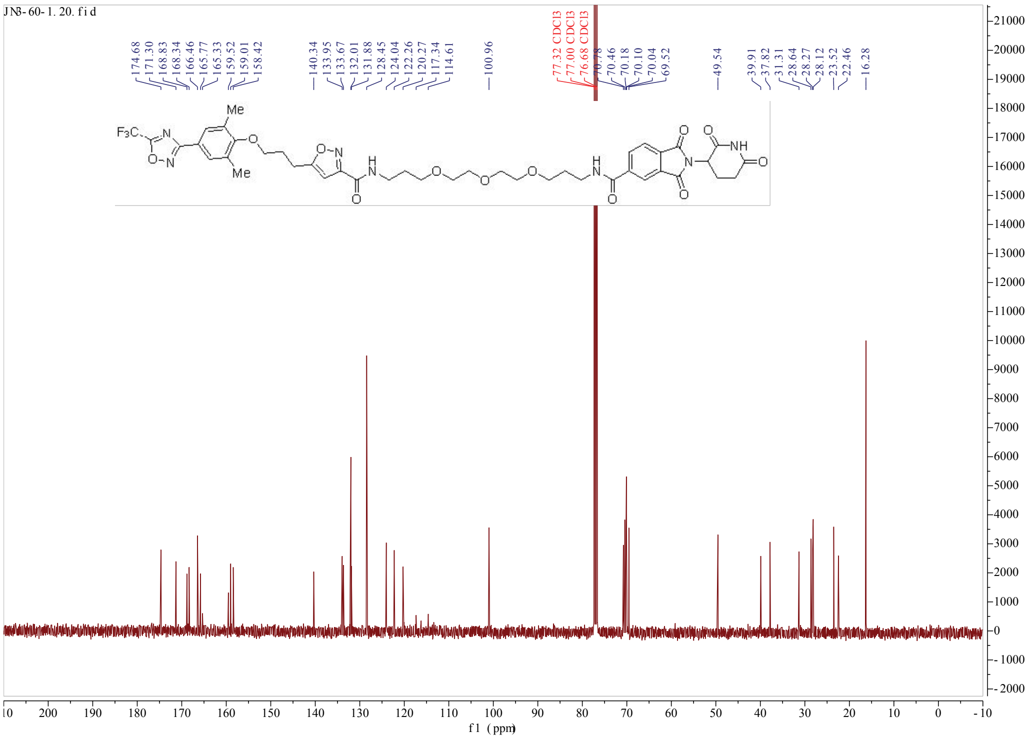

^{1}H NMR and ^{13}C NMR spectra of Jun15702

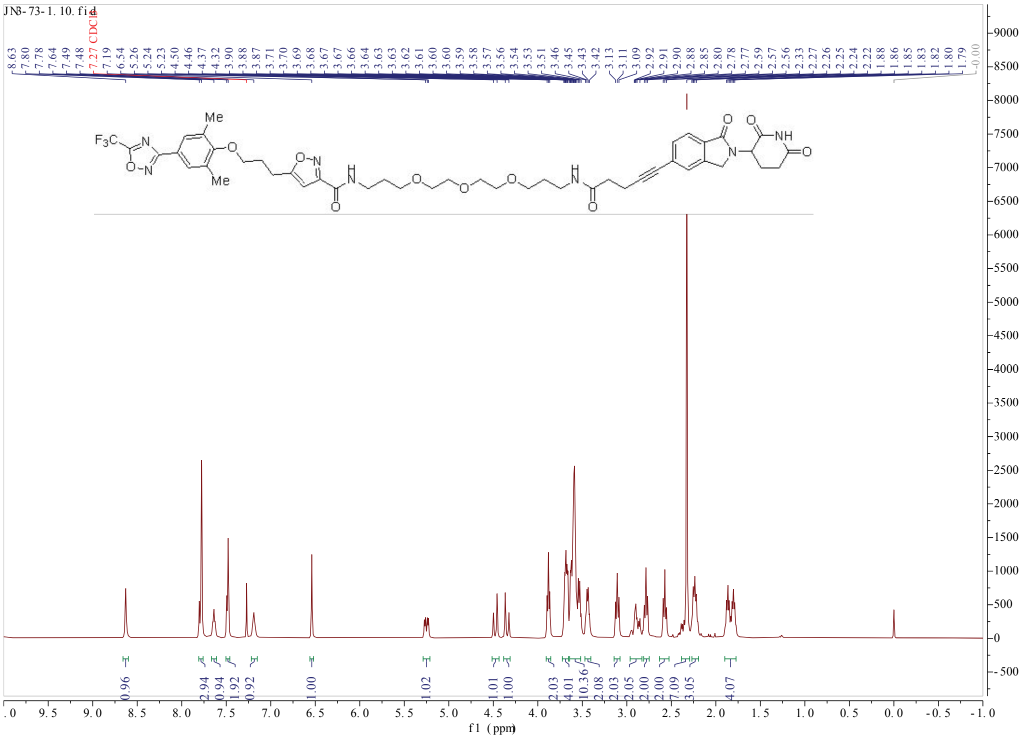

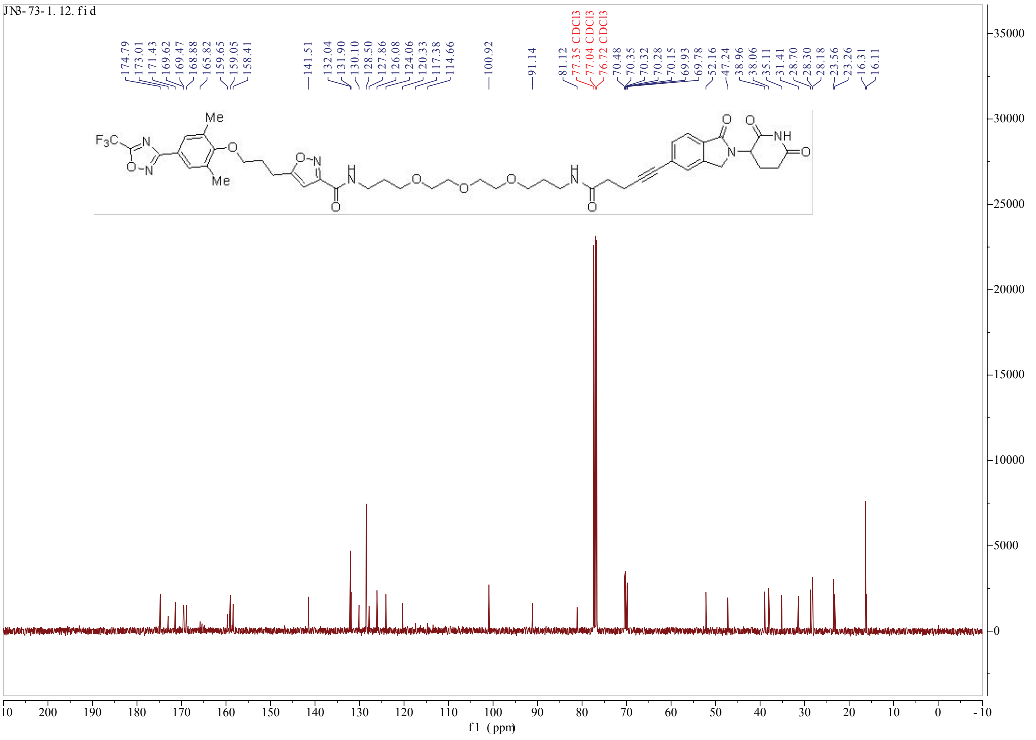

^{1}H NMR and ^{13}C NMR spectra of Jun15551

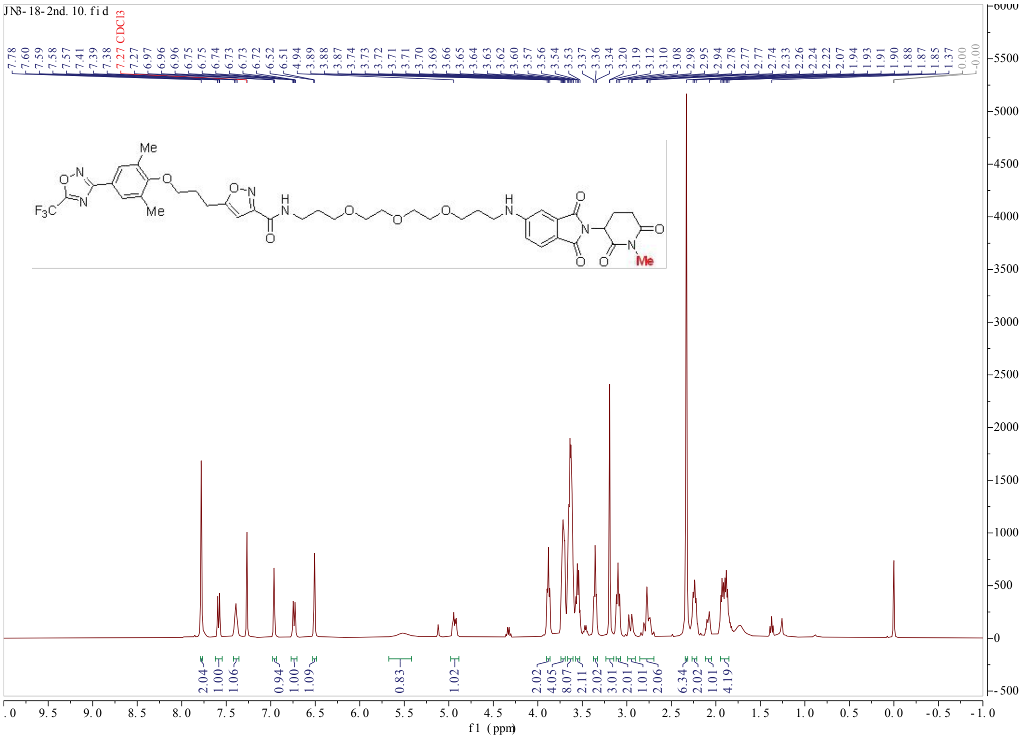

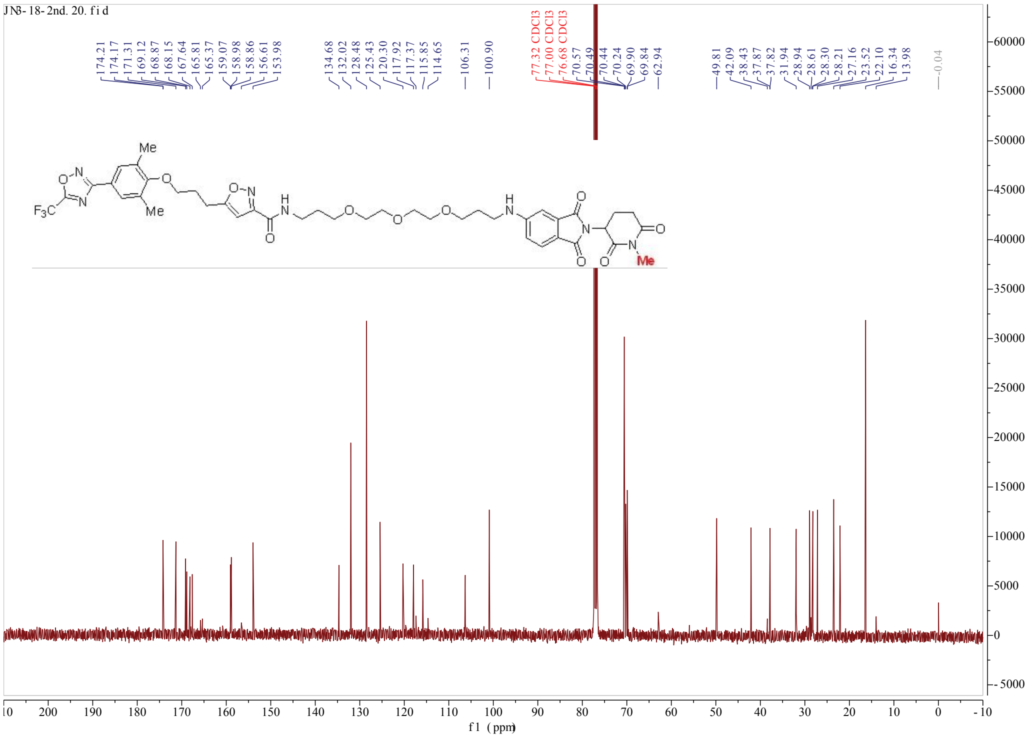

^{1}H NMR and ^{13}C NMR spectra of Jun15953

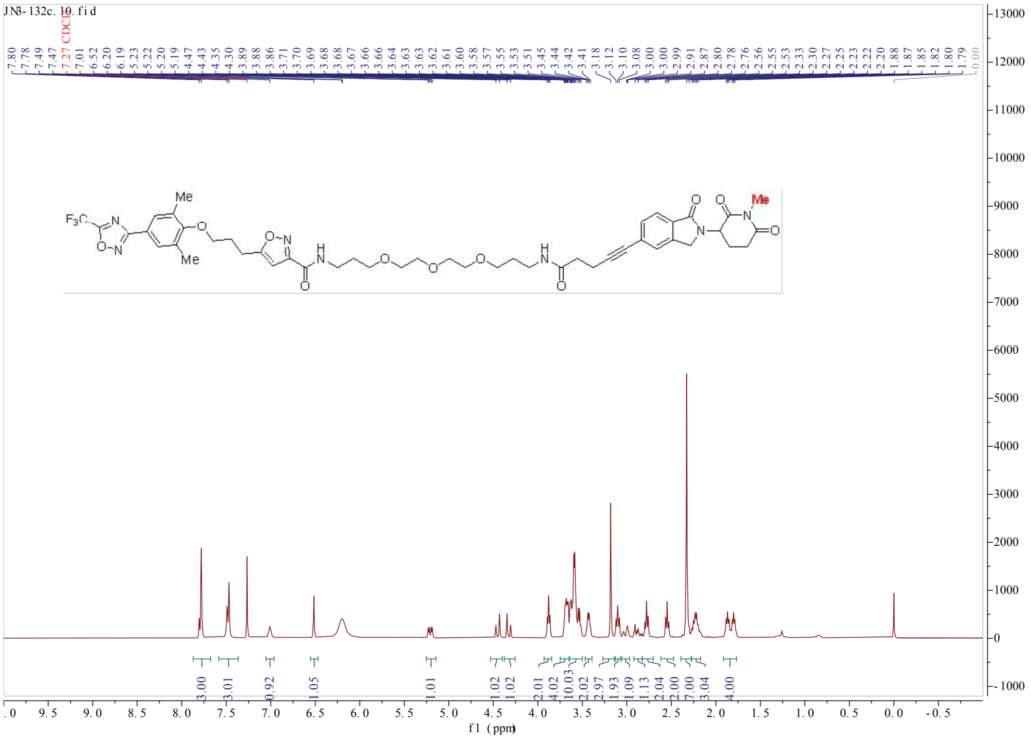

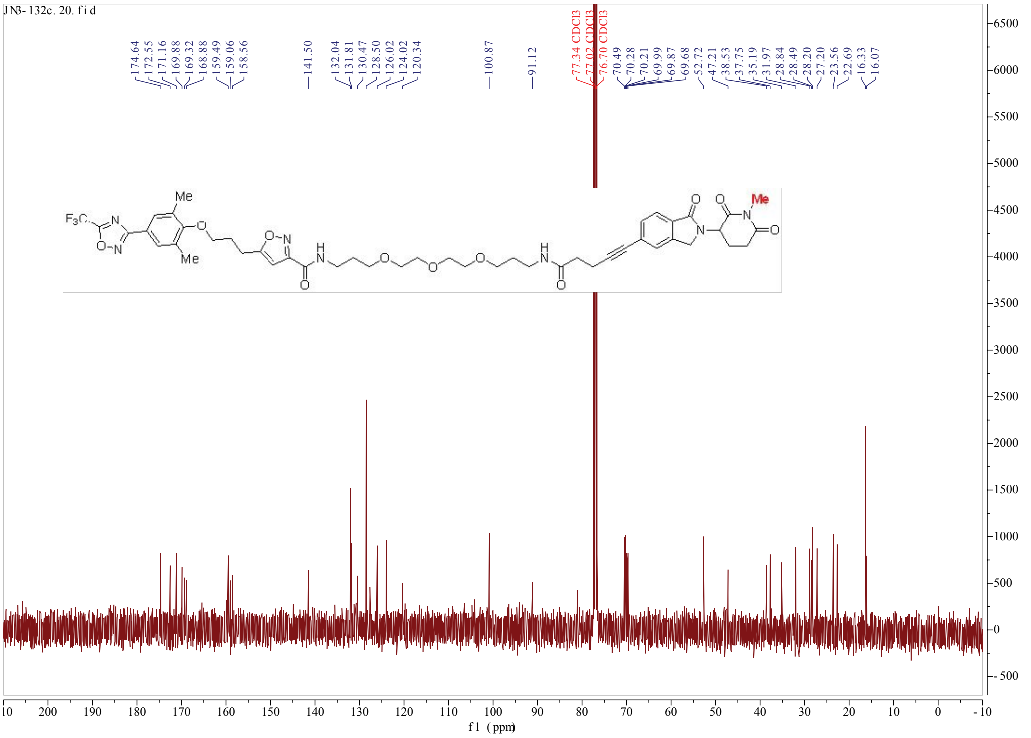

HPLC trace of Jun1522

Single Injection Report

**Data file:**	D:\CDSProjects\Checkout and Familiarization\Results\KL\Jun1522_HPLC_2.sirslt\Jun1522_HPLC_2.dx		
**Sample name:**	Jun1522_HPLC_2		
**Description:**			
**Sample amount:**	0.000	**Sample type:**	Sample
**Instrument:**	LCMS 1	**Location:**	P2-D1
**Injection date:**	2025-12-29 11:00:12-05:00	**Injection:**	1 of 1
**Acq. method:**	VP1 PROTAC_HPLC__method_10 min.amx	**Injection volume:**	5.000 µL
**Analysis method:**	3D UV Quantitative_DefaultMethod.pmx	**Acq. operator:**	SYSTEM
**Last changed:**	2023-03-22 11:19:18-04:00		

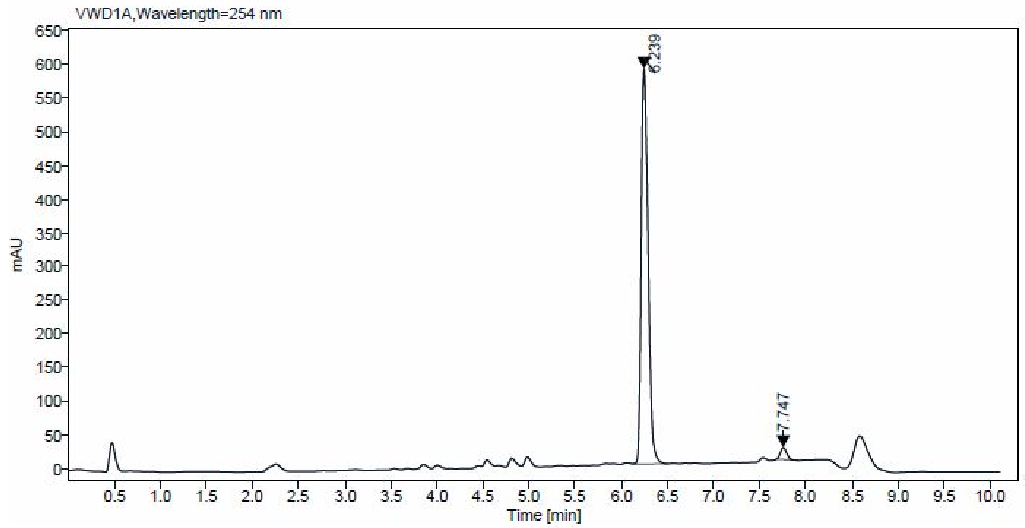

Signal: VWD1A,Wavelength=254 nm

RT [min]	Name	Area	Area%	Max Peak%	Height	Type	Width (50%)
7.747		81.6143	2.6476	2.720	17.800	MM m	
6.239		3000.9076	97.3524	100.000	585.395	VB	

HPLC trace of Jun1554

Single Injection Report

**Data file:**	D:\CDSProjects\Checkout and Familiarization\Results\KL\Jun1554_HPLC.sirslt\Jun1554_HPLC.dx		
**Sample name:**	Jun1554_HPLC		
**Description:**			
**Sample amount:**	0.000	**Sample type:**	Sample
**Instrument:**	LCMS 1	**Location:**	P2-D3
**Injection date:**	2025-12-29 11:22:10-05:00	**Injection:**	1 of 1
**Acq. method:**	VP1 PROTAC_HPLC__method_10 min.amx	**Injection volume:**	5.000 µL
**Analysis method:**	3D UV Quantitative_DefaultMethod.pmx	**Acq. operator:**	SYSTEM
**Last changed:**	2023-03-22 11:19:18-04:00		

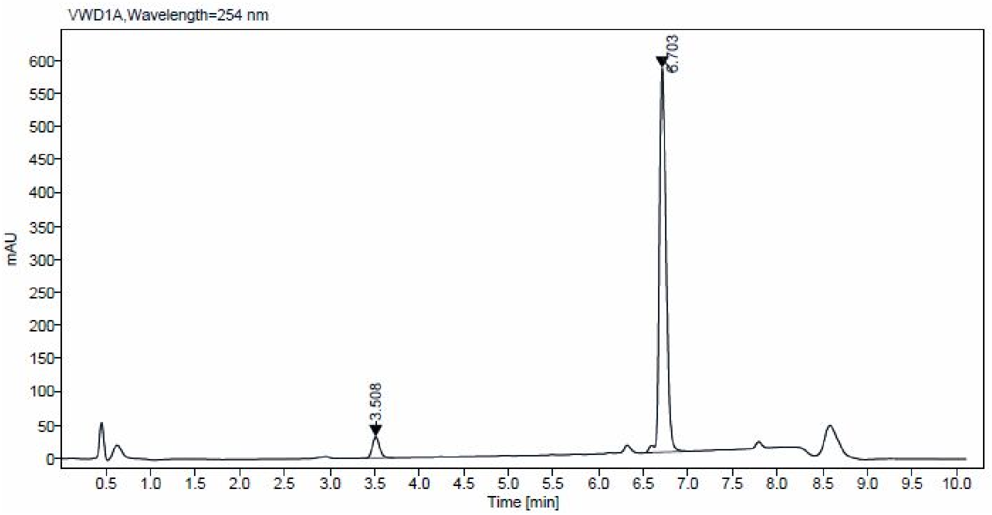

Signal: VWD1A,Wavelength=254 nm

RT [min]	Name	Area	Area%	Max Peak%	Height	Type	Width (50%)
3.508		176.5495	5.5462	5.872	31.907	BB	
6.703		3006.7145	94.4538	100.000	579.208	MB m	
	**Sum**	**3183.2641**					

HPLC trace of Jun14956

Single Injection Report

Agilent

**Data file:**	D:\CDSProjects\Checkout and Familiarization\Results\KL\Jun14956_HPLC.sirslt\Jun14956_HPLC.dx		
**Sample name:**	Jun14956_HPLC		
**Description:**			
**Sample amount:**	0.000	**Sample type:**	Sample
**Instrument:**	LCMS 1	**Location:**	P2-D2
**Injection date:**	2025-12-29 11:11:12-05:00	**Injection:**	1 of 1
**Acq. method:**	VP1 PROTAC_HPLC__method_10 min.amx	**Injection volume:**	5.000 µL
**Analysis method:**	3D UV Quantitative_DefaultMethod.pmx	**Acq. operator:**	SYSTEM
**Last changed:**	2023-03-22 11:19:18-04:00		

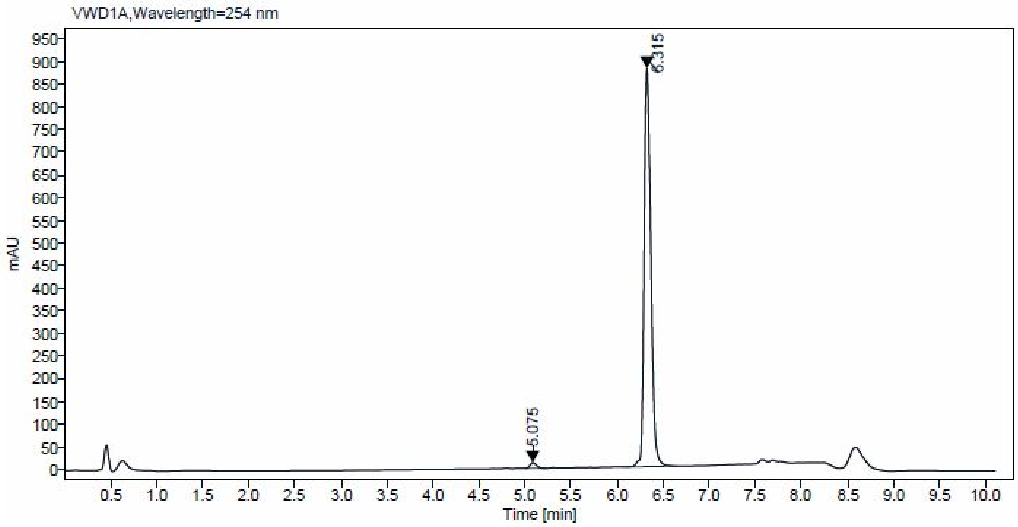

Signal: VWD1A,Wavelength=254 nm

RT [min]	Name	Area	Area%	Max Peak%	Height	Type	Width (50%)
5.075		59.0215	1.3006	1.318	12.363	VB	
6.315		4478.9398	98.6994	100.000	877.933	BB	
	**Sum**	**4537.9613**					

HPLC trace of Jun15183

Single Injection Report

**Data file:**	D:\CDSProjects\Checkout and Familiarization\Results\KL\Jun15183_HPLC.sirslt\Jun15183_HPLC.dx		
**Sample name:**	Jun15183_HPLC		
**Description:**			
**Sample amount:**	0.000	**Sample type:**	Sample
**Instrument:**	LCMS 1	**Location:**	P2-E3
**Injection date:**	2025-12-29 13:22:47-05:00	**Injection:**	1 of 1
**Acq. method:**	VP1 PROTAC_HPLC__method_10 min.amx	**Injection volume:**	5.000 µL
**Analysis method:**	3D UV Quantitative_DefaultMethod.pmx	**Acq. operator:**	SYSTEM
**Last changed:**	2023-03-22 11:19:18-04:00		

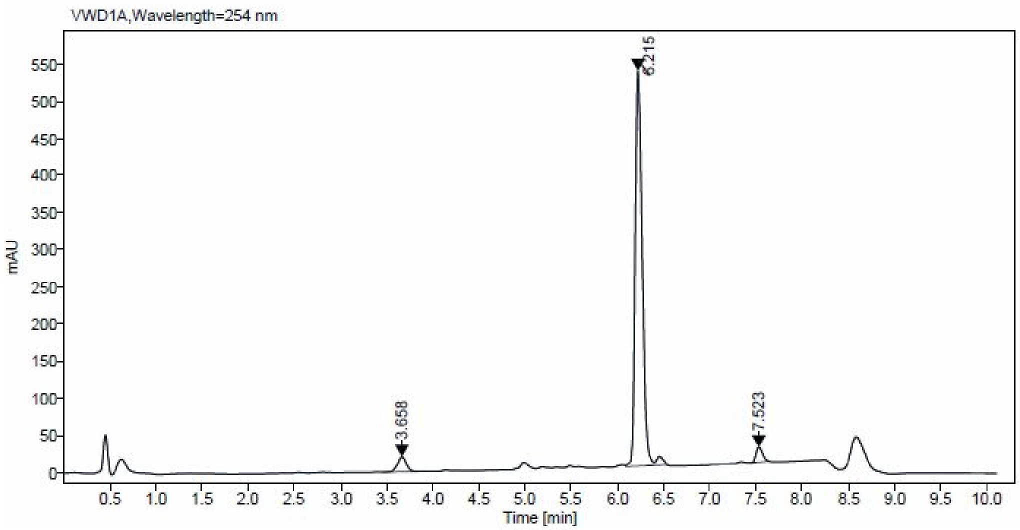

Signal: VWD1A,Wavelength=254 nm

RT [min]	Name	Area	Area%	Max Peak%	Height	Type	Width (50%)
7.523		95.4770	3.1639	3.419	19.862	MM m	
3.658		129.9320	4.3057	4.653	20.044	BB	
6.215		2792.2648	92.5304	100.000	532.042	MM m	
	**Sum**	**3017.6737**					

HPLC trace of Jun15191

Single Injection Report

**Data file:**	D:\CDSProjects\Checkout and Familiarization\Results\KL\Jun15191_HPLC.sirslt\Jun15191_HPLC.dx		
**Sample name:**	Jun15191_HPLC		
**Description:**			
**Sample amount:**	0.000	**Sample type:**	Sample
**Instrument:**	LCMS 1	**Location:**	P2-E4
**Injection date:**	2025-12-29 13:33:44-05:00	**Injection:**	1 of 1
**Acq. method:**	VP1 PROTAC_HPLC__method_10 min.amx	**Injection volume:**	5.000 µL
**Analysis method:**	3D UV Quantitative_DefaultMethod.pmx	**Acq. operator:**	SYSTEM
**Last changed:**	2023-03-22 11:19:18-04:00		

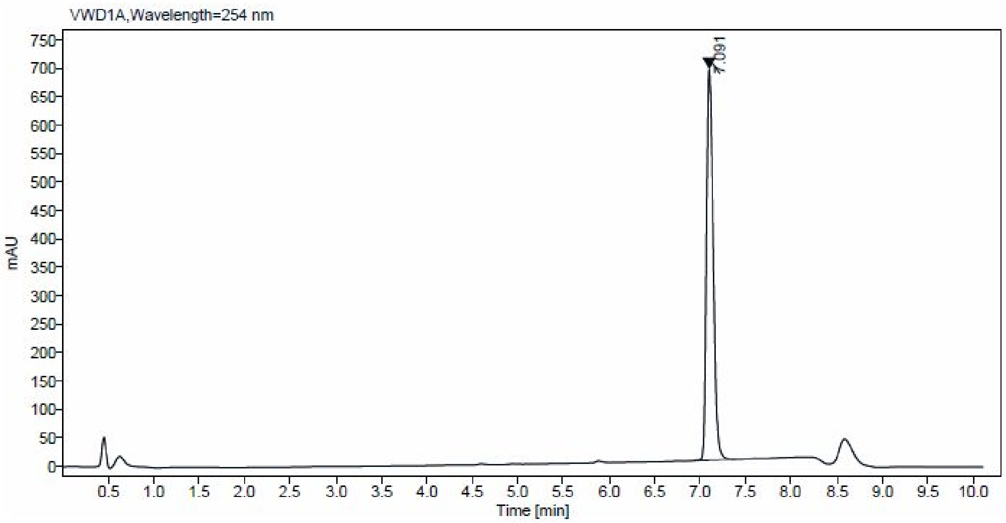

Signal: VWD1A,Wavelength=254 nm

RT [min]	Name	Area	Area%	Max Peak%	Height	Type	Width (50%)
7.091		3551.6122	100.0000	100.000	687.685	MM m	
	**Sum**	**3551.6122**					

HPLC trace of Jun15192

Single Injection Report Agilent

**Data file:**	D:\CDSProjects\Checkout and Familiarization\Results\KL\Jun15192_HPLC.sirslt\Jun15192_HPLC.dx		
**Sample name:**	Jun15192_HPLC		
**Description:**			
**Sample amount:**	0.000	**Sample type:**	Sample
**Instrument:**	LCMS 1	**Location:**	P2-E5
**Injection date:**	2025-12-29 13:44:41-05:00	**Injection:**	1 of 1
**Acq. method:**	VP1 PROTAC_HPLC__method_10 min.amx	**Injection volume:**	5.000 µL
**Analysis method:**	3D UV Quantitative_DefaultMethod.pmx	**Acq. operator:**	SYSTEM
**Last changed:**	2023-03-22 11:19:18-04:00		

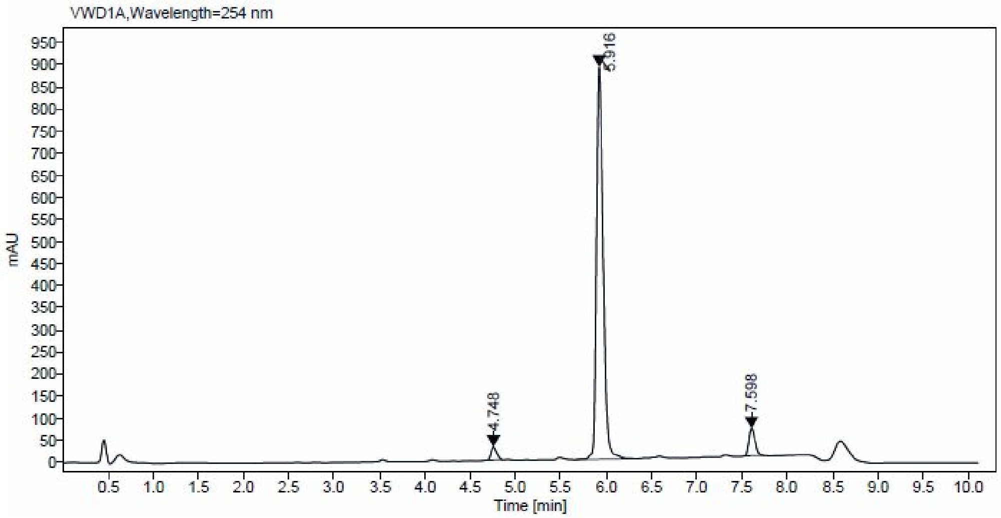

Signal: VWD1A,Wavelength=254 nm

RT [min]	Name	Area	Area%	Max Peak%	Height	Type	Width (50%)
4.748		126.7568	2.5387	2.768	28.739	MM m	
7.598		286.0944	5.7300	6.247	61.105	MM m	
5.916		4580.0550	91.7312	100.000	888.823	BB	
	**Sum**	**4992.9063**					

HPLC trace of Jun15294

Single Injection Report

**Data file:**	D:\CDSProjects\Checkout and Familiarization\Results\KL\Jun15294_HPLC.sirslt\Jun15294_HPLC.dx		
**Sample name:**	Jun15294_HPLC		
**Description:**			
**Sample amount:**	0.000	**Sample type:**	Sample
**Instrument:**	LCMS 1	**Location:**	P2-E8
**Injection date:**	2025-12-29 14:24:53-05:00	**Injection:**	1 of 1
**Acq. method:**	VP1 PROTAC_HPLC__method_10 min.amx	**Injection volume:**	5.000 µL
**Analysis method:**	3D UV Quantitative_DefaultMethod.pmx	**Acq. operator:**	SYSTEM
**Last changed:**	2023-03-22 11:19:18-04:00		

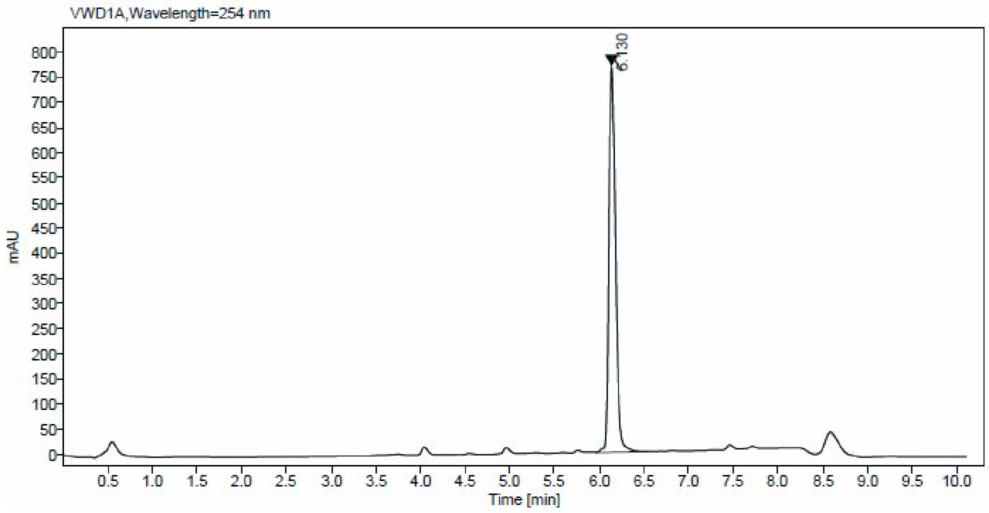

Signal: VWD1A,Wavelength=254 nm

RT [min]	Name	Area	Area%	Max Peak%	Height	Type	Width (50%)
6.130		3993.2966	100.0000	100.000	767.219	VV	
	**Sum**	**3993.2966**					

HPLC trace of Jun15322

Single Injection Report

Agilent

**Data file:**	D:\CDSProjects\Checkout and Familiarization\Results\KL\Jun15322_HPLC.sirslt\Jun15322_HPLC.dx		
**Sample name:**	Jun15322_HPLC		
**Description:**			
**Sample amount:**	0.000	**Sample type:**	Sample
**Instrument:**	LCMS 1	**Location:**	P2-E2
**Injection date:**	2025-12-29 13:11:48-05:00	**Injection:**	1 of 1
**Acq. method:**	VP1 PROTAC_HPLC__method_10 min.amx	**Injection volume:**	5.000 µL
**Analysis method:**	3D UV Quantitative_DefaultMethod.pmx	**Acq. operator:**	SYSTEM
**Last changed:**	2023-03-22 11:19:18-04:00		

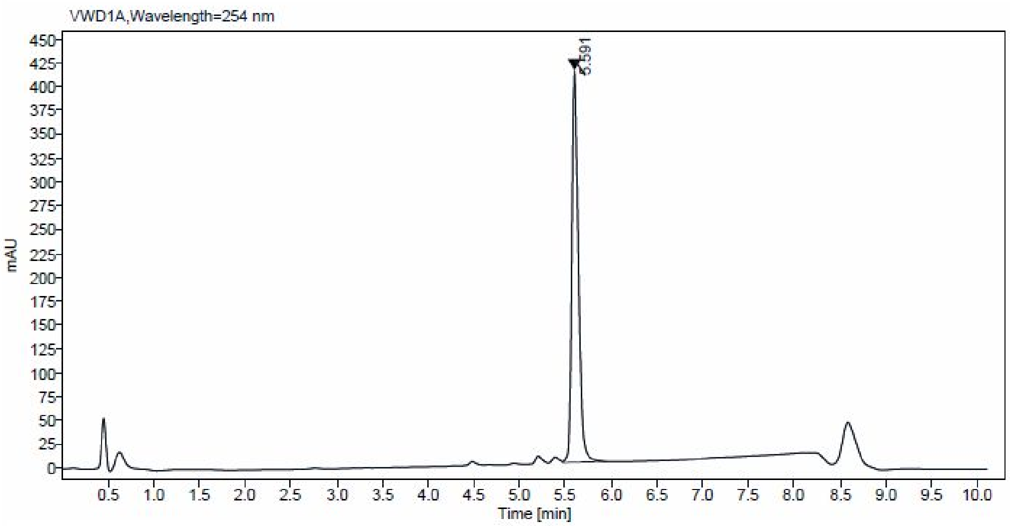

Signal: VWD1A,Wavelength=254 nm

RT [min]	Name	Area	Area%	Max Peak%	Height	Type	Width (50%)
5.591		2102.3880	100.0000	100.000	410.030	MM m	
	**Sum**	**2102.3880**					

HPLC trace of Jun15323

Single Injection Report Agilent

**Data file:**	D:\CDSProjects\Checkout and Familiarization\Results\KL\Jun15323_HPLC.sirslt\Jun15323_HPLC.dx		
**Sample name:**	Jun15323_HPLC		
**Description:**			
**Sample amount:**	0.000	**Sample type:**	Sample
**Instrument:**	LCMS 1	**Location:**	P2-E1
**Injection date:**	2025-12-29 13:00:49-05:00	**Injection:**	1 of 1
**Acq. method:**	VP1 PROTAC_HPLC__method_10 min.amx	**Injection volume:**	5.000 µL
**Analysis method:**	3D UV Quantitative_DefaultMethod.pmx	**Acq. operator:**	SYSTEM
**Last changed:**	2023-03-22 11:19:18-04:00		

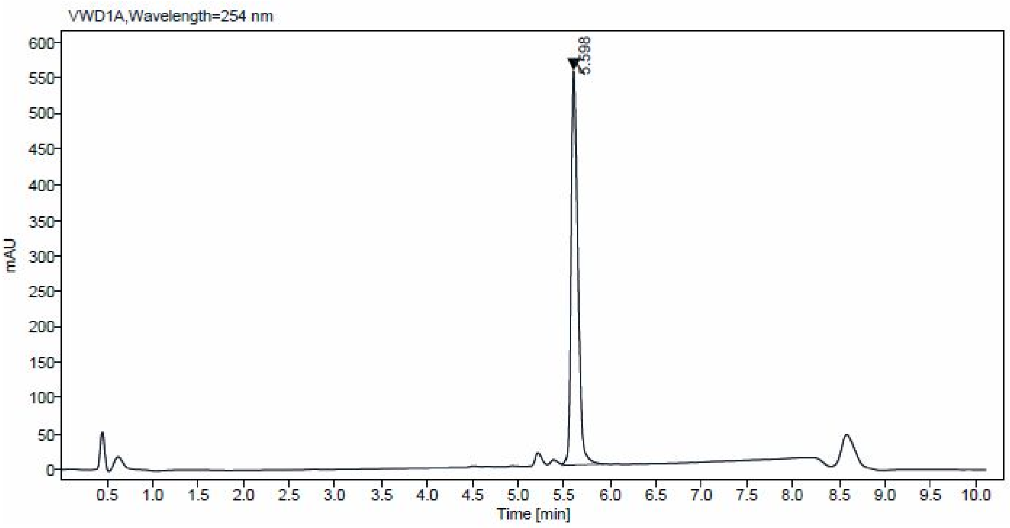

Signal: VWD1A,Wavelength=254 nm

RT [min]	Name	Area	Area%	Max Peak%	Height	Type	Width (50%)
5.598		2849.6738	100.0000	100.000	554.666	MM m	
	**Sum**	**2849.6738**					

HPLC trace of Jun15331

Single Injection Report Agilent

**Data file:**	D:\CDSProjects\Checkout and Familiarization\Results\KL\Jun15331_HPLC.sirslt\Jun15331_HPLC.dx		
**Sample name:**	Jun15331_HPLC		
**Description:**			
**Sample amount:**	0.000	**Sample type:**	Sample
**Instrument:**	LCMS 1	**Location:**	P2-D9
**Injection date:**	2025-12-29 12:27:52-05:00	**Injection:**	1 of 1
**Acq. method:**	VP1 PROTAC_HPLC__method_10 min.amx	**Injection volume:**	5.000 µL
**Analysis method:**	3D UV Quantitative_DefaultMethod.pmx	**Acq. operator:**	SYSTEM
**Last changed:**	2023-03-22 11:19:18-04:00		

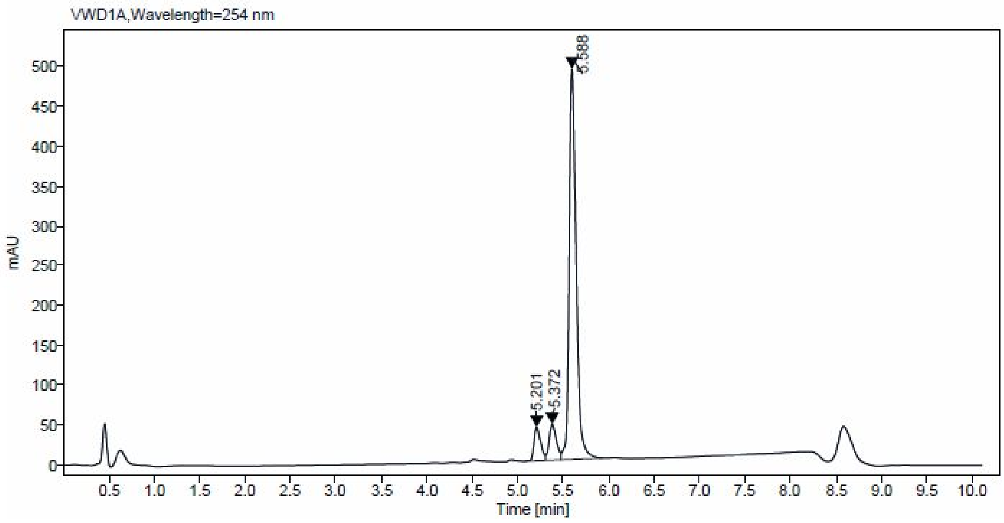

Signal: VWD1A,Wavelength=254 nm

RT [min]	Name	Area	Area%	Max Peak%	Height	Type	Width (50%)
5.201		213.2692	6.8570	8.062	41.807	BV	
5.372		251.4686	8.0852	9.506	44.889	VV	
5.588		2645.5017	85.0578	100.000	490.741	VB	
	**Sum**	**3110.2395**					

HPLC trace of Jun15332

Single Injection Report Agilent

**Data file:**	D:\CDSProjects\Checkout and Familiarization\Results\KL\Jun15332_HPLC.sirslt\Jun15332_HPLC.dx		
**Sample name:**	Jun15332_HPLC		
**Description:**			
**Sample amount:**	0.000	**Sample type:**	Sample
**Instrument:**	LCMS 1	**Location:**	P2-D10
**Injection date:**	2025-12-29 12:38:51-05:00	**Injection:**	1 of 1
**Acq. method:**	VP1 PROTAC_HPLC__method_10 min.amx	**Injection volume:**	5.000 µL
**Analysis method:**	3D UV Quantitative_DefaultMethod.pmx	**Acq. operator:**	SYSTEM
**Last changed:**	2023-03-22 11:19:18-04:00		

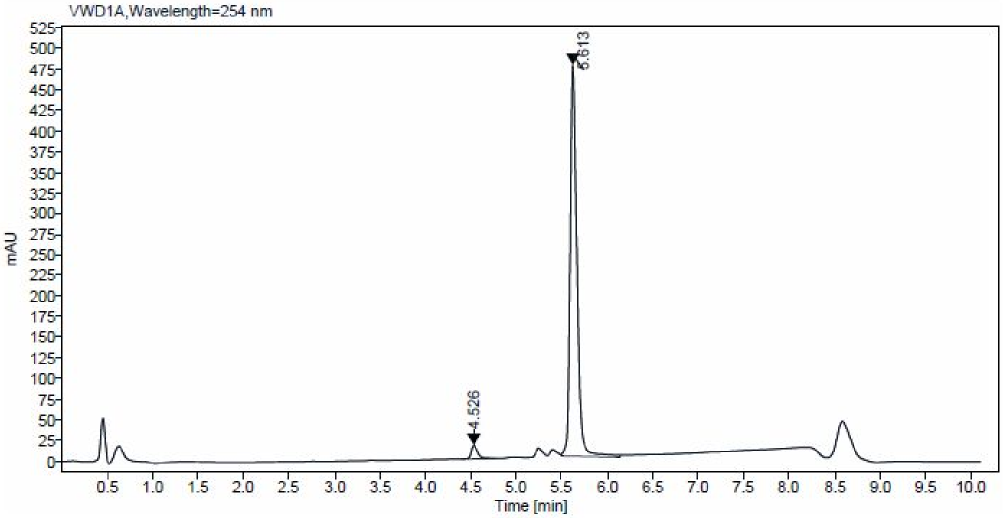

Signal: VWD1A,Wavelength=254 nm

RT [min]	Name	Area	Area%	Max Peak%	Height	Type	Width (50%)
4.526		80.0740	3.0752	3.173	15.978	BB	
5.613		2523.7758	96.9248	100.000	473.537	VV	
	**Sum**	**2603.8498**					

HPLC trace of Jun15333

Single Injection Report

**Data file:**	D:\CDSProjects\Checkout and Familiarization\Results\KL\Jun15333_HPLC.sirslt\Jun15333_HPLC.dx		
**Sample name:**	Jun15333_HPLC		
**Description:**			
**Sample amount:**	0.000	**Sample type:**	Sample
**Instrument:**	LCMS 1	**Location:**	P2-D8
**Injection date:**	2025-12-29 12:16:54-05:00	**Injection:**	1 of 1
**Acq. method:**	VP1 PROTAC_HPLC__method_10 min.amx	**Injection volume:**	5.000 µL
**Analysis method:**	3D UV Quantitative_DefaultMethod.pmx	**Acq. operator:**	SYSTEM
**Last changed:**	2023-03-22 11:19:18-04:00		

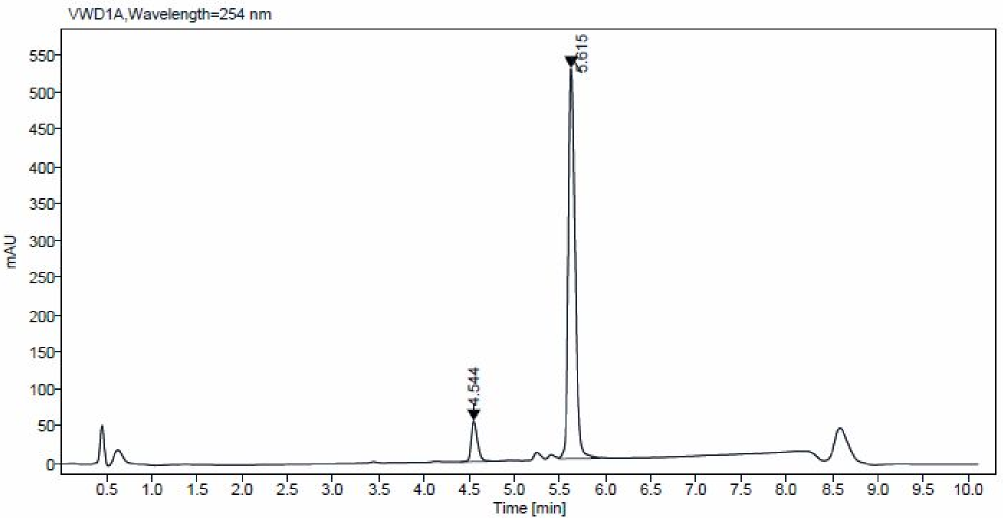

Signal: VWD1A,Wavelength=254 nm

RT [min]	Name	Area	Area%	Max Peak%	Height	Type	Width (50%)
4.544		262.0625	8.8145	9.667	54.163	BV	
5.615		2711.0350	91.1855	100.000	526.232	VV	
	**Sum**	**2973.0974**					

HPLC trace of Jun15373

Single Injection Report

**Data file:**	D:\CDSProjects\Checkout and Familiarization\Results\KL\Jun15373_HPLC.sirslt\Jun15373_HPLC.dx		
**Sample name:**	Jun15373_HPLC		
**Description:**			
**Sample amount:**	0.000	**Sample type:**	Sample
**Instrument:**	LCMS 1	**Location:**	P2-E6
**Injection date:**	2025-12-29 13:55:39-05:00	**Injection:**	1 of 1
**Acq. method:**	VP1 PROTAC_HPLC__method_10 min.amx	**Injection volume:**	5.000 µL
**Analysis method:**	3D UV Quantitative_DefaultMethod.pmx	**Acq. operator:**	SYSTEM
**Last changed:**	2023-03-22 11:19:18-04:00		

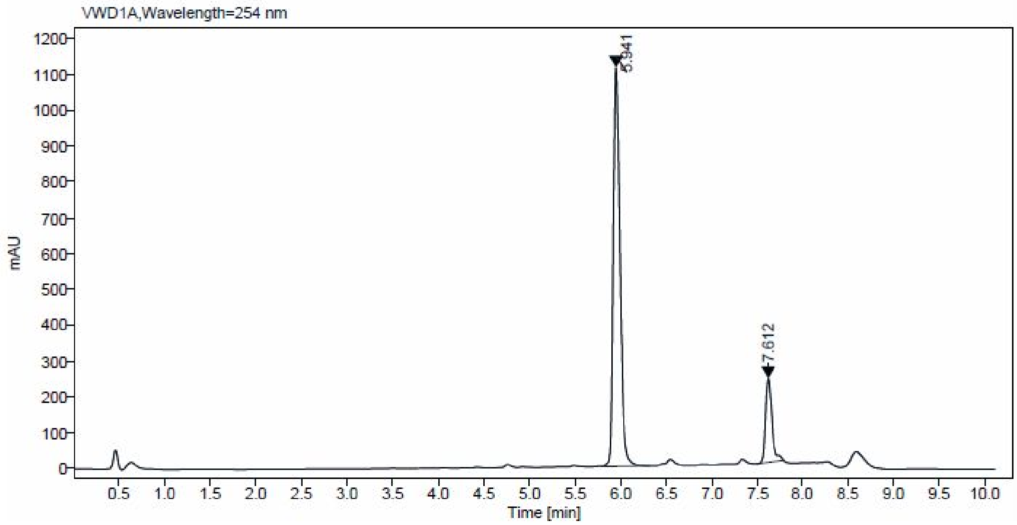

Signal: VWD1A,Wavelength=254 nm

RT [min]	Name		Area	Area%	Max Peak%	Height	Type	Width (50%)
7.612			1135.7816	16.8024	20.196	233.612	BM m	
5.941			5623.8578	83.1976	100.000	1112.817	VB	
		**Sum**	**6759.6394**					

HPLC trace of Jun15374

Single Injection Report Agilent

**Data file:**	D:\CDSProjects\Checkout and Familiarization\Results\KL\Jun15374_HPLC.sirslt\Jun15374_HPLC.dx		
**Sample name:**	Jun15374_HPLC		
**Description:**			
**Sample amount:**	0.000	**Sample type:**	Sample
**Instrument:**	LCMS 1	**Location:**	P2-E7
**Injection date:**	2025-12-29 14:06:35-05:00	**Injection:**	1 of 1
**Acq. method:**	VP1 PROTAC_HPLC__method_10 min.amx	**Injection volume:**	5.000 µL
**Analysis method:**	3D UV Quantitative_DefaultMethod.pmx	**Acq. operator:**	SYSTEM
**Last changed:**	2023-03-22 11:19:18-04:00		

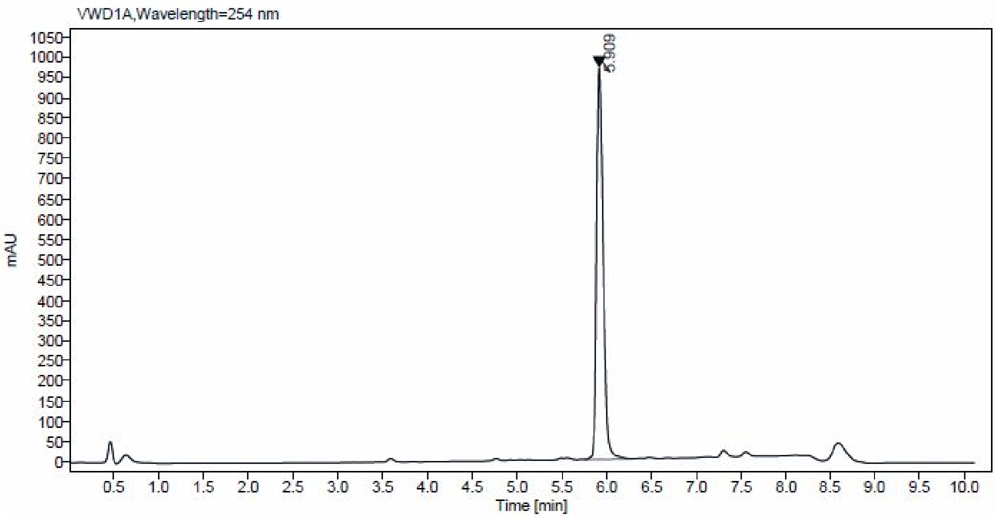

Signal: VWD1A,Wavelength=254 nm

RT [min]	Name	Area	Area%	Max Peak%	Height	Type	Width (50%)
5.909		4967.8101	100.0000	100.000	966.913	VV	
	**Sum**	**4967.8101**					

HPLC trace of Jun15375

Single Injection Report Agilent

**Data file:**	D:\CDSProjects\Checkout and Familiarization\Results\KL\Jun15375_HPLC.sirslt\Jun15375_HPLC.dx		
**Sample name:**	Jun15375_HPLC		
**Description:**			
**Sample amount:**	0.000	**Sample type:**	Sample
**Instrument:**	LCMS 1	**Location:**	P2-D11
**Injection date:**	2025-12-29 12:49:50-05:00	**Injection:**	1 of 1
**Acq. method:**	VP1 PROTAC_HPLC__method_10 min.amx	**Injection volume:**	5.000 µL
**Analysis method:**	3D UV Quantitative_DefaultMethod.pmx	**Acq. operator:**	SYSTEM
**Last changed:**	2023-03-22 11:19:18-04:00		

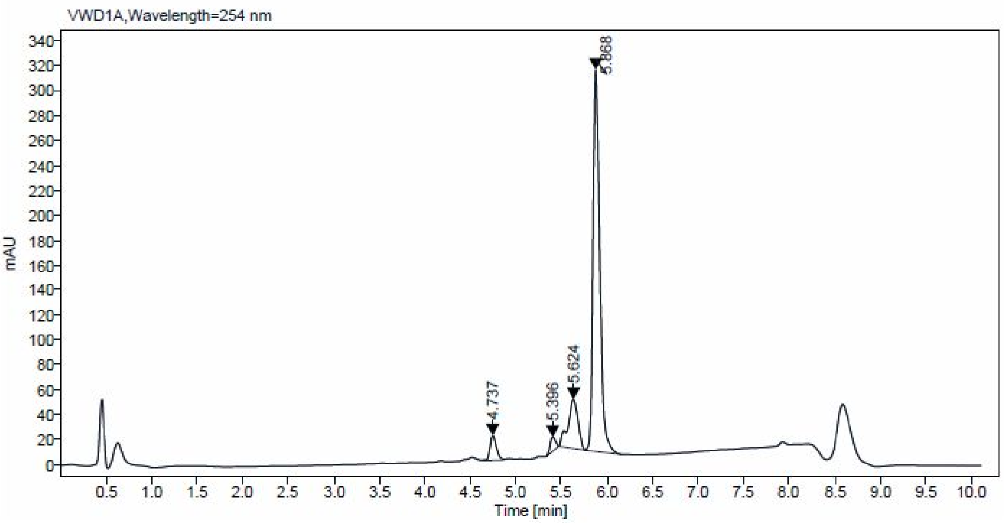

Signal: VWD1A,Wavelength=254 nm

RT [min]	Name	Area	Area%	Max Peak%	Height	Type	Width (50%)
5.396		44.1863	2.1724	2.776	11.162	BM m	
4.737		96.8938	4.7637	6.087	19.727	VV	
5.624		301.1893	14.8077	18.922	39.223	MB m	
5.868		1591.7403	78.2563	100.000	305.643	BB	
	**Sum**	**2034.0097**					

HPLC trace of Jun15412

Single Injection Report Agilent

**Data file:**	D:\CDSProjects\Checkout and Familiarization\Results\KL\Jun15412_HPLC.sirslt\Jun15412_HPLC.dx		
**Sample name:**	Jun15412_HPLC		
**Description:**			
**Sample amount:**	0.000	**Sample type:**	Sample
**Instrument:**	LCMS 1	**Location:**	P2-D4
**Injection date:**	2025-12-29 11:33:06-05:00	**Injection:**	1 of 1
**Acq. method:**	VP1 PROTAC_HPLC__method_10 min.amx	**Injection volume:**	5.000 µL
**Analysis method:**	3D UV Quantitative_DefaultMethod.pmx	**Acq. operator:**	SYSTEM
**Last changed:**	2023-03-22 11:19:18-04:00		

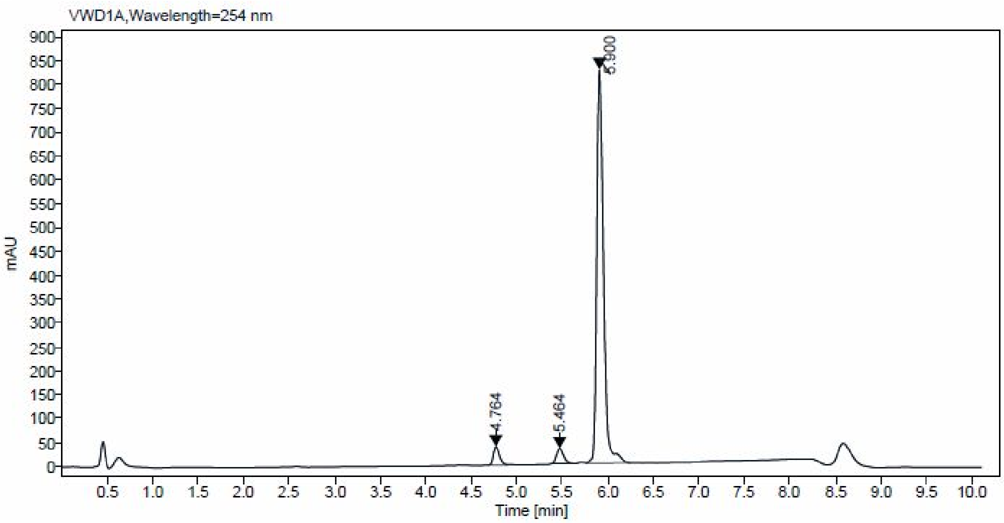

Signal: VWD1A,Wavelength=254 nm

RT [min]	Name	Area	Area%	Max Peak%	Height	Type	Width (50%)
5.464		168.8290	3.6275	3.923	30.559	MB m	
4.764		181.9551	3.9095	4.228	38.074	MV m	
5.900		4303.3692	92.4630	100.000	823.339	MM m	
	**Sum**	**4654.1533**					

HPLC trace of Jun15551

Single Injection Report

**Data file:**	D:\CDSProjects\Checkout and Familiarization\Results\KL\Jun15551_HPLC.sirslt\Jun15551_HPLC.dx		
**Sample name:**	Jun15551_HPLC		
**Description:**			
**Sample amount:**	0.000	**Sample type:**	Sample
**Instrument:**	LCMS 1	**Location:**	P2-E9
**Injection date:**	2025-12-29 14:57:21-05:00	**Injection:**	1 of 1
**Acq. method:**	VP1 PROTAC_HPLC__method_10 min.amx	**Injection volume:**	5.000 µL
**Analysis method:**	3D UV Quantitative_DefaultMethod.pmx	**Acq. operator:**	SYSTEM
**Last changed:**	2023-03-22 11:19:18-04:00		

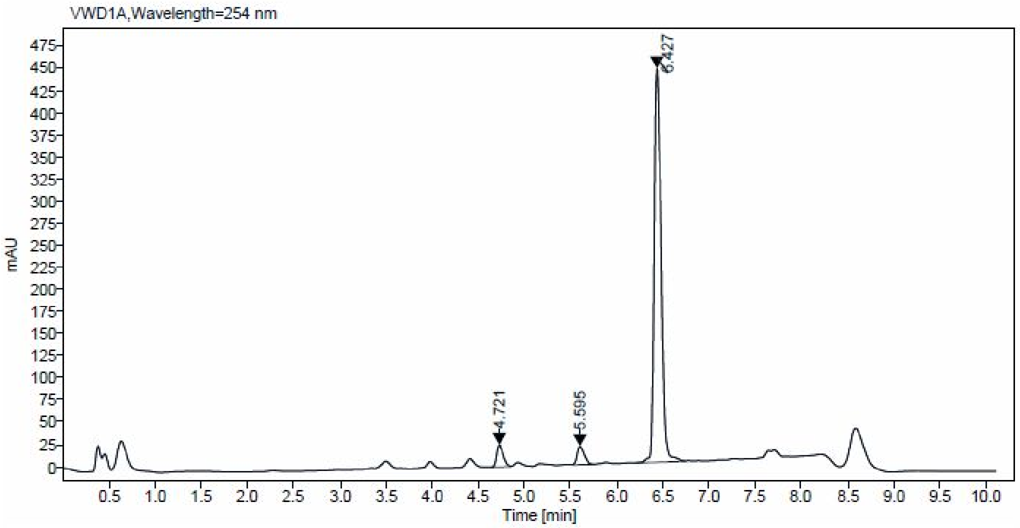

Signal: VWD1A,Wavelength=254 nm

RT [min]	Name	Area	Area%	Max Peak%	Height	Type	Width (50%)
5.595		113.7103	4.3678	4.808	20.546	VB	
4.721		124.7759	4.7928	5.276	24.788	BV	
6.427		2364.9158	90.8394	100.000	444.706	VV	
	**Sum**	**2603.4020**					

HPLC trace of Jun15616

Single Injection Report

Agilent

**Data file:**	D:\CDSProjects\Checkout and Familiarization\Results\KL\Jun15616_HPLC.sirslt\Jun15616_HPLC.dx		
**Sample name:**	Jun15616_HPLC		
**Description:**			
**Sample amount:**	0.000	**Sample type:**	Sample
**Instrument:**	LCMS 1	**Location:**	P2-F1
**Injection date:**	2025-12-29 15:51:49-05:00	**Injection:**	1 of 1
**Acq. method:**	VP1 PROTAC_HPLC__method_10 min.amx	**Injection volume:**	5.000 µL
**Analysis method:**	3D UV Quantitative_DefaultMethod.pmx	**Acq. operator:**	SYSTEM
**Last changed:**	2023-03-22 11:19:18-04:00		

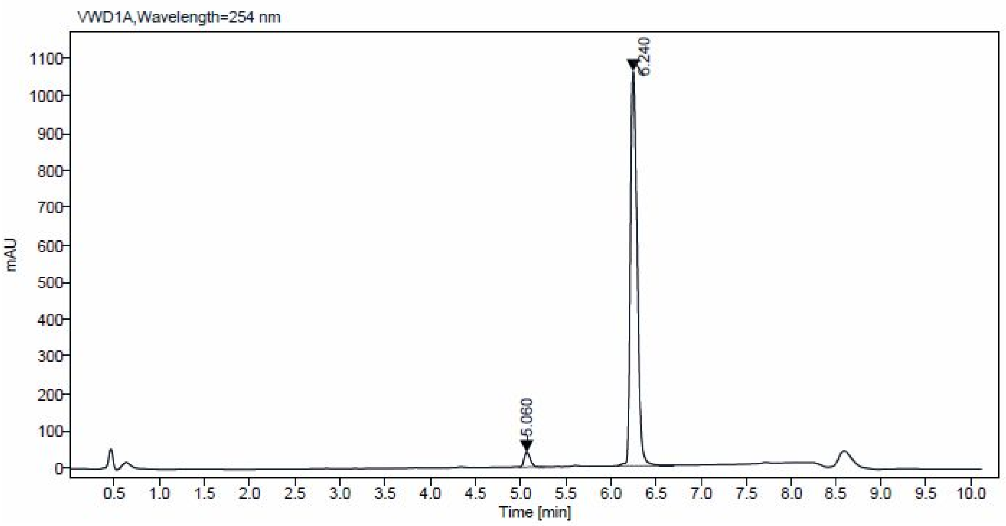

Signal: VWD1A,Wavelength=254 nm

RT [min]	Name	Area	Area%	Max Peak%	Height	Type	Width (50%)
5.060		193.2719	3.4004	3.520	39.993	VB	
6.240		5490.5264	96.5996	100.000	1060.420	MM m	
	**Sum**	**5683.7984**					

HPLC trace of Jun15692

Single Injection Report

**Data file:**	D:\CDSProjects\Checkout and Familiarization\Results\KL\Jun15692_HPLC_2.sirslt\Jun15692_HPLC_2.dx		
**Sample name:**	Jun15692_HPLC_2		
**Description:**			
**Sample amount:**	0.000	**Sample type:**	Sample
**Instrument:**	LCMS 1	**Location:**	P2-D5
**Injection date:**	2025-12-30 10:45:30-05:00	**Injection:**	1 of 1
**Acq. method:**	VP1 PROTAC_HPLC__method_10 min.amx	**Injection volume:**	5.000 µL
**Analysis method:**	3D UV Quantitative_DefaultMethod.pmx	**Acq. operator:**	SYSTEM
**Last changed:**	2023-03-22 11:19:18-04:00		

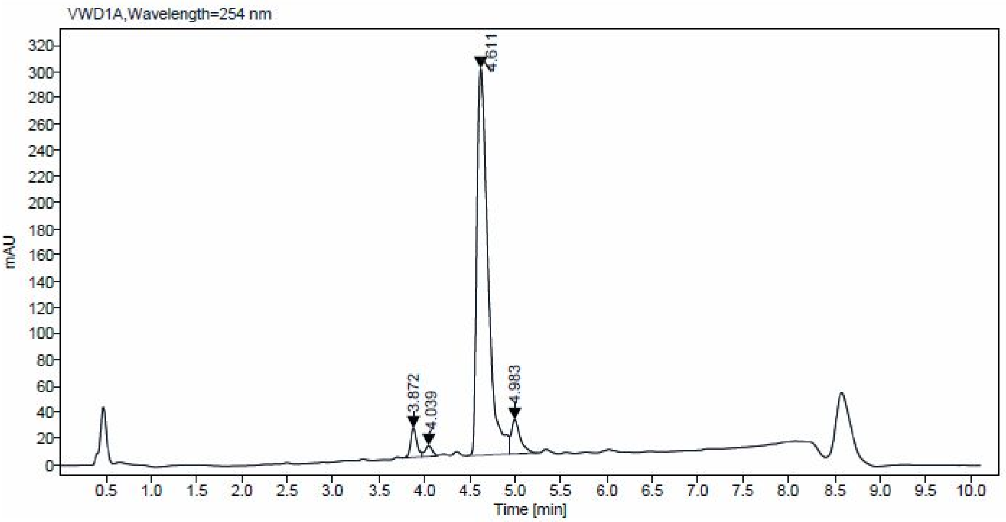

Signal: VWD1A,Wavelength=254 nm

RT [min]	Name	Area	Area%	Max Peak%	Height	Type	Width (50%)
4.039		48.5740	1.7220	1.957	8.294	VV	
3.872		108.6137	3.8505	4.377	22.323	BV	
4.983		182.0911	6.4554	7.338	26.082	VB	
4.611		2481.4694	87.9720	100.000	295.451	BM m	
	**Sum**	**2820.7481**					

HPLC trace of Jun15693

Single Injection Report Agilent

**Data file:**	D:\CDSProjects\Checkout and Familiarization\Results\KL\Jun15693_HPLC.sirslt\Jun15693_HPLC.dx		
**Sample name:**	Jun15693_HPLC		
**Description:**			
**Sample amount:**	0.000	**Sample type:**	Sample
**Instrument:**	LCMS 1	**Location:**	P2-F3
**Injection date:**	2025-12-29 16:13:48-05:00	**Injection:**	1 of 1
**Acq. method:**	VP1 PROTAC_HPLC__method_10 min.amx	**Injection volume:**	5.000 µL
**Analysis method:**	3D UV Quantitative_DefaultMethod.pmx	**Acq. operator:**	SYSTEM
**Last changed:**	2023-03-22 11:19:18-04:00		

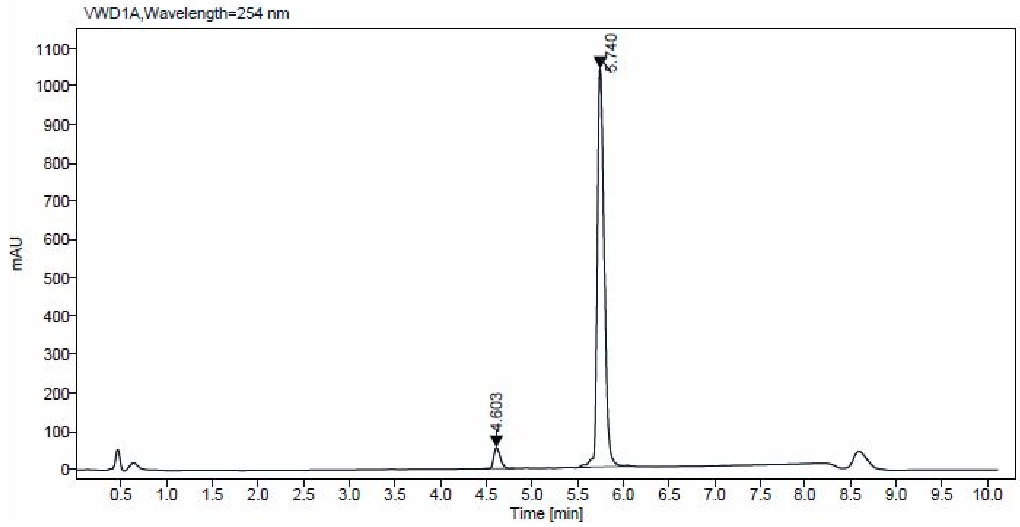

Signal: VWD1A,Wavelength=254 nm

RT [min]	Name	Area	Area%	Max Peak%	Height	Type	Width (50%)
4.603		266.1250	4.6449	4.871	54.902	BB	
5.740		5463.2172	95.3551	100.000	1040.003	MM m	
**Sum**		**5729.3422**					

HPLC trace of Jun15701

Single Injection Report Agilent

**Data file:**	D:\CDSProjects\Checkout and Familiarization\Results\KL\Jun15701_HPLC.sirslt\Jun15701_HPLC.dx		
**Sample name:**	Jun15701_HPLC		
**Description:**			
**Sample amount:**	0.000	**Sample type:**	Sample
**Instrument:**	LCMS 1	**Location:**	P2-F2
**Injection date:**	2025-12-29 16:02:49-05:00	**Injection:**	1 of 1
**Acq. method:**	VP1 PROTAC_HPLC__method_10 min.amx	**Injection volume:**	5.000 µL
**Analysis method:**	3D UV Quantitative_DefaultMethod.pmx	**Acq. operator:**	SYSTEM
**Last changed:**	2023-03-22 11:19:18-04:00		

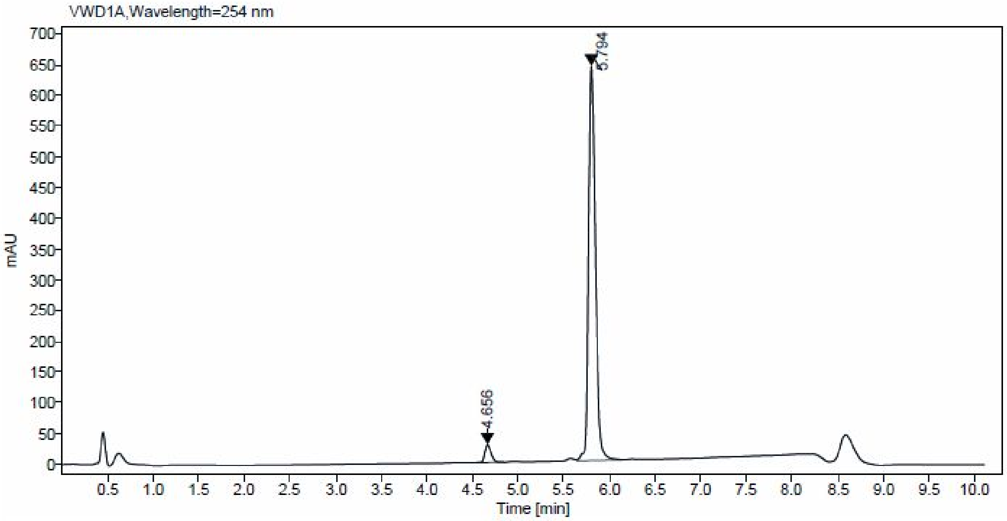

Signal: VWD1A,Wavelength=254 nm

RT [min]	Name	Area	Area%	Max Peak%	Height	Type	Width (50%)
4.656		136.8745	3.9610	4.124	28.251	BV	
5.794		3318.6511	96.0390	100.000	641.466	VV	
	**Sum**	**3455.5256**					

HPLC trace of Jun15702

Single Injection Report

Agilent

**Data file:**	D:\CDSProjects\Checkout and Familiarization\Results\KL\Jun15702_HPLC_2.sirslt\Jun15702_HPLC_2.dx		
**Sample name:**	Jun15702_HPLC_2		
**Description:**			
**Sample amount:**	0.000	**Sample type:**	Sample
**Instrument:**	LCMS 1	**Location:**	P2-D6
**Injection date:**	2025-12-30 11:08:37-05:00	**Injection:**	1 of 1
**Acq. method:**	VP1 PROTAC_HPLC__method_10 min.amx	**Injection volume:**	5.000 µL
**Analysis method:**	3D UV Quantitative_DefaultMethod.pmx	**Acq. operator:**	SYSTEM
**Last changed:**	2023-03-22 11:19:18-04:00		

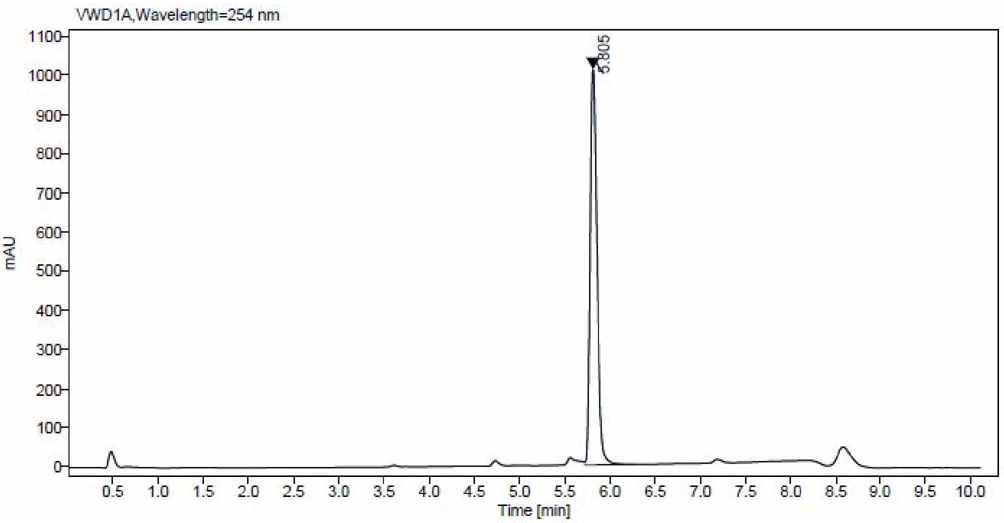

Signal: VWD1A,Wavelength=254 nm

RT [min]	Name	Area	Area%	Max Peak%	Height	Type	Width (50%)
5.805		5272.9559	100.0000	100.000	1010.860	VB	
	**Sum**	**5272.9559**					

HPLC trace of Jun15953

Single Injection Report Agilent

**Data file:**	D:\CDSProjects\Checkout and Familiarization\Results\KL\Jun15953_HPLC_2.sirslt\Jun15953_HPLC_2.dx		
**Sample name:**	Jun15953_HPLC_2		
**Description:**			
**Sample amount:**	0.000	**Sample type:**	Sample
**Instrument:**	LCMS 1	**Location:**	P2-D7
**Injection date:**	2025-12-29 12:05:56-05:00	**Injection:**	1 of 1
**Acq. method:**	VP1 PROTAC_HPLC__method_10 min.amx	**Injection volume:**	5.000 µL
**Analysis method:**	3D UV Quantitative_DefaultMethod.pmx	**Acq. operator:**	SYSTEM
**Last changed:**	2023-03-22 11:19:18-04:00		

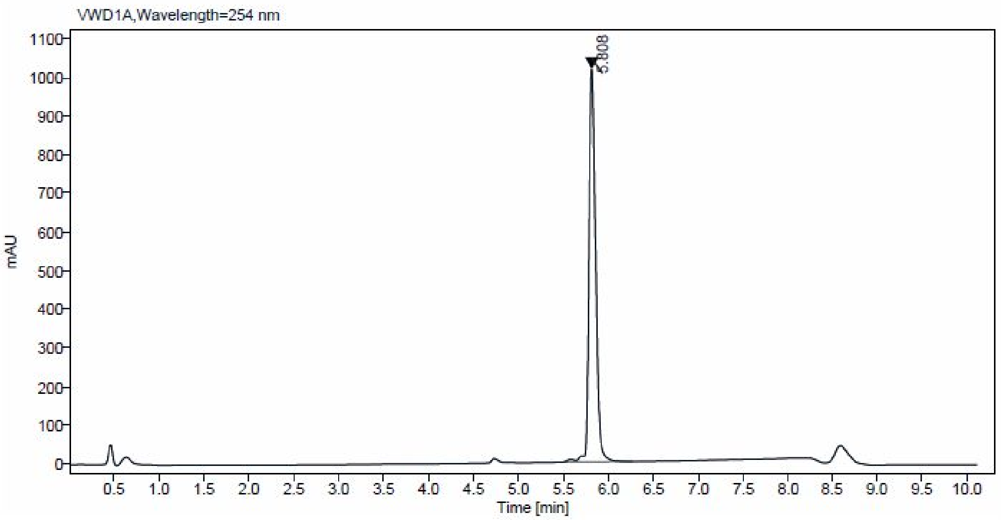

Signal: VWD1A,Wavelength=254 nm

RT [min]	Name	Area	Area%	Max Peak%	Height	Type	Width (50%)
5.808		5264.4291	100.0000	100.000	1015.261	MB m	
	**Sum**	**5264.4291**					

Reference

(1) Repetto, G.; del Peso, A.; Zurita, J. L., Neutral red uptake assay for the estimation of cell viability/cytotoxicity. *Nature Protocols* **2008,** *3*, 1125-1131.

(2) Tan, B.; Sacco, M.; Tan, H.; Li, K.; Joyce, R.; Zhang, X.; Chen, Y.; Wang, J., Exploring diverse reactive warheads for the design of SARS-CoV-2 main protease inhibitors. *European Journal of Medicinal Chemistry* **2023,** *259*, 115667.

(3) Guzman, C.; Bagga, M.; Kaur, A.; Westermarck, J.; Abankwa, D., ColonyArea: an ImageJ plugin to automatically quantify colony formation in clonogenic assays. *PLoS One* **2014,** *9*, e92444.

(4) Ma, C.; Hu, Y.; Zhang, J.; Musharrafieh, R.; Wang, J., A Novel Capsid Binding Inhibitor Displays Potent Antiviral Activity against Enterovirus D68. *ACS Infect Dis* **2019,** *5*, 1952-1962.
